# Supplementary material for: Improved microbial genomes and gene catalog of the chicken gut from metagenomic sequencing of high-fidelity long reads
Source: Gigascience. 2022 Nov 18;11:giac116. doi: 10.1093/gigascience/giac116 (PMC9673493; doi:10.1093/gigascience/giac116)
Supplement: giac116_GIGA-D-22-00175_Original_Submission [file giac116_giga-d-22-00175_original_submission.pdf]

GigaScience

Improved microbial genomes and gene catalog of chicken gut from metagenomic sequencing of high-fidelity long reads

--Manuscript Draft--

**Manuscript Number:**	GIGA-D-22-00175
**Full Title:**	Improved microbial genomes and gene catalog of chicken gut from metagenomic sequencing of high-fidelity long reads
**Article Type:**	Research
**Funding Information:**	National Natural Science Foundation of China (Grant No. 32000408) — Dr. Yan Zhang
**Abstract:**	Background<br><br>Due to the importance of chicken production and the remarkable influence of gut microbiota on the host's health and growth, tens of thousands of metagenome-assembled genomes (MAGs) have been constructed for the chicken gut microbiome. However, limited by the short-read sequencing and assembly technologies, most of these MAGs are far from complete and have lower qualities with contamination.<br><br>Results<br><br>We generated 332 Gb high-fidelity (HiFi) long reads from the five chicken intestinal compartments, and assembled 461 and 337 microbial genomes at species and strain level, of which 53% and 55% are circular genomes, respectively. For the assembled microbial genomes, about 95% were regarded as complete according to the "RNA complete" criteria, which requires at least one full-length rRNA operon coding for all three types of rRNA(16S, 23S and 5S rRNA) and at least 18 copies of full-length tRNA genes. In comparison with the short-read derived chicken MAGs, 384 (83% of 461) and 89 (26% of 337) strain-level and species-level genomes in this study are novel and do not have any matches. At the gene level, a third of the 2.5 million genes in HiFi derived gene catalog are novel and cannot be matched to the short-read derived gene catalogs. Moreover, the HiFi derived genomes have much higher continuity and completeness, as well as lower contamination; the HiFi derived gene catalog has much higher ratio of complete gene structures. The dominant phyla in our HiFi assembled genomes is Firmicutes (82.5%), and the foregut is highly enriched in five genera Ligilactobacillus , Limosilactobacillus , Lactobacillus , Weissella , and Enterococcus , all of which belongs to the order Lactobacillales. Using GTDB-tk, all the 337 species-level genomes were successfully classified to order level, however, 2, 35 and 189 genomes could not be classified into any known family, genus, and species, respectively. Among these not-fully classified genomes, 9 and 49 of them may belong to novel genera and species, respectively, for that their 16S-rRNA genes have identities lower than 95% and 97% to any known 16S-rRNA genes.<br><br>Conclusions<br><br>HiFi sequencing not only remarkably improved the quality of metagenome assemblies and gene structures, but also recovered a substantial portion of novel genomes and genes that were missed in short-read metagenome studies. The novel genomes or novel species obtained in this study will facilitate the gut microbiome and host-microbiota interaction studies, which would benefit the sustainable development of poultry.
**Corresponding Author:**	Wei Fan<br>Chinese Academy of Agricultural Sciences<br>shenzhen, guangdong CHINA
**Corresponding Author Secondary Information:**	
**Corresponding Author's Institution:**	Chinese Academy of Agricultural Sciences

Powered by Editorial Manager® and ProduXion Manager® from Aries Systems Corporation

Corresponding Author's Secondary Institution:	
First Author:	Wei Fan
First Author Secondary Information:	
Order of Authors:	Wei Fan
	Yan Zhang
	Fan Jiang
	Boyuan Yang
	Sen Wang
	Hengchao Wang
	Anqi Wang
	Dong Xu
Order of Authors Secondary Information:	
Additional Information:	
**Question**	**Response**
Are you submitting this manuscript to a special series or article collection?	No
**Experimental design and statistics**<br><br>Full details of the experimental design and statistical methods used should be given in the Methods section, as detailed in our Minimum Standards Reporting Checklist. Information essential to interpreting the data presented should be made available in the figure legends.<br><br>Have you included all the information requested in your manuscript?	Yes
**Resources**<br><br>A description of all resources used, including antibodies, cell lines, animals and software tools, with enough information to allow them to be uniquely identified, should be included in the Methods section. Authors are strongly encouraged to cite Research Resource Identifiers (RRIDs) for antibodies, model organisms and tools, where possible.<br><br>Have you included the information	Yes

requested as detailed in our Minimum Standards Reporting Checklist?	
**Availability of data and materials**<br><br>All datasets and code on which the conclusions of the paper rely must be either included in your submission or deposited in publicly available repositories (where available and ethically appropriate), referencing such data using a unique identifier in the references and in the “Availability of Data and Materials” section of your manuscript.<br><br>Have you have met the above requirement as detailed in our Minimum Standards Reporting Checklist?	Yes

maintext include tables and figures

Click here to access/download;Manuscript;manuscript_v1-20220703-final.docx

Improved microbial genomes and gene catalog of chicken gut from metagenomic sequencing of high-fidelity long reads

Yan Zhang*, Fan Jiang*, Boyuan Yang*, Sen Wang, Hengchao Wang, Anqi Wang, Dong Xu, and Wei Fan

Guangdong Laboratory for Lingnan Modern Agriculture (Shenzhen Branch), Genome Analysis Laboratory of the Ministry of Agriculture and Rural Affairs, Agricultural Genomics Institute at Shenzhen, Chinese Academy of Agricultural Sciences, Shenzhen, Guangdong, 518120, China.

*These authors contributed equally to this work. Correspondence should be addressed to fanwei@caas.cn.

Abstract

Background: Due to the importance of chicken production and the remarkable influence of gut microbiota on the host's health and growth, tens of thousands of metagenome-assembled genomes (MAGs) have been constructed for the chicken gut microbiome. However, limited by the short-read sequencing and assembly technologies, most of these MAGs are far from complete and have lower qualities with contamination.

Results: We generated 332 Gb high-fidelity (HiFi) long reads from the five chicken intestinal compartments, and assembled 461 and 337 microbial genomes at species and strain level, of which 53% and 55% are circular genomes, respectively. For the assembled microbial genomes, about 95% were regarded as complete according to the "RNA complete" criteria, which requires at least one full-length rRNA operon coding for all three types of rRNA(16S, 23S and 5S rRNA) and at least 18 copies of full-length tRNA genes. In comparison with the short-read derived chicken MAGs, 384 (83% of 461) and 89 (26% of 337) strain-level and species-level genomes in this study are novel and do not have any matches. At the gene level, a third of the 2.5 million genes in HiFi derived gene catalog are novel and cannot be matched to the short-read derived gene catalogs. Moreover, the HiFi derived genomes have much higher continuity and completeness, as well as lower contamination; the HiFi derived gene catalog has much higher ratio of complete gene structures. The dominant phyla in our HiFi assembled genomes is Firmicutes (82.5%), and the foregut is highly enriched in five genera *Ligilactobacillus*, *Limosilactobacillus*, *Lactobacillus*, *Weissella*, and *Enterococcus*, all

of which belongs to the order Lactobacillales. Using GTDB-tk, all the 337 species-level genomes were successfully classified to order level, however, 2, 35 and 189 genomes could not be classified into any known family, genus, and species, respectively. Among these not-fully classified genomes, 9 and 49 of them may belong to novel genera and species, respectively, for that their 16S-rRNA genes have identities lower than 95% and 97% to any known 16S-rRNA genes.
Conclusions: HiFi sequencing not only remarkably improved the quality of metagenome assemblies and gene structures, but also recovered a substantial portion of novel genomes and genes that were missed in short-read metagenome studies. The novel genomes or novel species obtained in this study will facilitate the gut microbiome and host-microbiota interaction studies, which would benefit the sustainable development of poultry.

Introduction

Domestic chicken, *Gallus gallus*, has long been used as a model animal for avian species, and chicken eggs and meat provide a primary source of animal-derived protein in the human diet. The first draft genome sequence of chicken was published in 2004, providing unique perspectives on vertebrate evolution (International Chicken Genome Sequencing, 2004).Then, the population resequencing studies revealed not only the phylogeny history and population structure, but also the loci selection during chicken domestication (Rubin, et al., 2010; Wong, et al., 2004). The gut microbiota can degrade dietary polysaccharides, detoxify xenobiotics, produce nutrients and energies such as vitamins, amino acids, short chain fatty acids (SCFA), and can also modulate the immune system, thus play important roles in chicken nutrition, physiology, immunity, and health. However, the gut microbiota also contain many zoonotic pathogens, posing threats to the poultry industry and human health (Oakley, et al., 2014; Yeoman, et al., 2012). Due to the importance of chicken gut microbiota, their compositions and interactions with the host have been intensively studied in the past years.

High-throughput short-read sequencing technologies have extensively facilitated metagenome studies to explore taxonomic and functional compositions of the chicken gut microbiota. Studies that aim to decipher the taxonomic compositions tends to sequence 16S rRNA gene amplicons (Sergeant, et al., 2014; Wen, et al., 2019), while studies that focus on both taxonomy and functions will adopt whole genome shotgun sequencing (Feng, et al., 2021). In 2018, Huang et al. firstly construct a comprehensive gene catalog of chicken gut microbiome containing ~9 million genes, through sequencing of 495 chicken samples from seven different farms in China (Huang, et al., 2018). Then, several endeavors have been made to construct the metagenome assembled genomes (MAGs) from the fragmental contigs. In 2020, Glendinning et al. constructed 469 draft MAGs using the gut metagenomes of 24 chicken samples (Glendinning, et al., 2020). In 2021, Segura-Wang et al. reconstructed 155 MAGs from metagenomes of 751 chicken samples (Segura-Wang, et al., 2021); Gilroy et al. constructed over 5,595 MAGs based on 632 chicken metagenomes (Gilroy, et al., 2021); Feng et al. assembled 12,339 MAGs by integrating 799 public chicken gut microbiome samples from ten countries (Feng, et al., 2021). These MAGs and gene catalog constructed from short-read metagenome data provide an overview of the chicken gut microbiota landscape.

Due to the technical limitation of short-read sequencing, these metagenome assembly often results in fragmental contigs with contig N50 less than 10 Kb, and a certain part of small contigs less than 500 bp are usually excluded for downstream analysis (Huang, et al., 2018). Although these short contigs can be grouped into MAGs with binning algorithms, binning introduces several types of errors, such as incompleteness and contamination (Yue, et al., 2020). Therefore, MAGs cannot be taken as microbial reference genomes. Indeed, a considerable portion of the gene structures in the non-redundant gene catalog are not complete, limiting their use for potential applications. The advent of highly accurate long-read HiFi (high-fidelity) sequencing provides a promise for resolving these problems. Recently, a sheep fecal metagenome study using ~200 G HiFi reads data by metaFlye assembled 44 circular contigs, each corresponding to a complete reference genome (Bickhart, et al., 2022; Kolmogorov, et al., 2020). Furthermore, using the same data, the software of Hifiasm-meta generated better assembly result of 279 circular complete reference genomes (Feng, et al., 2022). In this study, we used high-fidelity long reads to improve the metagenome assemblies and gene catalogs of the chicken gut microbiomes.

Results

Longer contigs of chicken metagenome assembled from high-fidelity long reads

We collected 150 digesta samples from the five intestinal compartments (duodenum, jejunum, ileum, cecum, and colorectum) of 30 chickens (Lingnan yellow broilers) slaughtered on day 42, extracted the metagenomic DNA, combined the DNA samples and constructed sequencing libraries for each intestinal compartment. Then, we generated 22 Gb, 45 Gb, 73 Gb, 81 Gb, 112 Gb PacBio HiFi reads for duodenum, jejunum, ileum, cecum, and colorectum, respectively (Table 1). For the total 332 Gb HiFi reads, the N50 read length is 17 Kb, and the median read quality value is 32, which are comparable to previous HiFi metagenome studies (Bickhart, et al., 2022; Feng, et al., 2022). The increasing amount of HiFi reads from duodenum to colorectum was associated with the microbial diversity of different intestinal compartments (Huang, et al., 2018), in order to recover more microbial species.

We assembled the HiFi reads into contigs for each intestinal compartment independently by Hifiasm-meta (Feng, et al., 2022), which results in linkage graphs of contigs. Taking the colorectum as an example, we saw a super complex, several tangled circular, hundreds of circular, and a lot of linear topologies in the contig graph (Figure 1, Figure S1). By checking the taxonomic components and reads coverage depth for each topology, we found that the super complex contains tens of various microbial genomes sharing some similar genomic fragments; the tangled circles contain many different strains of one species, and high redundancy of overlapped contigs makes the tangled circles seems much bigger than the real size of the species genome; the circular and linear contigs represent complete and incomplete genomes for a single microbial strain or species, respectively.

The total contig sizes are 0.22 Gb, 0.56 Gb, 0.85 Gb, 3.11 Gb, 3.96 Gb and contig N50 sizes are 28 Kb, 29 Kb, 34 Kb, 193 Kb and 165 Kb for duodenum, jejunum, ileum, cecum, and colorectum, respectively (Figure 2ab, Table S1). In comparison, the contig N50 size from short-read metagenome assembly is usually lower than 10 Kb (Huang, et al., 2018), suggesting that HiFi reads assembly has largely improved the contig continuity. The foregut (duodenum, jejunum, ileum)

assemblies contain more fragmental contigs than the hindgut (cecum, colorectum), which may be explained by the fact that the foregut contains only a few of dominant microbial species and other species with very low abundance. In comparison, the hindgut (cecum, colorectum) contains hundreds of abundant microbial species, and their abundance distribution are relatively more even. Though genomic complexity may also lead to fragmental contigs, we observed a non-trivial correlation between contig size and coverage depth, indicating that insufficient coverage depth of microbes with very low abundance is the primary reason for most of the fragmental contigs (Figure 2c, Figure S2).

Hundreds of complete circular genomes and binned non-circular MAGs

For duodenum, jejunum, ileum, cecum, and colorectum, we obtained 22, 25, 41, 120 and 173 reference microbial genomes of circular contigs, and recovered 5, 15, 21, 165 and 161 metagenome-assembled genomes (MAGs) from binning of non-circular contigs, resulting in total of 27, 40, 62, 285 and 334 assembled microbial genomes that passed the medium-quality criteria, respectively (Figure 3a, Table S2). Most of the circular genomes met the near-complete criteria, while the non-circular MAGs include more of those with relatively lower qualities, referred as high-quality and medium-quality. Previously, the Hifiasm-meta project has used a small portion of our data in this study for software testing, and assembled 62 circular microbial genomes that meets near-complete criteria using 33.6 G chicken cecum data (Feng, et al., 2022). In this study, using a total of 81 G cecum data, we successfully assembled 110 circular microbial genomes with near-complete quality. This result indicates that more complete genomes can be assembled by increasing the sequencing depth.

For the assembled microbial genomes within each intestinal compartment, the sequence divergences are mostly above 1%. i.e. with average nucleotide identity (ANI) below 99%, which represent for a strain-level assembly. To remove the assembly redundancy among intestinal compartments, we removed redundant genomes with the requirement of sequence divergence lower than 1% and then generated 461 non-redundant genomes of microbial strains for the chicken gut (Figure 3a). Furthermore, to remove the redundant genomes at the species-level, these 461 non-redundant microbial strain genomes were reduced to 337 non-redundant genomes with sequence divergences above 5%. Of the 461 strain-level and 337 species-level microbial genomes, 246 (53%) and 187 (55%) are circular genomes, respectively. According to the distribution analysis, the circular genomes have larger assembly sizes and higher checkM scores in comparison with the non-circular MAGs (Figure 3bc), and the assembled genome sizes have positive correlations with checkM scores (Figure S3).

Although plasmids were reported to be more difficult to assemble than host genomes in metagenomes (Pellow, et al., 2021), we were able to identify 61, 67, 71, 81 and 78 circular plasmid genomes in the Hifiasm-meta contigs for duodenum, jejunum, ileum, cecum, and colorectum, respectively (Table S3). Moreover, we also identified 33, 14, 14, 52 and 50 circular viral genomes for the corresponding intestinal compartments. The average genome size of the plasmids is 69 Kb, which is a little larger than that of the virus genomes 52 Kb. The success in assembling these circular plasmid and virus genomes is encouraging, and much more plasmid and virus fragments exist in the tangled or linear contigs, which needs further investigation.

Presence of rRNA and tRNA genes confirm the high assembly quality

In prokaryotes, the 5S, 16S, and 23S ribosomal RNA (rRNA) genes are commonly located and transcribed together, forming rRNA operons. Usually, Multiple copies of rRNA operons exist in one genome, and the repetitive characteristic makes rRNA operons difficult to assemble with short reads. Transfer RNA (tRNA) genes are randomly distributed in the genome, often with redundant copies. The identification of rRNA and tRNA genes was traditionally used as an important measurement for the completeness of genome assembly (Feng, et al., 2022). We annotated the rRNA and tRNA genes in the 461 non-redundant microbial genomes, and found that 447 (97%) genomes have at least one full-length rRNA operon coding for all three types of rRNA (5S, 16S, 23S) genes, 450 (98%) genomes have at least 18 copies of full-length tRNA genes, and 439 (95%) genomes are "RNA complete" which meets the requirements of both rRNA and tRNA criteria. Our results showed that most microbial genomes have 1-6 rRNA operons (Figure 4a), and 35-65 copies of tRNA genes (Figure 4b). In addition, the number of rRNA operon and tRNA genes in circular genomes is larger than that on non-circular MAGs (Figure 4ab), which is consistent with the completeness comparison of the microbial genomes.

Superiority of HiFi assembled genomes over short-read assembled MAGs

Numerous endeavors have been made to construct MAGs from short-read assembled contigs (Kang, et al., 2019). A recent study on chicken gut metagenome reported 12,339 dereplicated strain-level MAGs (ANI < 99%) and 1,978 dereplicated species-level MAGs (ANI < 95%), by integrating the short-read assembly of 799 public chicken gut microbiome samples from ten countries (Feng, et al., 2021). Comparing to the reported strain-level MAGs, 384 (83%) of our 461 strain-level genomes are novel (ANI < 99%), including 209 (45%) circular genomes and 175 (38%) non-circular MAGs (Figure 5a). Comparing to the reported species-level MAGs, 89 (26%) of our 337 species-level genomes are novel (ANI < 95%), including 50 (15%) circular genomes and 39 (12%) non-circular MAGs (Figure 5b). Although the currently limited sample sizes and HiFi sequencing depth have led to the smaller number of assembled microbial genomes than that of the short-read assembled MAGs (Figure 5c), HiFi assembly can recover genomes of novel species and especially novel strains, which cannot successfully be resolved by short reads assembly, due to fact that short reads cannot distinguish the highly similar sequences between microorganisms with close relationships.

The HiFi assembled microbial genomes have huge superiorities over the short-read assembled MAGs. The average contig number is 1 for our circular genomes and 2.8 for non-circular MAGs, in comparison to 257 for the short-read assembled MAGs (Figure 5d). The average assembled genome sizes are 2.61 Mb, 2.35 Mb, and 2.23 Mb, and average contig N50 sizes are 2,884 Kb, 1,697 Kb and 38 Kb for the circular genomes, non-circular MAGs, and short-read MAGs, respectively (Figure 5e-f). Moreover, the average checkM completeness percentages are 95.5, 76.4 and 89.5, and the average checkM contamination percentages are 0.85, 1.59, 2.14 for the circular genomes, non-circular MAGs, and short-read MAGs, respectively (Figure 5g-h). Almost all the evaluations of our circular genomes and non-circular MAGs are better or much better than those of the short-read assembled MAGs, except for the checkM completeness of our non-circular MAGs, which is a little lower than that of the short-read MAGs, because that the two genome datasets have used different completeness cutoffs (50% versus 80%). Overall, the HiFi assembled microbial genomes are not

only more continuous and complete than the short-read MAGs, but also have less contamination.

Advantage of HiFi-derived gene catalog over gene catalogs from short-reads

Besides MAGs, the non-redundant gene catalog was another important resource in metagenome studies. Huang et al. published the first 9.0 M gene catalog (CGM-RGC) for the chicken gut metagenome in 2018 (Huang, et al., 2018), and Feng et al. published an integrated 16.6 M gene catalog (GG-IGC) for the chicken gut metagenome in 2021 (Feng, et al., 2021). Here, we constructed a 2.5 M non-redundant gene catalog (HiFi-RGC) with the HiFi assembled contigs from all intestinal compartments. Though the gene number of our gene catalog is smaller than the two published gene catalogs due to limited sample sizes, the structure completeness ratio of our gene catalog is 99%, much higher than 38% and 63% for CGM-RGC and GG-IGC, respectively (Figure 6a-b). According to the gene sequence analysis, 847,801 (33.8%) and 724,123 (28.9%) genes are novel compared to CGM-RGC and GG-IGC (Figure c-d), suggesting that the HiFi derived gene catalog has recovered a substantial portion of the genes that are missed by short-read technologies.

Phylogeny of HiFi assembled microbial genomes and novel genomic representation

We used GTDB-tk to align the 337 HiFi assembled species-level genomes to the 47,894 species clusters (45,555 bacterial and 2,339 archaeal) in GTDB database (r202), and assign taxonomic classification to the HiFi assembled genomes based on phylogenetic placement (Chaumeil, et al., 2019). Only one genome was classified to archaea, and the other 336 genomes were all classified to bacteria. The dominant phylum is Firmicutes containing 278 (82.5%) genomes, followed by Bacteroidota and Actinobacteriota, which contain 25 (7.4%) and 14 (4.2%) genomes, respectively. In total, these three phyla covered 317 (94%) of all the assembled genomes. Noticeably, there was distinctive difference between the foregut (duodenum, jejunum, ileum) and hindgut (cecum, and colorectum) in microbial composition. The foregut was highly enriched in five genus *Ligilactobacillus*, *Limosilactobacillus*, *Lactobacillus*, *Weissella*, and *Enterococcus*, all belong to the order of Lactobacillales. In contrast, the species diversity of the hindgut was much higher, and the species was more dispersed (Figure 7, Figure S4). The difference of species composition between foregut and hindgut was consistent with previous reports from short-read metagenome studies (Huang, et al., 2018), which was caused by the difference in morphology and physiology between the foregut and hindgut. The sampling of all intestinal compartments contributes to more comprehensive microbial genome assemblies. Microbes of very low abundances in some intestinal compartment but of relatively higher abundance in other compartments could also be recovered (Figure 7).

Although all the species-level genomes have been successfully classified at the order level by GTDT-tk (Chaumeil, et al., 2019), 2, 35 and 189 genomes cannot be classified at lower taxonomic levels of family, genus, and species, respectively, suggesting that they are novel assembled genomes for these families, genera, and species (Figure 7, Figure S4). To classify these genomes at lower taxonomic ranks, we also used Ribosomal Database Project (RDP) Classifier and alignments to the Silva 16S rRNA database with the annotated 16S rRNA gene sequences from their genomes. RDP successfully classified one genome at family level, and 14 genomes at genus level, leaving 1, 21, and 189 genomes still unclassified at family, genus, and species level, respectively. Then, the alignment identities to the Silva database were used to validate the taxonomic novelty for these

genomes. We found that 58 genomes have 16S-rRNA gene identities lower than 97%, which is the threshold for demarcating bacterial species (Stackebrandt and Goebel, 1994), among which 9 genomes have 16S-rRNA gene identities lower than 95%, which is the threshold generally used to delineate a new genus (Ludwig, et al., 1998), indicating that 9 and 49 of these genomes may be corresponding to novel genus and species, respectively.

Conclusions

Given the importance of chicken production and the remarkable contribution of intestinal microbiota to the host nutrition and health, numerous endeavors have been made to construct chicken gut MAGs and gene catalogs. In the present study, we assembled 461 microbial genomes at strain level (ANI > 99%) and 337 microbial genomes at species level (ANI > 95%), of which 246 (53%) and 187 (55%) are circular genomes, respectively, using high-fidelity long reads of the five intestinal compartments of chickens. In addition, many circular plasmids and viral genomes were also successfully obtained. Among the 461 microbial genomes, 439 (95%) genomes are "RNA complete" which meet the criteria of having at least one full-length rRNA operon coding for all three types of rRNA (16S, 23S and 5S rRNA) genes and at least 18 copies of full-length tRNA genes. Besides human and sheep, chicken is now the third animal species that have comprehensive HiFi gut metagenome assemblies.

In comparison to the chicken MAGs derived from short-read metagenome assemblies, the HiFi assembled microbial genomes not only have huge advantages in continuity, completeness, and contamination metrics, but also recovered 384 (83% of 461) and 89 (26% of 337) novel strains and species, respectively. In addition, the structure completeness ratio of the 2.5 M non-redundant gene catalog constructed from HiFi assembled contigs (>99%) is much higher than that of short-read assembly derived gene catalogs (40-60%), and about one third of the genes in the HiFi derived gene catalog are novel compared to short-read derived gene catalogs. Taken together, our results showed that HiFi metagenome sequencing not only bring genomes and genes with better qualities, but also provided a substantial portion of novel genomes and genes that were missed in short-read metagenome studies.

Phylogeny analysis showed that the dominant phyla in our HiFi assembled genomes are Firmicutes (82.5%), Bacteroidota (7.4%) and Actinobacteriota (4.2%). The foregut is highly enriched in five genus *Ligilactobacillus*, *Limosilactobacillus*, *Lactobacillus*, *Weissella*, and *Enterococcus* in Lactobacillales (order), whereas the hindgut has a much wider spectrum of species. Using GTDT-tk, 2, 35 and 189 genomes failed to be classified at family, genus, and species level, suggesting that they are novel assembled genomes for these family, genus, and species, respectively. RDP Classifier further assigned one genome at family level, and 14 genomes at genus level. Among the remaining unclassified genomes, 9 and 49 genomes have 16S-rRNA gene identities lower than 95% and 97% to the Silva database, indicating that these genomes may represent for novel genus and species, respectively. The HiFi metagenome assembly not only improve the genomic representation, but also enable the discovery of novel taxonomic units. With regard to the chicken production, these novel microbial genomes or species serve as a valuable resource for functional studies, such as feed digestion, fermentation, as well as the mechanisms of disease-prevention and growth-promotion effects of antibiotics and alternatives.

Methods

Chickens raising and disease prevention

The Lingnan yellow broilers were studied for a 42-day feeding trail, with free access to feed and water. The baby chicks were bought from Zhiwei Guangdong company at day 1 age, and raised in battery cages at the farm house of Agricultural genomics institute of Shenzhen. The lighting schedule was 16 h light and 8 h dark throughout the experiment. The room temperature was controlled with heaters, gradually reduced from 35 °C on day 1 to 24 °C on day 21, and then do not change until day 42. The diets were based on the Nutrient Requirements of Poultry: Ninth Revised Edition, 1994 (NRC, 1994) and Feeding Standard of Chicken (NY/T 33-2004).

The chickens were injected Marek's Disease Vaccine and Cephalosporin on day 1, vaccinated with Newcastle disease virus (NDV, La Sota) and infectious bronchitis virus (IBV, H120) on day 7 through intranasal administration, vaccinated with NDV La Sota and IBV M41 and avian influenza H9-NJ02 on day 9 through hypodermic injection, vaccinated with Infectious Bursal Disease Virus (IBD B87) on day 14 through water drinking, vaccinated with fowlpox virus (FPV, CVCC AV1003) on day 21 through wing puncture, vaccinated with Newcastle disease virus (NDV, La Sota) on day 28 through water drinking. The Coccidiosis and other parasite diseases were also prevented by applying Diclazuril on day 17-18, Sulfaquinoxaline on day 24-25, and Albendazole on day 31-32.

Body weight records and digesta sample collections

Body weight and feed intake of the chickens were recorded for each replicate on day 42, with an average feed intake of 3.74 Kg, an average body weight of 1.99 Kg, and a feed conversion ratio of 1.93, which are consistent with the growth characteristics of this chicken breed. Then, randomly selected chickens were slaughtered on day 42 and the intestines were immediately removed and dissected. Fresh digesta samples from duodenum, jejunum, ileum, cecum, and colorectum were collected and frozen in a dry-ice pack, transported to the laboratory and stored at -80 °C until DNA extraction.

DNA extraction, library preparation, and sequencing

The digesta samples for each intestinal compartment from a total number of 30 chickens were collected for metagenomic DNA extraction. The 30 chickens were separated into 6 groups, with each group containing 5 chickens. For each group, the digesta samples for each intestinal compartment were pooled and mixed well with saline buffer containing 0.1% Tween 80 (pre-cooled at 4 °C). The microbial cells were separated through differential centrifugation to remove the undigested feed particles (Huang, et al., 2018) and the enriched microbial cells were used for DNA extraction with DNeasy PowerSoil Pro kit (47014, Qiagen) according to the manufacture's protocol. The metagenomic DNA extracted from 6 groups of the same intestinal compartment were pooled together, and further purified with VAHTS DNA Clean Beads (N411-02, Vazyme). Finally, the combined DNA samples from 30 chicken individuals for duodenum, jejunum, ileum, cecum, and colorectum were generated independently.

The high-integrity genomic DNA was fragmented into 15-20 kb inserts using g-TUBEs (Covaris,

USA), and sequencing libraries were prepared by SMRTbell Express Template Prep Kit 2.0 (PacBio, USA). Then, high-fidelity long reads were generated on PacBio Sequel II with Circular Consensus Sequence (CCS) mode (PacBio, USA). Based on the fact microbial diversity is gradually increasing from the head to end point of the intestinal tract, one, two, two, three, and three Pacbio CCS cells were used for sequencing duodenum, jejunum, ileum, cecum, and colorectum, respectively.

Metagenome contig assembly and MAG binning

To ensure assembly quality, the raw HiFi sequencing reads were filtered by requiring read length over 2 Kb and average read accuracy over 99%. In addition, the remaining reads were also mapped to the host chicken genome as well as feed genomes (maize and soybean) by minimap2 v2-2.20 (Li, 2018) with parameter "-x map-hifi", and about 2%, 0.5%, 0.5%, 0.1%, 0.1% reads were filtered as contaminations for duodenum, jejunum, ileum, cecum, and colorectum, respectively. Hifiasm-meta r058 (Feng, et al., 2022) was used to assemble the pre-filtered HiFi reads into contigs. By exploiting the contig linkages from the resulting GFA files with Bandage v0.8.1 (Wick, et al., 2015), the Hifiasm-meta contigs were divided into 3 classes: (1) circular contig, complete genome assembly of a given species; (2) tangled "circular", many fragmental contigs linked into a tangled "circular", formed by various heterozygous strains of a species; (3) linear contig, incomplete genome assembly of a species, often due to low coverage depth. Then, the circular contigs were left alone, and each tangled "circular" was re-assembled by Hifiasm-meta independently, using these fragmental contigs as input reads. Furthermore, the linear contigs were grouped into metagenome assembled genomes (MAGs) by a binning algorithm MetaBAT2 v2.12.1 (Kang, et al., 2019), with the contig depth obtained from Hifiasm-meta GFA files. CheckM (lineage_wf) v1.1.3 (Parks, et al., 2015) was utilized to evaluate the assembly quality, and 3 quality ranks were adopted: near complete (≥ 90% completeness and < 5% contamination), high quality (≥ 70% completeness and < 10% contamination), and medium quality (≥ 50% completeness and < 10% contamination).

Construction of non-redundant microbial genome assemblies

Limited by computer memory, the sequencing data of each intestinal compartment (duodenum, jejunum, ileum, cecum, and colorectum) was assembled independently. Then, the microbial genome assemblies (near-complete, high-quality, medium-quality) from all intestinal compartments were put together, and pairwise identity (0 – 100) were calculated by FastANI v1.32 (https://github.com/ParBLiSS/FastANI). The identity values were converted into distance values (100 - identity), and hierarchical clustering algorithm with maximum distance were applied. The stop distance for hierarchical clustering was set to 1 and 5 to get strain-level and species-level clusters, respectively. Then, in each cluster, circular genome was preferred than non-circular MAG, in addition, genome assembly with larger checkM score (completeness – 5 * contamination) was preferred. After taking the best genome assembly as the representative, the other genome assemblies were taken as redundance and removed. Finally, non-redundant set of microbial genomes at strain-level (ANI 99%) and species-level (ANI 95%) were generated, respectively.

Taxonomy classification and genome annotation

GTDB-Tk (classify_wf) v1.5.1 (Chaumeil, et al., 2019) and its database version r202 was used for phylogenetic placement and classification of the assembled microbial genomes, and GraPhlAn v1.1.3 (Asnicar, et al., 2015) was used for tree visualization. Ribosomal Database Project (RDP)

Classifier V2.11 (Wang, et al., 2007) was used to classify the genome lower taxonomic ranks with 16S rRNA gene sequences. BLAST V2.3.1 alignments to the Silva database (r138) (Quast, et al., 2013) were further used to validate the novelty of taxonomic units. ViralVerify v 1.1 (https://github.com/ablab/viralVerify) with parameter "--hmm nbc_hmms.hmm" was adopted to classify the assembled genomes into bacteria / archaea, plasmid and viral genomes. RNAmmer v1.2 (Lagesen, et al., 2007) with parameters "-S arc/bac -m lsu,ssu,tsu" was adopted to annotate the 5S, 16S, and 23S rRNA genes, tRNAscan-SE v2.0.3 (Chan, et al., 2021) with parameters "-G -H" was adopted to predict tRNA genes, and Prodigal (v2.6.3) (Hyatt, et al., 2010) with parameter "-p single" was used to predict protein-coding genes on the assembled microbial genomes.

Non-redundant gene catalog construction

Protein-coding gene prediction was performed on the contigs of each intestinal compartment by Prodigal (v2.6.3) (Hyatt, et al., 2010) with parameter "-p meta". Then, in order to get a non-redundant chicken gut gene catalog at species-level, the gene models from all the intestinal compartments were put together and redundance were removed by the criteria of identity > 95% and overlap > 90% of the shorter genes, using cd-hit-est v4.6.6 (Li and Godzik, 2006) with parameter "-c 0.95 -n 10 -G 0 -aS 0.9".

Acknowledgements

We thank Dan Liu and Wenshu Liu for giving suggestions on the selection of chicken breed. We thank Yazhi Qin for helping raising and slaughtering of the chickens. The work was funded by National Natural Science Foundation of China (Grant No. 32000408), the Agricultural Science and Technology Innovation Program of CAAS, and fund of Key Laboratory of Shenzhen (ZDSYS20141118170111640).

Reference

Asnicar, F., *et al.* Compact graphical representation of phylogenetic data and metadata with GraPhlAn. *PeerJ* 2015;3:e1029.

Bickhart, D.M., *et al.* Generating lineage-resolved, complete metagenome-assembled genomes from complex microbial communities. *Nat Biotechnol* 2022;40(5):711-719.

Chan, P.P., *et al.* tRNAscan-SE 2.0: improved detection and functional classification of transfer RNA genes. *Nucleic Acids Res* 2021;49(16):9077-9096.

Chaumeil, P.A., *et al.* GTDB-Tk: a toolkit to classify genomes with the Genome Taxonomy Database. *Bioinformatics* 2019.

Feng, X., *et al.* Metagenome assembly of high-fidelity long reads with hifiasm-meta. *Nat Methods* 2022.

Feng, Y., *et al.* Metagenome-assembled genomes and gene catalog from the chicken gut microbiome aid in deciphering antibiotic resistomes. *Commun Biol* 2021;4(1):1305.

Gilroy, R., *et al.* Extensive microbial diversity within the chicken gut microbiome revealed by metagenomics and culture. *PeerJ* 2021;9:e10941.

Glendinning, L., *et al.* Assembly of hundreds of novel bacterial genomes from the chicken caecum. *Genome Biol* 2020;21(1):34.

Huang, P., *et al.* The chicken gut metagenome and the modulatory effects of plant-derived benzylisoquinoline alkaloids. *Microbiome* 2018;6(1):211.

Hyatt, D., *et al.* Prodigal: prokaryotic gene recognition and translation initiation site identification. *BMC Bioinformatics* 2010;11:119.

International Chicken Genome Sequencing, C. Sequence and comparative analysis of the chicken genome provide unique perspectives on vertebrate evolution. *Nature* 2004;432(7018):695-716.

Kang, D.D., *et al.* MetaBAT 2: an adaptive binning algorithm for robust and efficient genome reconstruction from metagenome assemblies. *PeerJ* 2019;7:e7359.

Kolmogorov, M., *et al.* metaFlye: scalable long-read metagenome assembly using repeat graphs. *Nat Methods* 2020;17(11):1103-1110.

Lagesen, K., *et al.* RNAmmer: consistent and rapid annotation of ribosomal RNA genes. *Nucleic Acids Res* 2007;35(9):3100-3108.

Li, H. Minimap2: pairwise alignment for nucleotide sequences. *Bioinformatics*

2018;34(18):3094-3100.

Li, W. and Godzik, A. Cd-hit: a fast program for clustering and comparing large sets of protein or nucleotide sequences. *Bioinformatics* 2006;22(13):1658-1659.

Ludwig, W., *et al.* Bacterial phylogeny based on comparative sequence analysis. *Electrophoresis* 1998;19(4):554-568.

Oakley, B.B., *et al.* The chicken gastrointestinal microbiome. *FEMS Microbiol Lett* 2014;360(2):100-112.

Parks, D.H., *et al.* CheckM: assessing the quality of microbial genomes recovered from isolates, single cells, and metagenomes. *Genome Res* 2015;25(7):1043-1055.

Pellow, D., *et al.* SCAPP: an algorithm for improved plasmid assembly in metagenomes. *Microbiome* 2021;9(1):144.

Quast, C., *et al.* The SILVA ribosomal RNA gene database project: improved data processing and web-based tools. *Nucleic Acids Res* 2013;41(Database issue):D590-596.

Rubin, C.J., *et al.* Whole-genome resequencing reveals loci under selection during chicken domestication. *Nature* 2010;464(7288):587-591.

Segura-Wang, M., *et al.* Genome-Resolved Metagenomics of the Chicken Gut Microbiome. *Front Microbiol* 2021;12:726923.

Sergeant, M.J., *et al.* Extensive microbial and functional diversity within the chicken cecal microbiome. *PLoS One* 2014;9(3):e91941-e91941.

Stackebrandt, E. and Goebel, B.M. Taxonomic note: A place for DNA:DNA reassociation and 16s rRNA sequence analysis in the present spec. In.; 1994.

Wang, Q., *et al.* Naive Bayesian classifier for rapid assignment of rRNA sequences into the

new bacterial taxonomy. *Appl Environ Microbiol* 2007;73(16):5261-5267.

Wen, C., *et al.* The gut microbiota is largely independent of host genetics in regulating fat deposition in chickens. *The ISME journal* 2019;13(6):1422-1436.

Wick, R.R., *et al.* Bandage: interactive visualization of de novo genome assemblies. *Bioinformatics* 2015;31(20):3350-3352.

Wong, G.K., *et al.* A genetic variation map for chicken with 2.8 million single-nucleotide polymorphisms. *Nature* 2004;432(7018):717-722.

Yeoman, C.J., *et al.* The microbiome of the chicken gastrointestinal tract. *Anim Health Res Rev* 2012;13(1):89-99.

Yue, Y., *et al.* Evaluating metagenomics tools for genome binning with real metagenomic datasets and CAMI datasets. *BMC Bioinformatics* 2020;21(1):334.

Data availability

The HiFi sequencing reads can be found under BioProject ID PRJNA748109: SRR19683891 for duodenum, SRR19732514 and SRR19726169 for jejunum, SRR19736685 for ileum, SRR19732730 for cecum, and SRR19683890 and SRR19732729 for colorectum. The assembled contigs, microbial genomes for each intestinal compartments and non-redundant sets at species and strain levels, non-redundant gene catalog, as well as plasmid and viral annotations are available at AGIS website (ftp://ftp.agis.org.cn/~fanwei/Chicken_gut_metagenome_Hifi/).

Author contributions

Y.Z. and W.F. designed and coordinated the research. Y.Z. and B.Y. prepared chicken gut materials for sequencing. F.J. and B.Y. performed data analysis. W.F. wrote the manuscript, and all authors provided suggestions and revised the manuscript.

Ethics approval

This study was approved by the Life Science Ethics Committee of Agricultural Genomics Institute, Chinese Academy of Agricultural Sciences.

Competing interests

The authors declare no competing interests.

Tables

Table 1. Statistics of Pacbio HiFi sequencing data

Intestinal compartment	Pabio Cell number	Number of reads	Number of bases (bp)	N50 read length (bp)	Median read quality (Phred)
Duodenum	1	2,734,871	22,233,516,165	9,778	39
Jejunum	2	2,669,321	44,559,115,216	16,417	35
Ileum	2	4,282,202	72,828,594,344	16,856	33
Cecum	3	5,045,925	80,959,163,166	17,319	31
Colorectum	3	5,865,946	111,891,321,947	19,258	31
All	11	20,598,265	332,471,710,838	17,316	32

Figures

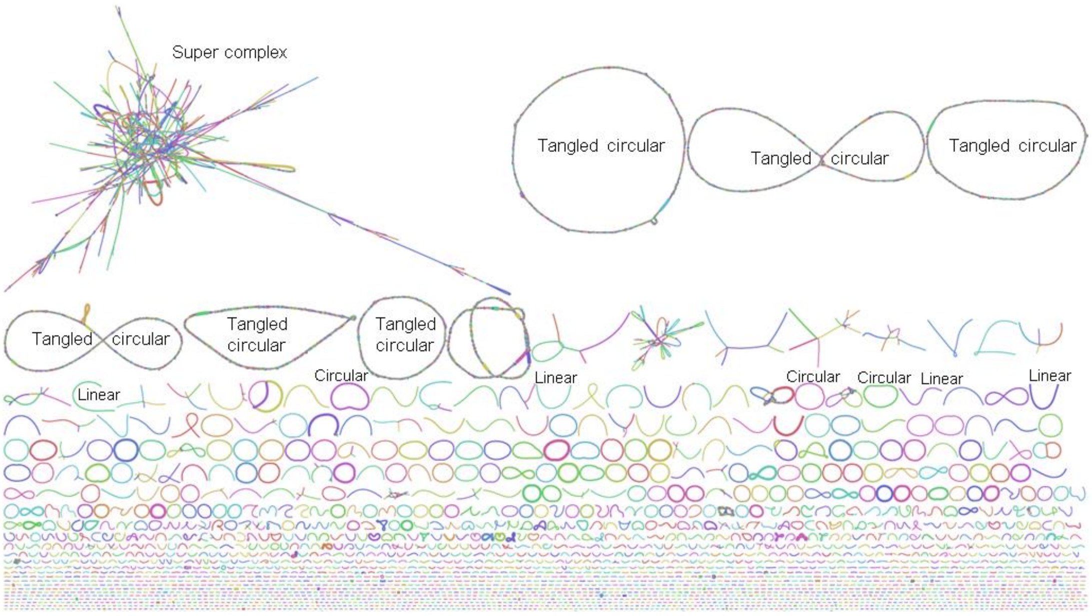

Figure 1. Graphic display of the contig assembly graph. Random colors were chosen for different contigs. The line length is in proportion to contig length, and the line width is in proportion to contig coverage depth. Some examples for super complex, tangled circular, individual circular and linear contigs were labeled. This plot is for the colorectum assembly drawn by Bandage, and those for the other intestinal compartments are shown in Figure S1.

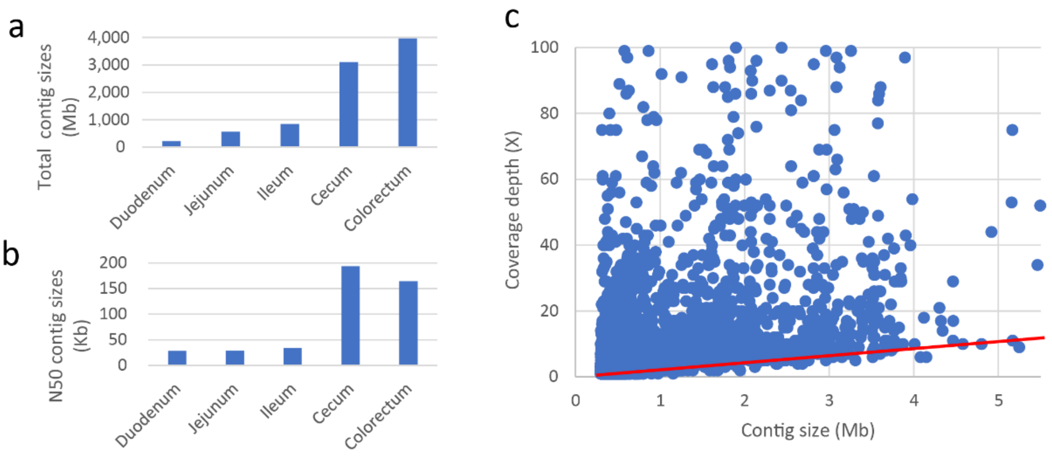

Figure 2. Statistics of contig assembly. (a) Histogram of total assembled contig sizes for each intestinal compartment. (b) Histogram of N50 contig sizes for each intestinal compartment. (c) Correlation plot of contig length and coverage depth, using data from all intestinal compartments. The red marker line indicates that sufficient coverage depth contributes to the contig continuity.

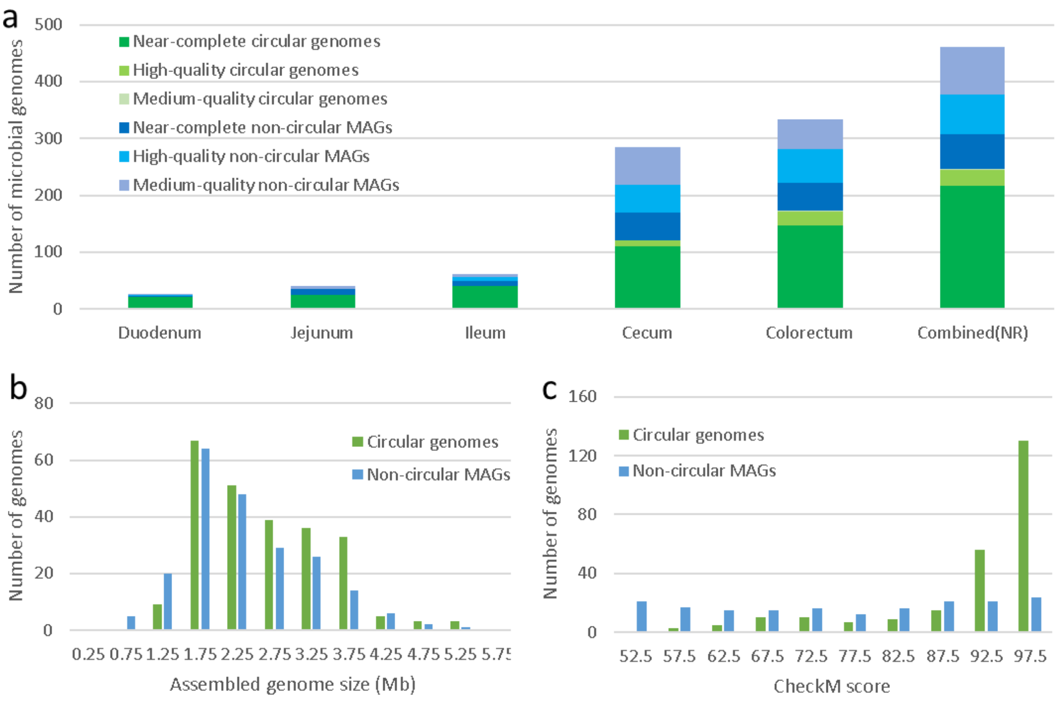

Figure 3. Evaluation and ranking of assembled microbial genomes. (a) Circular genomes refer to circular contigs, and non-circular MAGs refer to incomplete genome assemblies derived from contig binning or merging algorithms. A circular genome or non-circular MAG is defined as 'near-complete', if its CheckM completeness is ≥90% and its contamination level ≤5%, defined as 'high-quality' if completeness ≥70% and contamination ≤10%, or defined as 'medium-quality' if completeness ≥50% and contamination ≤10%. Combined (NR) is the non-redundant set of microbial genomes from all intestinal compartments. All the microbial genomes in Combined (NR) have ≤ 99% ANI to the other microbial genomes in Combined (NR). (b) Distribution of the assembled microbial genome sizes for circular genomes and non-circular MAGs. (c) Distribution of the checkM scores (completeness – 5 * contamination) for circular genomes and non-circular MAGs.

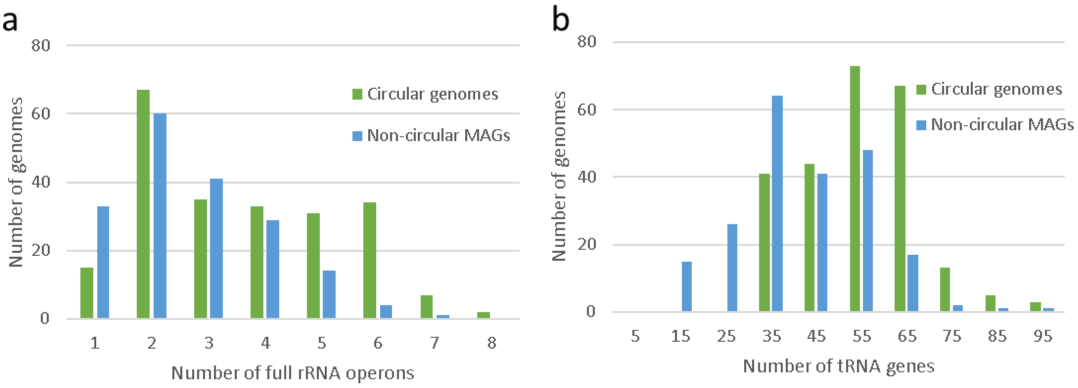

Figure 4. Statistics of non-coding RNA genes in assembled microbial genomes. (a) Distribution of the number of full rRNA operon, which codes for a 5S, a 16S, and a 23S rRNA. (b) Distribution of the number of tRNA genes.

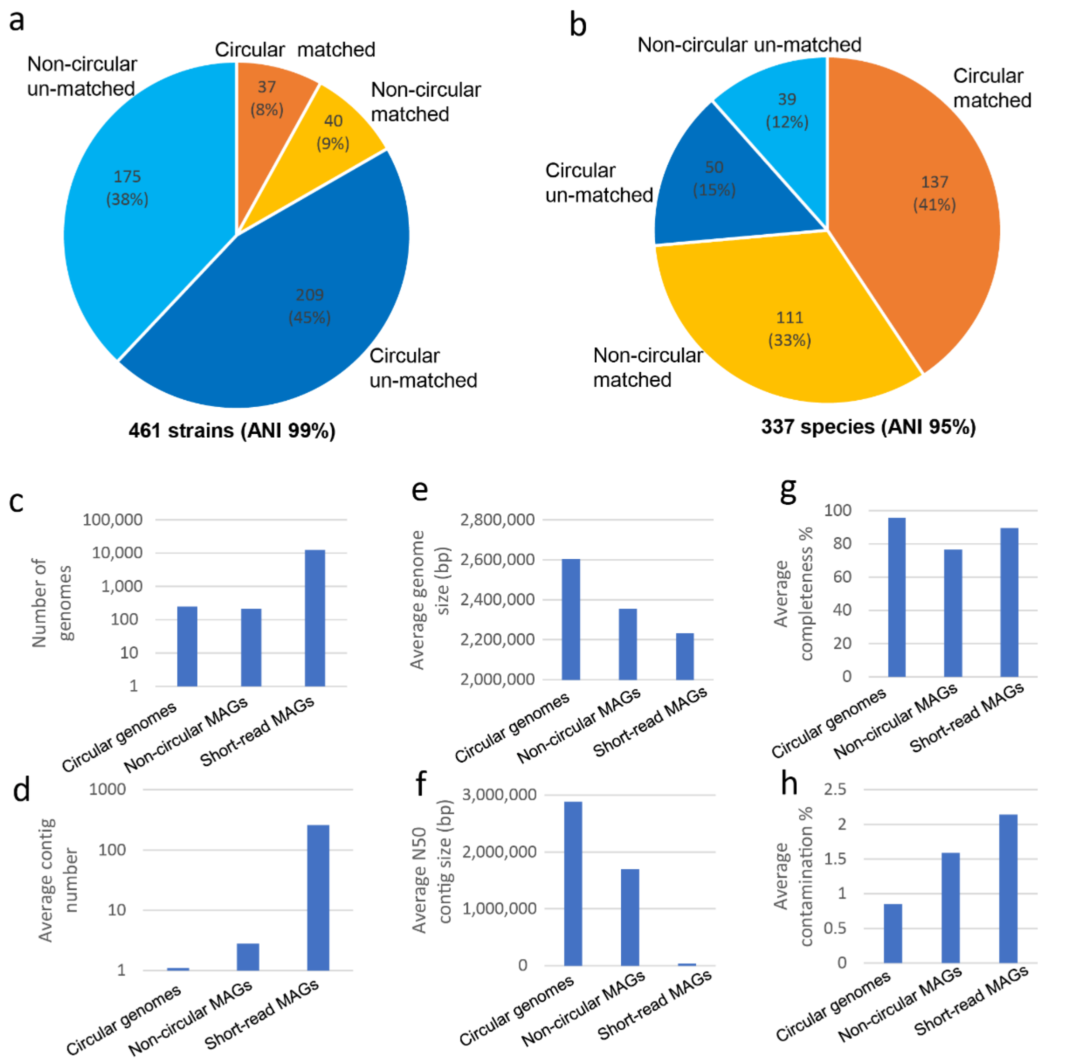

Figure 5. Comparison of HiFi assembled microbial genomes with short-read assembled MAGs. (a) Matching of our 461 assembled microbial strain-level genomes (99% average nucleotide identity, ANI) with public 12,339 dereplicated MAGs (99% ANI) derived from short-reads. Criterion for matching: HiFi assembled microbial genome has ANI higher than 99% to any short-read assembled MAG. (b) Matching of our 337 assembled microbial species-level genomes (95% ANI) with public 1,978 dereplicated MAGs (95% ANI) derived from short-reads. Criterion for matching: HiFi assembled microbial genome has ANI higher than 95% to any short-read assembled MAG. The un-matched microbial genomes unveil candidates of novel strains and species. (c) Number of genomes, (d) average contig number, (e) averaged assembled genome size, (f) average N50 contig size, (g) average checkM completeness, (h) average checkM contamination of the circular genomes, non-circular MAGs and public chicken gut MAGs assembled from short-reads.

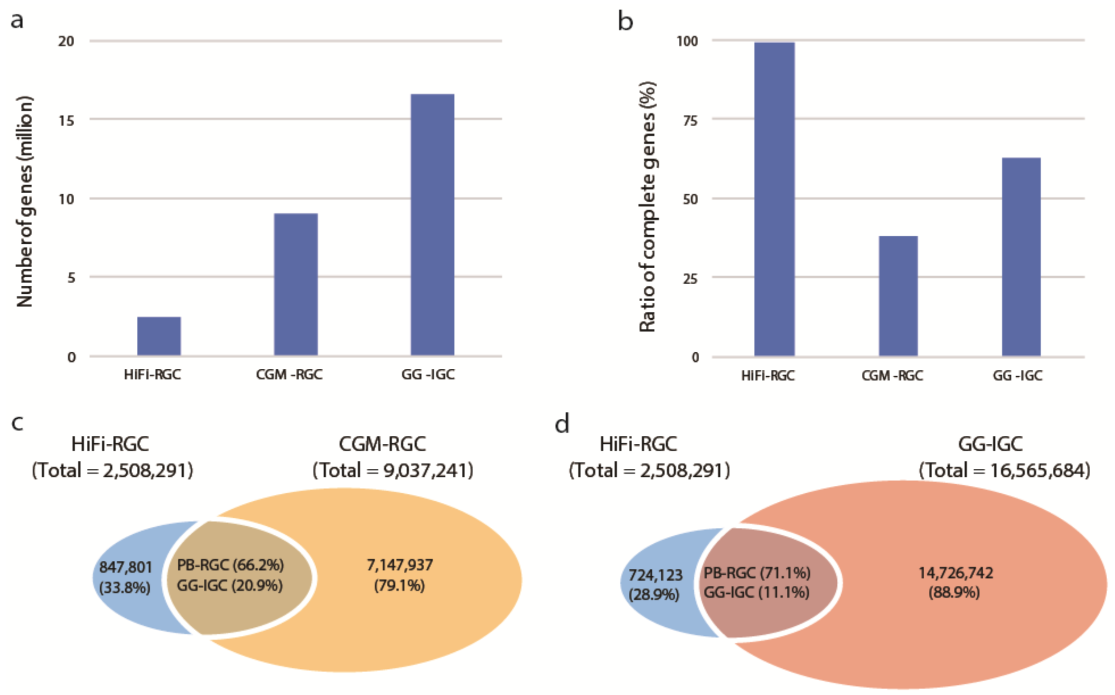

Figure 6. Comparison of HiFi-derived reference gene catalog (HiFi-RGC) with two published gene catalogs derived from short-read sequencing data (CGM-RGC and GG-IGC). CGM-RGC refer to chicken gut metagenome – reference gene catalog published by Huang et al. in 2018, GG-IGC refer to Gallus gallus – Integrated gene catalog published by Feng et al. in 2021. (a) Gene number and (b) gene structure completeness ratio of the 3 gene catalogs. Overlap of HiFi-RGC and CGM-RGC (c) and GG-IGC (d). A confident share is defined by the criteria of sequence identity ≥95% and length overlap ≥90% of the shorter sequence.

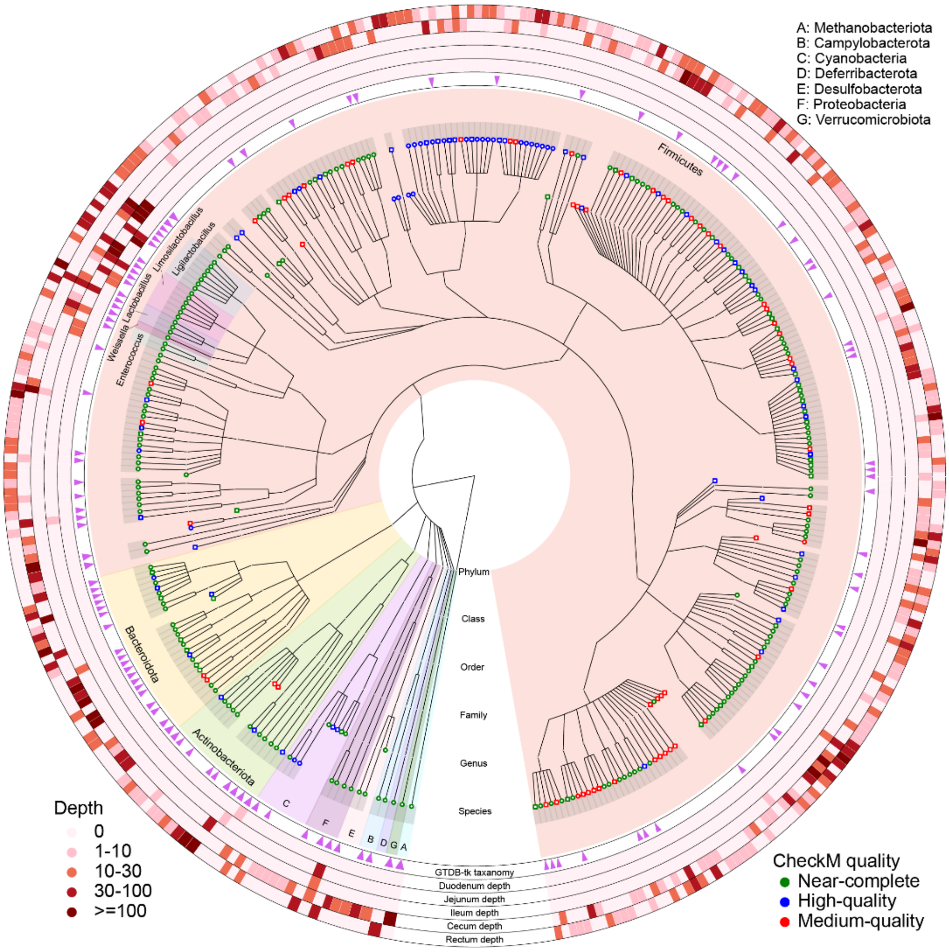

Figure 7. Phylogeny of the HiFi-assembled microbial genomes. A colored clade corresponds to a phylum inferred by GTDB-Tk. Inside the largest phylum Firmicutes, five genus *Ligilactobacillus*, *Limosilactobacillus*, *Lactobacillus*, *Weissella*, and *Enterococcus* are also colored for highlighting. The leaf nodes of the phylogenic tree have two shapes: "circle" represents circular genome, "rectangle" represents non-circular MAG. The colors of the leaf nodes represent checkM quality ranks: "green" refers to near-complete, "blue" refers to "High-quality", and "red" refers to "Medium-quality". The inner ring shows GTDB-tk classification, and a triangle means the corresponding leaf node is matched to an existing genome in the GTDB database. The five outer rings show the sequencing coverage depth for each assembled microbial genome from each intestinal compartment, respectively.

Supplementary figures and tables

Click here to access/download
Supplementary Material
Supplemental-20220703-final.docx
