# Supplementary material for: Improved microbial genomes and gene catalog of the chicken gut from metagenomic sequencing of high-fidelity long reads
Source: Gigascience. 2022 Nov 18;11:giac116. doi: 10.1093/gigascience/giac116 (PMC9673493; doi:10.1093/gigascience/giac116)
Supplement: giac116_GIGA-D-22-00175_Revision_1 [file giac116_giga-d-22-00175_revision_1.pdf]

GigaScience

Improved microbial genomes and gene catalog of chicken gut from metagenomic sequencing of high-fidelity long reads

--Manuscript Draft--

Manuscript Number:	GIGA-D-22-00175R1
Full Title:	Improved microbial genomes and gene catalog of chicken gut from metagenomic sequencing of high-fidelity long reads
Article Type:	Research
Funding Information:	National Natural Science Foundation of China (Grant No. 32000408) — Dr. Yan Zhang
Abstract:	Background<br><br>Due to the importance of chicken production and the remarkable influence of gut microbiota on the host's health and growth, tens of thousands of metagenome-assembled genomes (MAGs) have been constructed for the chicken gut microbiome. However, limited by the short-read sequencing and assembly technologies, most of these MAGs are far from complete and have lower qualities with contamination.<br><br>Results<br><br>We generated 332 Gb high-fidelity (HiFi) long reads from the five chicken intestinal compartments, and assembled 461 and 337 microbial genomes at species and strain level, of which 53% and 55% are circular genomes, respectively. For the assembled microbial genomes, about 95% were regarded as complete according to the "RNA complete" criteria, which requires at least one full-length rRNA operon coding for all three types of rRNA(16S, 23S and 5S rRNA) and at least 18 copies of full-length tRNA genes. In comparison with the short-read derived chicken MAGs, 384 (83% of 461) and 89 (26% of 337) strain-level and species-level genomes in this study are novel and do not have any matches. At the gene level, a third of the 2.5 million genes in HiFi derived gene catalog are novel and cannot be matched to the short-read derived gene catalogs. Moreover, the HiFi derived genomes have much higher continuity and completeness, as well as lower contamination; the HiFi derived gene catalog has much higher ratio of complete gene structures. The dominant phyla in our HiFi assembled genomes is Firmicutes (82.5%), and the foregut is highly enriched in five genera Ligilactobacillus , Limosilactobacillus , Lactobacillus , Weissella , and Enterococcus , all of which belongs to the order Lactobacillales. Using GTDB-tk, all the 337 species-level genomes were successfully classified to order level, however, 2, 35 and 189 genomes could not be classified into any known family, genus, and species, respectively. Among these not-fully classified genomes, 9 and 49 of them may belong to novel genera and species, respectively, for that their 16S-rRNA genes have identities lower than 95% and 97% to any known 16S-rRNA genes.<br><br>Conclusions<br><br>HiFi sequencing not only remarkably improved the quality of metagenome assemblies and gene structures, but also recovered a substantial portion of novel genomes and genes that were missed in short-read metagenome studies. The novel genomes or novel species obtained in this study will facilitate the gut microbiome and host-microbiota interaction studies, which would benefit the sustainable development of poultry.
Corresponding Author:	Wei Fan<br>Chinese Academy of Agricultural Sciences<br>shenzhen, guangdong CHINA
Corresponding Author Secondary Information:	
Corresponding Author's Institution:	Chinese Academy of Agricultural Sciences

Powered by Editorial Manager® and ProduXion Manager® from Aries Systems Corporation

**Corresponding Author's Secondary Institution:**	
**First Author:**	Wei Fan
**First Author Secondary Information:**	
**Order of Authors:**	Wei Fan
	Yan Zhang
	Fan Jiang
	Boyuan Yang
	Sen Wang
	Hengchao Wang
	Anqi Wang
	Dong Xu
**Order of Authors Secondary Information:**	
**Response to Reviewers:**	Editor comments:<br><br>In addition, please register any new software application in the bio.tools and SciCrunch.org databases to receive RRID (Research Resource Identification Initiative ID) and biotoolsID identifiers, and include these in your manuscript. This will facilitate tracking, reproducibility and re-use of your tool.<br><br>Reply: we have registered new software in the SciCrunch.org databases, and included RRIDs of all the used software in our manuscript.<br><br><br>Reviewer reports:<br><br>Reviewer #1: The authors sequenced the chicken gut microbiome using HiFi sequencing based on PacBio Sequel II platform, and further revealed its advantages compared with traditional short-read sequencing: higher quality of assemblies for gene structures and more recovered novel genes and genomes. Although the conclusions obtained in this study are right, this work meets our expectations and is a commonsense. In addition, this study is a little bit simple because they only compare the two sequencing technologies. The authors should perform some analysis about scientific findings in chicken gut microbiome. The detailed comments are as following：<br><br>1. This paper has spent large effort to prove the advantage of the high-fidelity (HiFi) sequencing, but a lot of previous research have reported the advantage of the high-fidelity (HiFi) sequencing and it is a commonsense. What is really new in this paper? The authors should reveal the scientific findings among different chicken-gut microbiota.<br><br>Reply: Thanks to the reviewer’s suggestions. As this is the first HiFi metagenome project for chicken, we have performed detailed comparisons of the HiFi metagenome data and short-read metagenome data. Although the advantage of HiFi metagenome sequencing has been shown by a few previous studies, our analysis has used different assembly method, so we think it is necessary to make detailed comparisons again, which can assure that the chicken HiFi-derived microbial genomes and gene catalogue are both in high quality.<br><br>We admit that the experiment design of this study is a bit simple. However, we think that the quick release of the generated resources, including the assembled novel strain and species genomes, as well as the predicted novel genes, will be very helpful to the scientific world and chicken production industry. We have also made several scientific findings from our metagenome data analysis, such as: (1) The dominant phyla in our HiFi assembled genomes is Firmicutes (82.5%), and the foregut is highly enriched in five genera Ligilactobacillus, Limosilactobacillus, Lactobacillus, Weissella, and Enterococcus. (2) Among the 337 species-level microbial genomes, 9 and 49 of them

Powered by Editorial Manager® and ProduXion Manager® from Aries Systems Corporation

may belong to novel genera and species, respectively.

Now we have added some more analysis and descriptions in the manuscript, and the original Result section “Phylogeny of HiFi assembled microbial genomes and novel genomic representation”was split into two sections “Phylogeny of HiFi assembled microbial genomes and differences among intestinal compartments” and “Novel genomic representation and novel genus and species discovery”. Some important revisions were listed below:

(1)In Result section “Phylogeny of HiFi assembled microbial genomes and differences among intestinal compartments”, we have added:

“The remaining genomes were classified as Cyanobacteria, Proteobacteria, Desulfobacterota, Campylobacterota, Deferribacterota, Methanobacteriota, and Verrucomicrobiota.”;

“The foregut contains the duodenum, jejunum, and ileum, which mainly function in feed digestion and nutrient absorption. The hindgut contains the cecum and colorectum, which function in fermentation, detoxification and recycling of residual water and salt.”;

(2) In Result section “Novel genomic representation and novel genus and species discovery”, we have added:

“Some of these new genomic sequences may have potential benefits to industry or medical applications. Lactobacillus has traditionally been used in the fermentation industry, producing lactate from raw carbohydrates and synthetic media (Sun, et al., 2015). In recent years, Lactobacillus and its close relatives Ligilactobacillus and Limosilactobacillus have also been widely adopted as probiotic supplements, either in animal feed to promote growth or human foods to improve human health (Sarao and Arora, 2017). Among our 337 assembled microbial species genomes, 3 genomes belong to Lactobacillus, 7 genomes belong to Ligilactobacillus, and 6 genomes belong to Limosilactobacillus. All these genomes have been successfully classified to the genus level, and most of the genomes were successfully classified to the species level by GTDB-Tk. However, 2 Ligilactobacillus genomes and 1 Limosilactobacillus genome have not been classified to the species level, suggesting that these 3 species-level genomes may represent novel genomic resources for probiotic development.”;

“In addition, approximately one-third of these newly discovered genera and half of these newly discovered species were not found in the short-read MAG data, suggesting that they are derived only from HiFi metagenome data, which further shows the advantage of HiFi sequencing in metagenomic studies.”;

(3) In Result section “Advantage of HiFi-derived gene catalog over gene catalogs from short-reads”, we have added a paragraph:

“By comparing the pairwise overlap at the gene sequence level, we found that 847,801 (33.8%) and 724,123 (28.9%) genes are unique in HiFi-RGC compared to CGM-RGC and GG-IGC, respectively (Figure 6c-d), suggesting that the HiFi-derived gene catalog recovered a substantial portion of the genes that were missed by short-read technologies. Because GG-IGC is more comprehensive than CGM-RGC, we considered the 724,123 (28.9%) genes in HiFi-RGC as unique genes and the remaining genes (71.1%) in HiFi-RGC as shared genes. Then, the microbial communities derived from the unique and shared genes in HiFi-RGC were compared. The results showed that 36.8% of unique genes were unclassified at the phylum level, which was obviously higher than the proportion of shared genes (24.9%), suggesting that the unique genes are enriched in unknown phyla (Figure S6).”

2. Method section: "The 30 chickens were separated into 6 groups, with each group containing 5 chickens.". However, the digesta samples of the 30 chickens were finally pooled, mixed together and analyzed based on the difference of intestinal compartment. Pls elucidate what the meaning of classification is?

Reply: The mentioned “classification” was just for the convenience of experimental

processing, as the total volume of digesta from all chickens was large and difficult to process in one time. For each intestinal compartment，samples from all the 30 chickens were pooled altogether in two steps of pooling (digesta pooling and extracted DNA pooling). The manuscript has been revised with more detailed and clear description：

“Mainly due to the volume of digesta, it was difficult to process all of the samples at one time. For the convenience of processing, the duodenum digesta from every 5 chickens were pooled together and then washed for microbial cell enrichment and DNA extraction. After processing all duodenum samples, the metagenomic DNA was finally pooled, and further purified with VAHTS DNA Clean Beads (N411-02, Vazyme). The metagenomic DNA samples of the jejunum, ileum, cecum and colorectum were processed in the same way, except that for the cecum, due to its relatively high microbial density, only a subfraction of the pooled and thoroughly mixed digesta was used for microbial cell enrichment and DNA extraction.”

3. The line number should be added in the manuscript, and English needs to be polished throughout the MS.

Reply: We have added the line number and polished the English by a native speaker.

Reviewer #2: The authors present work that expands the databases of chicken microbiome genomes with high quality assemblies and represents a valuable resource. The authors also demonstrate a step towards moving from short read assembly of metagenomes towards long read assemblies, which is undoubtedly the direction the field should be moving in.

While I expect these genomes to be high quality and not heavily impacted by indels given the accuracy of hifi and the high completeness statistics, a common criticism of long-reads is the error rate. Have the authors considered demonstrating that this is not impacting their high quality genomes through, for example, the ideel tool or some other assessment of error rate in the assemblies? This may strengthen the message that long reads are suitable for this kind of work, particularly if the result is independent of the depth of coverage over each species.

Reply: Before metagenome assembly, we have pre-filtered low quality HiFi reads, and only HiFi reads with quality over Q20 were used for metagenome assembly. The high base quality of input reads makes sure that the high base quality of the contig assembly.

In respect to the hifiasm-meta algorithm, it first performs HiFi-reads error correction from overlapped reads, which is equivalent to call the consensus sequences from multiple aligned reads. So, higher reads coverage will improve the error correction of the HiFi reads, and finally improve the base accuracy of the contig assemblies. In the hifiasm-meta paper (Feng, X., et al. Metagenome assembly of high-fidelity long reads with hifiasm-meta. Nat Methods 2022), Extended Data Fig.2 shows Yak quality value (QV) score correlated with contig coverage, and higher coverage will result in higher Yak QV. Yak QV is a rough estimation of the single base accuracy, including both mismatch and indel errors.

Here, we performed the same analysis using our chicken metagenome contig data, and obtained a similar result, which was shown in Figure 2d: Correlation plot of YAK quality score (QV) and coverage depth, using contigs with length over 100 Kb from all intestinal compartments. The K-mer frequency was calculated with parameters “yak count -b37 -t48” and yak QV was calculated with parameters “yak qv -t80 -p -K3.2g -l100k”. The red marker line indicates that higher coverage depth will improve the single base quality of the contig sequences.

We have also added descriptions in the maintext (Results/Longer contigs of the

Powered by Editorial Manager® and ProduXion Manager® from Aries Systems Corporation

chicken metagenome assembled from high-fidelity long reads): “Moreover, the coverage depth is positively related with the single-base quality values, indicating that higher coverage depth will improve the single-base accuracy of the contig sequences (Figure 2d).”.

In addition, we also used the checkM completeness to evaluate the single base accuracy of the assembled genomes: We added a supplementary figure S5: Correlation plot of genome coverage depth and checkM completeness score. The 187 circular genomes out of 337 non-redundant species-level genomes were used here. Considering all these genomes have complete genome assemblies, the difference of checkM completeness scores should only be caused by the single base accuracy, due to the marker gene prediction method adopted by checkM. Genome assemblies with higher single base accuracy will have higher checkM completeness values.The plots clearly shows that higher coverage depth will result in higher checkM completeness scores, indicating that higher coverage depth will improve the single base accuracy of genome assemblies.

We also added descriptions in the maintext “Using the 187 circular species-level genomes, which all have complete genome assemblies, we showed that higher coverage depth is positively correlated with CheckM completeness score, indicating that a higher coverage depth will improve the single-base accuracy of the genome assemblies (Figure S5).”

Can the authors elaborate on the methods they used to compare the existing gene catalogs to their own as this appears to not be described in the methods section?

Reply: We have added the methods for comparing the gene catalogs in Methods/ Non-redundant gene catalog construction:

To compare the overlap of our gene catalog (HiFi-RGC) with two published chicken gut metagenome gene catalogs (CGM-RGC and GG-IGC) (Feng, et al., 2021; Huang, et al., 2018), pairwise alignments of HiFi-RGC to CGM-RGC and HiFi-RGC to GG-IGC were performed using BLAT (BLAT, RRID:SCR_011919) (Kent, 2002) with identity ≥ 95% and overlap ≥ 90% of the shorter genes as the criteria for shared genes.

Can the authors include in the supplement some stats on the quantity and quality of DNA extracted from each sample type? It is very useful for people considering using the same protocol especially when long-read sequencing typically requires high levels of high quality starting material.

Reply: We have added the quantity and quality information in Table S1 and Figure S1. The DNA quality and quantity were measured by Invitrogen Qubit 4 Fluorometer with Qubit™ dsDNA BR(Invitrogen, Q32850) and by Nanodrop 2000c Microvolume Spectrophotometer (Table S1). The integrity of DNA was evaluated on field electrophoresis agarose gels (Figure S1).

Table S1. Quality and Quantity assessment of the extracted DNA

Figure S1. Agarose gel electrophoresis. (a) DC electrophoresis (0.7% gel, 100V, 1h) for Duodenum microbiota DNA (3011A); M1 15kb DNA Marker(15000、10000、7500、5000、2500、1000、250bp); M2 λDNA /HindIII(23130、9416、6557、4361、2322、2027、564bp). (b) Pulse electrophoresis (0.7% gel, pulse 5~80kb, 16h) for Duodenum microbiota DNA (3011A); M1 15kb DNA Marker; M2 λDNA /HindIII; (c) DC electrophoresis (1% gel, 180V, 20min) for Jejunum microbiota DNA (lane 1), Ileum microbiota DNA (lane 2), Cecum microbiota DNA (lane 3), and Colorectum microbiota DNA (lane 4); S stantard sample (50ng); M-1 trans 2k plus; M-2 trans 15k plus. (d) Pulse electrophoresis (0.8% gel, pulse 5~80kb, 17h) for Jejunum microbiota DNA (lane 1), Ileum microbiota DNA (lane 2), Cecum microbiota DNA (lane 3), and Colorectum microbiota DNA (lane 4); M 48kb DNA Extension Ladder. In summary, the microbiota DNA from all intestinal fragments are intact except for the Duodenum, which is slightly

Powered by Editorial Manager® and ProduXion Manager® from Aries Systems Corporation

degraded. The microbiota DNA from all intestinal fragments are qualified for HiFi sequencing.

The authors state that computational limitations prevented them from doing a co-assembly, but did they include coverage data from multiple gut regions while binning their non-circular contigs with metabat2? Using many samples from different individuals and regions of the gut aligned to the contigs you are binning can improve the outcome from binning and uses more data without requiring the extreme computational power of the assembly stage.

Reply: Thanks for the reviewer’s suggestion. We agreed that using many samples from different individuals and regions of the gut may improve the binning process. If we have taken a co-assembly of all the intestinal data and generated one contig data set, then that will be a certain choice.

However, a big limitation in metagenome assembly is the computer memory, for example, only the assembly of cecum data needs about 800 G memory, and it will take over 3000 G memory for a co-assembly of all the 5 intestinal fragments. Considering that the maximum memory of our computer server is 1000 G, we assembled each of the 5 intestinal fragments separately, resulting in 5 hifiasm-meta contig data sets. Then, a major task is to merge them into one assembly result. If we merge all the contigs firstly, then the linkage and depth information from the hifiasm-meta GFA files will be lost.

To fully utilize the linkage and depth information from the hifiasm-meta GFA file, we decided to get genome-level assembly for each of the 5 intestinal fragment firstly, including 3 parts: (1) the circular contigs were taken as complete genome; (2) “tangled”circular were re-assembled into non-redundant contig assembly; (3) linear contig were binned by Metabat2 using the depth information obtained from the hifiasm-meta GFA file of the corresponding intestinal fragment. In this way, the contigs and the depth information matched well. Finally, the genome-level assemblies from all the 5 intestinal fragments were merged into a non-redundant genome-level assembly. We think this strategy is more suitable for our data analysis. Although the results here may not be the best, but it should be reliable.

There are a couple of instances of GTDB-tk being written as GTDT-tk, please double check this is correct throughout.

Reply: We have corrected all “GTDT-tk”to “GTDB-tk”.

I find the panel order in figure 5 to be unintuitive, I suggest that the panels below A and B go in order from left to right rather than top to bottom.

Reply: We have re-ordered the panel order of figure 5, let c, d, e in one row, and f, g, h in another row.

The last paragraph of page 7 is a little fiddly to follow, it might be better presented as a table or just referring the reader to see the data in the figure.

Reply: We have added a supplementary Table (Table S5) to make it easy to follow:

Table S5. Unclassified number of genomes at each taxonomic level
Note: Here the 337 species-level microbial genomes were used as input, and the unclassified number of genomes at each taxonomic levels (family, genus, species) were shown by three classification methods: (1) GTDB-tk alone; (2) GTDB-tk and Ribosomal Database Project (RDP) Classifier; (3) GTDB-tk and Ribosomal Database

Powered by Editorial Manager® and ProduXion Manager® from Aries Systems Corporation

	Project (RDP) Classifier and alignments to the Silva 16S rRNA database. The last method classified the maximum number of genomes, and only 9 and 49 genomes failed to be classified into known genus and species, indicating that they may be novel genus and species which haven't been reported before.<br><br>Page 7 refers to "figure c-d" without a figure number.<br><br>Reply: "figure c-d" has been changed to “"Figure 6c-d"”.<br><br>I like figure 7, but a couple of parts are difficult to interpret. The circles and squares are not very distinct, is it possible for the squares to be another shape that is less similar to the circles? With the aid of zooming in it is possible to read the labels on the depth tracks, but for convenience I would recommend also listing the labels in order in the caption.<br><br>Reply: We have used hollow circles to replace the squares, and used solid circles to replace the circles. We also listed the labels in order in the caption “From inner to outer: duodenum, jejunum, ileum, cecum, and colorectum.”.<br><br>I find this sentence on page 10 a bit ambiguous and suggest adjusting for clarification "Finally, the combined DNA samples from 30 chicken individuals for duodenum, jejunum, ileum, cecum, and colorectum were generated independently." (i.e. what is combined and what is independent)<br><br>Reply: For each intestinal compartment, all the extracted DNA from all chickens were combined. The digesta samples from different intestinal compartment were processed separately. Finally， there were only 5 metagenomic DNA samples of duodenum, jejunum, ileum, cecum, and colorectum. The manuscript was revised for a better clarification：<br><br>“Mainly due to the volume of digesta, it was difficult to process all of the samples at one time. For the convenience of processing, the duodenum digesta from every 5 chickens were pooled together and then washed for microbial cell enrichment and DNA extraction. After processing all duodenum samples, the metagenomic DNA was finally pooled, and further purified with VAHTS DNA Clean Beads (N411-02, Vazyme). The metagenomic DNA samples of the jejunum, ileum, cecum and colorectum were processed in the same way, except that for the cecum, due to its relatively high microbial density, only a subfraction of the pooled and thoroughly mixed digesta was used for microbial cell enrichment and DNA extraction.”<br><br>Can the authors clarify in the methods section the method of bead beating used in the protocol, for reproducibility.<br><br>Reply: The details about bead beating were added into the manuscript：“For the bead beating and lysis options of the DNeasy PowerSoil Pro kit, we added approximately 200 mg of the enriched cells and 800 uL of Solution CD1 into each PowerBead Pro Tube. The tubes were vortexed briefly to mix and incubated at 65 °C for 10 min before the bead beating step. Then, the tubes were placed horizontally and properly balanced on a Vortex Adapter for 24 (1.5–2.0 ml) tubes (QIAGEN, 13000-V1-24) on a Kylin-Bell VORTEX-6. The samples were vortexed in the tubes at maximum speed for 10 min. To ensure the efficiency of the homogenization step, fewer than 12 tubes were vortexed at one time. All the other steps were carried out according to the manufacturer's standard protocol.”
**Additional Information:**	
**Question**	**Response**
Are you submitting this manuscript to a	No

special series or article collection?	
**Experimental design and statistics**<br><br>Full details of the experimental design and statistical methods used should be given in the Methods section, as detailed in our Minimum Standards Reporting Checklist. Information essential to interpreting the data presented should be made available in the figure legends.<br><br>Have you included all the information requested in your manuscript?	Yes
**Resources**<br><br>A description of all resources used, including antibodies, cell lines, animals and software tools, with enough information to allow them to be uniquely identified, should be included in the Methods section. Authors are strongly encouraged to cite Research Resource Identifiers (RRIDs) for antibodies, model organisms and tools, where possible.<br><br>Have you included the information requested as detailed in our Minimum Standards Reporting Checklist?	Yes
**Availability of data and materials**<br><br>All datasets and code on which the conclusions of the paper rely must be either included in your submission or deposited in publicly available repositories (where available and ethically appropriate), referencing such data using a unique identifier in the references and in the "Availability of Data and Materials" section of your manuscript.<br><br>Have you have met the above requirement as detailed in our Minimum Standards Reporting Checklist?	Yes

Powered by Editorial Manager® and ProduXion Manager® from Aries Systems Corporation

Manuscript

Click here to access/download;Manuscript;manuscript_Revison1_20220920

Improved microbial genomes and gene catalog of the chicken gut from metagenomic sequencing of high-fidelity long reads

Formatted: Numbering: Continuous

Yan Zhang*, Fan Jiang*, Boyuan Yang*, Sen Wang, Hengchao Wang, Anqi Wang, Dong Xu, and Wei Fan

Guangdong Laboratory for Lingnan Modern Agriculture (Shenzhen Branch), Genome Analysis Laboratory of the Ministry of Agriculture and Rural Affairs, Agricultural Genomics Institute at Shenzhen, Chinese Academy of Agricultural Sciences, Shenzhen, Guangdong, 518120, China.

*These authors contributed equally to this work. Correspondence should be addressed to fanwei@caas.cn.

Formatted: Font: (Default) Times New Roman

Abstract

Background: Due to the importance of chicken production and the remarkable influence of the gut microbiota on ~~the~~ host~~'s~~ health and growth, tens of thousands of metagenome-assembled genomes (MAGs) have been constructed for the chicken gut microbiome. However, ~~limited by~~due to the limitations of ~~the~~ short-read sequencing and assembly technologies, most of these MAGs are far from complete, are of lower quality, and include contaminant reads~~and have lower qualities with contamination~~.

Results: We generated 332 Gb of high-fidelity (HiFi) long reads from the five chicken intestinal compartments~~,~~ and assembled 461 and 337 microbial genomes~~ at species and strain level~~, of which 53% and 55% are circular~~ genomes~~, at the species and strain levels, respectively. For the assembled microbial genomes, approximately~~about~~ 95% were regarded as complete according to the "RNA complete" criteria, which requires at least one full-length rRNA operon encoding ~~for~~ all three types of rRNA (16S, 23S and 5S~~ rRNA~~) and at least 18 copies of full-length tRNA genes. In comparison with the short-read-derived chicken MAGs, 384 (83% of 461) and 89 (26% of 337) strain-level and species-level genomes in this study are novel, with no matches to previously reported sequences ~~and do not have any matches~~. At the gene level, one~~a~~-third of the 2.5 million genes in the HiFi-derived gene catalog are novel and cannot be matched to the short-read-derived gene catalogs. Moreover, the HiFi-derived genomes have much higher continuity and completeness, as well as lower contamination; the HiFi-derived gene catalog has a much higher ratio of complete gene

Commented [Ed1]: Please ensure that the intended meaning has been maintained in this edit.

Formatted: Font: (Default) Times New Roman

structures. The dominant phyluma~~a~~ in our HiFi-~~-~~assembled genomes ~~is~~ was Firmicutes (82.5%), and the foregut ~~is~~ was highly enriched in five genera *Ligilactobacillus*, *Limosilactobacillus*, *Lactobacillus*, *Weissella*, and *Enterococcus*, all of which belong~~s~~ to the order Lactobacillales. Using ~~GTDB-tk~~GTDB-Tk, all ~~the~~ 337 species-level genomes were successfully classified ~~to~~ at the order level;~~,~~ however, 2, 35 and 189 genomes could not be classified into any known family, genus, and species, respectively. Among these incompletely~~not fully~~ classified genomes, ~~9~~ 9 and ~~49~~ 49 ~~of them~~ may belong to novel genera and species, respectively, ~~for that~~because their 16S ~~-~~rRNA genes have identities lower than 95% and 97% to any known 16S ~~-~~rRNA genes.

Conclusions: HiFi sequencing not only ~~remarkably improved the quality of~~produced metagenome assemblies and gene structures with markedly improved quality, but also recovered a substantial portion of novel genomes and genes that were missed in previous short-read-based metagenome studies. The novel genomes ~~or novel~~and species obtained in this study will facilitate ~~the~~ gut microbiome and host-~~-~~microbiota interaction studies, ~~which would benefit~~and thereby contribute to the sustainable development of poultry resources.

Introduction

The d~~D~~omestic chicken, *Gallus gallus*, has long been used as a model ~~animal for~~ avian species, and chicken eggs and meat provide a primary source of animal-derived protein in the human diet. The first draft genome sequence of chicken was published in 2004, providing unique perspectives on vertebrate evolution (International Chicken Genome Sequencing, 2004). Subsequent~~Then, the~~ population resequencing studies revealed not only the phylogeny history and population structure~~,~~ of this species but also information about~~the~~ locus~~i~~ selection during chicken domestication (Rubin, et al., 2010; Wong, et al., 2004). The gut microbiota can degrade dietary polysaccharides, detoxify xenobiotics, produce nutrients and energy sources~~ies~~ such as vitamins, amino acids, short-~~-~~chain fatty acids (SCFA), and can also modulate the immune system, thus playing important roles in chicken nutrition, physiology, immunity, and health. However, the gut microbiota also contains many zoonotic pathogens, posing threats to the poultry industry and to human health (Oakley, et al., 2014; Yeoman, et al., 2012). Due to the importance of the chicken gut microbiota, ~~their~~ its compositions and host-interactions~~s~~ ~~with the host~~ have been ~~intensively~~ studied intensively in ~~the past~~recent years.

High-throughput short-read sequencing technologies have extensively facilitated metagenome studies to explore the taxonomic and functional compositions of the chicken gut microbiota. Studies that aim to decipher ~~the~~ taxonomic compositions tend~~s~~ to sequence 16S rRNA gene amplicons (Sergeant, et al., 2014; Wen, et al., 2019), while studies that focus on both taxonomy and functions ~~will adop~~have used~~t~~ whole-~~-~~genome shotgun sequencing (Feng, et al., 2021). In 2018, Huang et al. ~~firstly~~ constructed the first~~a~~ comprehensive gene catalog of the chicken gut microbiome containing ~9 million genes~~,~~ through sequencing of 495 chicken samples from seven different farms in China (Huang, et al., 2018). Then, several endeavors have been made to construct the metagenome assembled genomes (MAGs) from the ~~fragmental~~fragmented contigs. In 2020, Glendinning et al. constructed 469 draft MAGs using the gut metagenomes of 24 chicken samples (Glendinning, et al., 2020). In 2021, Segura-Wang et al. reconstructed 155 MAGs from metagenomes of 751 chicken

Formatted: Font: (Default) Times New Roman

samples (Segura-Wang, et al., 2021); Gilroy et al. constructed over 5,595 MAGs based on 632 chicken metagenomes (Gilroy, et al., 2021); Feng et al. assembled 12,339 MAGs by integrating 799 public chicken gut microbiome samples from ten countries (Feng, et al., 2021). These MAGs and gene catalogs constructed from short-read metagenome data provide an overview of the chicken gut microbiota landscape.

Due to the technical limitation of short-read sequencing, these metagenome assemblies~~y~~ often ~~results in~~produce fragmented~~tal~~ contigs, with a contig N50 less than 10~~ Kb~~ kb, and a certain ~~part~~ portion of ~~small ~~contigs that are less than 500 bp in length are usually excluded for downstream analyses ~~analysis ~~(Huang, et al., 2018). Although these short contigs can be grouped into MAGs with binning algorithms, binning introduces several types of errors, such as incompleteness and contamination (Yue, et al., 2020). Therefore, MAGs cannot be taken as microbial reference genomes. Indeed, a considerable portion of the gene structures in the non-redundant gene catalog are in~~not~~ complete, limiting their use ~~for potential~~in various applications. The advent of highly accurate long-read HiFi (high-fidelity) sequencing ~~provides a ~~promises ~~for ~~to resolve~~ing~~ these problems. Recently, a sheep fecal metagenome study using ~200 Gb HiFi read~~s~~ data assembled by metaFlye produced ~~assembled ~~44 circular contigs, each corresponding to a complete reference genome (Bickhart, et al., 2022; Kolmogorov, et al., 2020). Furthermore, using the same data, ~~the software of ~~Hifiasm-meta software generated even better assembly result, producing~~of~~ 279 circular complete reference genomes (Feng, et al., 2022). In this study, we used high-fidelity long-~~ ~~read technology~~s~~ to improve the metagenome assemblies and gene catalogs of the chicken gut microbiomes.

Results

Longer contigs of the chicken metagenome assembled from high-fidelity long reads

We collected 150 digesta samples from the five intestinal compartments (duodenum, jejunum, ileum, cecum, and colorectum) of 30 chickens (Lingnan yellow broilers) slaughtered on ~~day ~~Day 42, extracted the metagenomic DNA and~~,~~ combined the DNA samples, evaluated the DNA quality and quantity (Table S1 and Figure S1), and constructed sequencing libraries for each intestinal compartment. Then, we generated 22 Gb, 45 Gb, 73 Gb, 81 Gb, and 112 Gb PacBio HiFi reads for duodenum, jejunum, ileum, cecum, and colorectum, respectively (Table 1). For the total 332 Gb HiFi reads, the N50 read length is 17 k~~K~~b, and the median read quality value is 32;~~,~~ ~~which ~~these values are comparable to those of previous HiFi metagenome studies (Bickhart, et al., 2022; Feng, et al., 2022). The increasing amount of HiFi reads from the duodenum to the colorectum was associated with the increase in microbial diversity ~~of ~~along the different intestinal compartments (Huang, et al., 2018), permitting the~~in order to~~ recovery of more microbial species.

We assembled the HiFi reads into contigs for each intestinal compartment independently ~~by ~~with Hifiasm-meta (Feng, et al., 2022), which ~~results in~~produced linkage graphs of the contigs. Taking the colorectum as an example, we ~~saw ~~observed a single "super complex", several tangled circular~~circular~~, hundreds of circular, and ~~a lot of~~many linear topologies in the contig graph (Figure 1, Figure S2~~1~~). Upon reviewing~~By checking~~ the taxonomic components and read~~s~~ coverage depth for each topology, we found that the super complex contains tens of various microbial genomes sharing some similar genomic fragments; the tangled circles contain many different strains of one species, and the high redundancy of overlapped contigs makes the tangled circles seem~~s~~ much

~~bigger~~ larger than the real genome size of the species ~~genome~~; and the circular and linear contigs represent complete and incomplete genomes for ~~a~~ single microbial strain or species, respectively.

The total contig sizes are 0.22 Gb, 0.56 Gb, 0.85 Gb, 3.11 Gb, and 3.96 Gb and the contig N50 sizes are 28 ~~Kb~~ kb, 29 ~~Kb~~ kb, 34 ~~Kb~~ kb, 193 ~~Kb~~ kb and 165 ~~Kb~~ kb for the duodenum, jejunum, ileum, cecum, and colorectum, respectively (Figure 2ab, Table S2~~1~~). In comparison, the contig N50 sizes from short-read metagenome ~~assembly~~ assemblies are~~is~~ usually lower than 10 ~~Kb~~ kb (Huang, et al., 2018), suggesting that HiFi reads assembly provides a substantial improvement in~~has largely improved the~~ contig continuity. The foregut (duodenum, jejunum, ileum) assemblies contain more fragmented~~al~~ contigs than the hindgut (cecum, colorectum), which may be explained by the fact that the foregut contains only a few of dominant microbial species and other species with very low abundance. In comparison, the hindgut (cecum, colorectum) contains hundreds of abundant microbial species, and their abundance distribution ~~are~~ is relatively more even. ~~Though~~ Although genomic complexity may also lead to ~~fragmental~~ fragmented contigs, we observed a non-~~-~~trivial correlation between contig size and coverage depth, indicating that insufficient coverage depth of microbes with very low abundance is the primary reason for most of the ~~fragmental~~ fragmented contigs (Figure 2c, Figure ~~S2~~S3). Moreover, the coverage depth is positively related with the single-base quality values, indicating that higher coverage depth will improve the single-base accuracy of the contig sequences (Figure 2d).

Hundreds of complete circular genomes and binned non-circular MAGs

For the duodenum, jejunum, ileum, cecum, and colorectum, respectively, we obtained 22, 25, 41, 120 and 173 reference microbial genomes of circular contigs ~~,~~ and recovered 5, 15, 21, 165 and 161 metagenome-assembled genomes (MAGs) from the binning of non-circular contigs, resulting in a total of 27, 40, 62, 285 and 334 assembled microbial genomes that passed the medium-quality criteria~~, respectively~~ (Figure 3a, Table ~~S2~~S3). Most of the circular genomes met the near-complete criteria, while the non-circular MAGs include more ~~of those~~genomes with relatively lower qualities, referred to as high-quality and medium-quality. Previously, the Hifiasm-meta project ~~has~~ used a small portion of ~~our~~ the data generated in this study for software testing~~,~~ and assembled 62 circular microbial genomes that ~~meets~~ met near-complete criteria using 33.6 Gb of chicken cecum data (Feng, et al., 2022). In this study, using a total of 81 Gb of cecum data, we successfully assembled 110 circular microbial genomes with near-complete quality. This result indicates that more complete genomes can be assembled by increasing the sequencing depth.

For the assembled microbial genomes within each intestinal compartment, the sequence divergences are mostly above 1%. i.e. ~~with~~ have an average nucleotide identity (ANI) below 99%, which represents ~~for~~ a strain-level assembly. To remove the assembly redundancy among intestinal compartments, we removed redundant genomes ~~with the requirement of~~(those with a sequence divergence ~~-sequence divergence~~ lower than 1%) and ~~then~~ thereby generated 461 non-redundant genomes of microbial strains ~~for~~ within the chicken gut (Figure 3a). Furthermore, to remove the redundant genomes at the species~~-~~ level, these 461 non-redundant microbial strain genomes were reduced to 337 non-redundant genomes with sequence divergences ~~above~~ greater than 5%. Of the 461 strain-level and 337 species-level microbial genomes, 246 (53%) and 187 (55%) are circular genomes, respectively. According to the distribution analysis, the circular genomes have larger

Formatted: Font: (Default) Times New Roman, 10.5 pt
Formatted: Font: (Default) Times New Roman, 10.5 pt
Formatted: Font: (Default) Times New Roman, 10.5 pt
Formatted: Font: (Default) Times New Roman, 10.5 pt
Formatted: Font: (Default) Times New Roman, 10.5 pt
Formatted: Font: (Default) Times New Roman, 10.5 pt
Formatted: Font: (Default) Times New Roman, 10.5 pt
Formatted: Font: (Default) Times New Roman, 10.5 pt
Formatted: Font: (Default) Times New Roman, 10.5 pt
Formatted: Font: (Default) Times New Roman, 10.5 pt
Formatted: Font: (Default) Times New Roman, 10.5 pt
Formatted: Font: (Default) Times New Roman, 10.5 pt
Formatted: Font: (Default) Times New Roman, 10.5 pt
Formatted: Font: (Default) Times New Roman, 10.5 pt
Formatted: Font: (Default) Times New Roman, 10.5 pt
Formatted: Font: (Default) Times New Roman, 10.5 pt
Formatted: Font: (Default) Times New Roman, 10.5 pt
Formatted: Font: (Default) Times New Roman
Formatted: Font: (Default) Times New Roman, 10.5 pt
Formatted: Font: (Default) Times New Roman, 10.5 pt
Formatted: Font: (Default) Times New Roman, 10.5 pt
Formatted ...
Formatted ...
Formatted ...
Formatted ...
Formatted ...
Formatted ...
Formatted ...
Formatted ...
Formatted: Font: (Default) Times New Roman
Formatted: Font: (Default) Times New Roman
Formatted: Font: (Default) Times New Roman

assembly sizes and higher checkMCheckM scores in comparison withthan the non-circular MAGs (Figure 3bc), and the assembled genome sizes have positive correlationsare positively correlated with the checkMCheckM scores (Figure S3S4). Using the 187 circular species-level genomes, which all have complete genome assemblies, we showed that higher coverage depth is positively correlated with CheckM completeness score, indicating that a higher coverage depth will improve the single-base accuracy of the genome assemblies (Figure S5).

Although plasmids were reported to be more difficult to assemble than host genomes in metagenomes (Pellow, et al., 2021), we were able to identify 61, 67, 71, 81 and 78 circular plasmid genomes in the Hifiasm-meta contigs for duodenum, jejunum, ileum, cecum, and colorectum, respectively (Table S43). Moreover, we also identified 33, 14, 14, 52 and 50 circular viral genomes for among the corresponding intestinal compartments. The average genome size of the plasmid genome sizes is 69 Kb kb, which is a littleslightly larger than that ofe average the virus genome size ofs 52 Kb kb. The success in assembling these circular plasmid and virus genomes is encouraging;, manyand much more plasmid and virus fragments exist in the tangled or linear contigs and should be investigated further, which needs further investigation.

Formatted: Font: (Default) Times New Roman

The pPresence of rRNA and tRNA genes confirms the high assembly quality

In prokaryotes, the 5S, 16S, and 23S ribosomal RNA (rRNA) genes are commonly colocatedlocated and transcribed together, forming rRNA operons. Usually, mMultiple copies of rRNA operons exist in one genome, and the repetitive characteristic nature makes rRNA operonsthem difficult to assemble with from short reads. Transfer RNA (tRNA) genes are randomly distributed in the genome, often with in multiple redundant copies. The identification of rRNA and tRNA genes was has traditionally been used as an important measurement for the completeness of genome assembly (Feng, et al., 2022). We annotated the rRNA and tRNA genes in the 461 non-redundant microbial genomes, and found that 447 (97%) genomes have at least one full-length rRNA operon encoding for all three types of rRNA (5S, 16S, 23S) genes, 450 (98%) genomes have at least 18 copies of full-length tRNA genes, and 439 (95%) genomes are "RNA complete", which meeting boths the requirements of both rRNA and tRNA criteria. Our results showed that most microbial genomes have 1-6 rRNA operons (Figure 4a), and 35-65 copies of tRNA genes (Figure 4b). In addition, the number of rRNA operon and tRNA genes in circular genomes is larger than that on in non-circular MAGs (Figure 4ab), which is consistent with the results of the completeness comparison ofanalysis of the microbial genomes.

Superiority of HiFi assembled genomes over short-read assembled MAGs

Numerous endeavors efforts have been made to construct MAGs from short-read assembled contigs (Kang, et al., 2019). A recent study on the chicken gut metagenome reported the generation of 12,339 dereplicated strain-level MAGs (ANI < 99%) and 1,978 dereplicated species-level MAGs (ANI < 95%), by integrating the short-read assembly of 799 public chicken gut microbiome samples from ten countries (Feng, et al., 2021). Comparing Compared to the reported strain-level MAGs, 384 (83%) of our 461 strain-level genomes are novel (ANI < 99%), including 209 (45%) circular genomes and 175 (38%) non-circular MAGs (Figure 5a). Comparing Compared to the reported species-level MAGs, 89 (26%) of our 337 species-level genomes are novel (ANI < 95%), including 50 (15%) circular genomes and 39 (12%) non-circular MAGs (Figure 5b). Although the currently

limited sample sizes and HiFi sequencing depth ~~have led to~~produces ~~the~~ a smaller number of assembled microbial genomes than ~~that of~~are generated by the short-read assembl~~ed MAGs~~y (Figure 5c), HiFi assembly can recover genomes of novel species and especially novel strains, which cannot be successfully ~~be~~ resolved by short read~~s~~ assembly, ~~due to fact that~~because short reads cannot distinguish the highly similar sequences ~~between~~ of closely related microorganisms~~ with close relationships~~.

The quality of the HiFi assembled microbial genomes ~~have huge superiorities over~~is highly superior to that of the short-read assembled MAGs. The average contig numbers for our assemblies are~~is~~ 1 for our circular genomes and 2.8 for non-circular MAGs, in comparison to 257 for the short-read assembled MAGs (Figure 5d). ~~The~~ Our average assembled genome sizes are 2.61 Mb, 2.35 Mb, and 2.23 Mb, and the average contig N50 sizes are 2,884~~ Kb~~ kb, 1,697~~ Kb~~ kb and 38~~ Kb~~ kb for the circular genomes, non-circular MAGs, and short-read MAGs, respectively (Figure 5e-f). Moreover, the average ~~checkM~~CheckM completeness percentages are 95.5, 76.4 and 89.5, and the average ~~checkM~~CheckM contamination percentages are 0.85, 1.59, and 2.14 for the circular genomes, non-circular MAGs, and short-read MAGs, respectively (Figure 5g-h). Almost all the evaluations of our circular genomes and non-circular MAGs are better or much better than those of the short-read assembled MAGs, except for the ~~checkM~~CheckM completeness of our non-circular MAGs, which is ~~a little~~slightly lower than that of the short-read MAGs, because ~~that~~ the two genome datasets ~~have~~ used different completeness cutoffs (50% versus 80%). Overall, the HiFi-~~-~~assembled microbial genomes are not only more continuous and complete than the short-read MAGs ~~,~~ but also have less contamination.

Advantage of HiFi-derived gene catalog over gene catalogs from short-reads

~~Besides~~ In addition to MAGs, the non-redundant gene catalog ~~was~~ is another important resource in metagenome studies. Based on Illumina sequencing data, in 2018, Huang et al. published the first 9.0 M gene catalog (CGM-RGC) for the chicken gut metagenome ~~in 2018~~ (Huang, et al., 2018), and in 2021, Feng et al. published a more comprehensive~~n~~ ~~integrated~~ 16.6 M gene catalog (GG-IGC) ~~for the chicken gut metagenome in 2021~~ (Feng, et al., 2021) that integrated all the available public chicken metagenome sequencing data. Here, we constructed a 2.5 M non-redundant gene catalog (HiFi-RGC) with the HiFi assembled contigs from all intestinal compartments. Alt~~T~~hough the gene number of our gene catalog is smaller than those of the two published gene catalogs due to the limited sample sizes, the structure completeness ratio of our gene catalog is 99%, much higher than the 38% and 63% reported for CGM-RGC and GG-IGC, respectively (Figure 6a-b).

By comparing the pairwise overlap at the gene sequence level, we found that 847,801 (33.8%) and 724,123 (28.9%) genes are unique in HiFi-RGC compared to CGM-RGC and GG-IGC, respectively (Figure 6c-d)~~According to the gene sequence analysis, 847,801 (33.8%) and 724,123 (28.9%) genes are novel compared to CGM-RGC and GG-IGC (Figure c-d)~~, suggesting that the HiFi-~~-~~derived gene catalog ~~has~~ recovered a substantial portion of the genes that ~~are~~ were missed by short-read technologies. Because GG-IGC is more comprehensive than CGM-RGC, we considered the 724,123 (28.9%) genes in HiFi-RGC as unique genes and the remaining genes (71.1%) in HiFi-RGC as shared genes.
Then, the microbial communities derived from the unique and shared genes in HiFi-RGC were

compared. The results showed that 36.8% of unique genes were unclassified at the phylum level, which was obviously higher than the proportion of shared genes (24.9%), suggesting that the unique genes are enriched in unknown phyla (Figure S6).

Phylogeny of HiFi assembled microbial genomes and differences among intestinal compartments~~and novel genomic representation~~

We used ~~GTDB-tk~~GTDB-Tk to align the 337 HiFi assembled species-level genomes to the 47,894 species clusters (45,555 bacterial and 2,339 archaeal) in the GTDB database (r202)~~,~~ and assign taxonomic classification to the HiFi assembled genomes based on their phylogenetic placement (Chaumeil, et al., 2019). Only one genome was classified ~~to~~ as archaea, and the other 336 genomes were all classified ~~to~~ as bacteria. The dominant phylum is Firmicutes containing 278 (82.5%) genomes, followed by Bacteroidota and Actinobacteriota, which contain 25 (7.4%) and 14 (4.2%) genomes, respectively. In total, these three phyla covered 317 (94%) of all the assembled genomes. The remaining genomes were classified as Cyanobacteria, Proteobacteria, Desulfobacterota, Campylobacterota, Deferribacterota, Methanobacteriota, and Verrucomicrobiota.

The foregut contains the duodenum, jejunum, and ileum, which mainly function in feed digestion and nutrient absorption. The hindgut contains the cecum and colorectum, which function in fermentation, detoxification and recycling of residual water and salt. Noticeably, there was a distinctive difference in the microbial composition between the foregut ~~(duodenum, jejunum, ileum)~~ and hindgut. ~~(cecum, and colorectum) in microbial composition.~~ The foregut was highly enriched in five ~~genus~~ genera *Ligilactobacillus*, *Limosilactobacillus*, *Lactobacillus*, *Weissella*, and *Enterococcus*, all belonging to the order ~~of~~ Lactobacillales. In contrast, the species diversity of the hindgut was much higher, and the species ~~was~~ were more dispersed (Figure 7, ~~Figure S4~~Figure S7). Th~~is~~ise difference ~~of~~ in species composition between the foregut and hindgut ~~was~~ is consistent with previous reports from short-read metagenome studies (Huang, et al., 2018), ~~which was~~and is caused by the difference in morphology and physiology between the foregut and hindgut. The sampling of all intestinal compartments contributes to more comprehensive microbial genome assemblies. Microbes of very low abundances in some intestinal compartments but of relatively higher abundance in other compartments could also be recovered ~~(Figure 7)~~.

~~(Sarao and Arora, 2017)(Sun, et al., 2015)~~

Novel genomic representation and novel genus and species discovery

Although all the species-level genomes have been successfully classified at the order level by GTDB~~T~~-T~~-t~~k (Chaumeil, et al., 2019), 2, 35 and 189 genomes ~~cannot~~ could not be classified at the lower taxonomic levels of family, genus, and species, respectively, suggesting that they are novel ~~assembled~~ genome assemblies for these families, genera, and species (Figure 7~~, Figure S4~~). Some of these new genomic sequences may have potential benefits to industry or medical applications. *Lactobacillus* has traditionally been used in the fermentation industry, producing lactate from raw carbohydrates and synthetic media (Sun, et al., 2015). In recent years, *Lactobacillus* and its close relatives *Ligilactobacillus* and *Limosilactobacillus* have also been widely adopted as probiotic supplements, either in animal feed to promote growth or human foods to improve human health (Sarao and Arora, 2017). Among our 337 assembled microbial species genomes, 3 genomes belong

Formatted: Not Highlight

Formatted: Font: (Default) Times New Roman, 10.5 pt

Formatted: Font: (Default) Times New Roman, 10.5 pt

Formatted: Don't adjust space between Latin and Asian text, Don't adjust space between Asian text and numbers

Formatted: Font: (Default) Times New Roman, 10.5 pt

Formatted: Font: (Default) Times New Roman, 10.5 pt

Formatted: Font color: Custom Color(RGB(19,20,19)), Pattern: Clear

Formatted: Font: Bold

to *Lactobacillus*, 7 genomes belong to *Ligilactobacillus*, and 6 genomes belong to *Limosilactobacillus*. All these genomes have been successfully classified to the genus level, and most of the genomes were successfully classified to the species level by GTDB-Tk. However, 2 *Ligilactobacillus* genomes and 1 *Limosilactobacillus* genome have not been classified to the species level, suggesting that these 3 species-level genomes may represent novel genomic resources for probiotic development.

To further classify these genomes at lower taxonomic ranks, we ~~also~~ used the Ribosomal Database Project (RDP) Classifier and alignments to the Silva 16S rRNA database with the annotated 16S rRNA gene sequences from their genomes. RDP successfully classified one genome at the family level~~,~~ and 14 genomes at the genus level, leaving 1, 21, and 189 genomes still unclassified at the family, genus, and species levels, respectively (Table S5). Then, the alignment identities to the Silva database were used to validate the taxonomic novelty for these genomes. We found that ~~58~~ 58 genomes have 16S-rRNA gene identities lower than 97%, which is the threshold for demarcating bacterial species (Stackebrandt and Goebel, 1994)~~.~~; ~~A~~among these, ~~which~~ ~~9~~ 9 genomes have 16S-rRNA gene identities lower than 95%, which is the threshold generally used to delineate a new genus (Ludwig, et al., 1998), indicating that ~~9~~ 9 and ~~49~~ 49 of these genomes may~~ be~~ correspond~~ing~~ to novel genera~~us~~ and species, respectively, which broadens our knowledge of the microbial world (Table S6-7). In addition, approximately one-third of these newly discovered genera and half of these newly discovered species were not found in the short-read MAG data, suggesting that they are derived only from HiFi metagenome data, which further shows the advantage of HiFi sequencing in metagenomic studies.

Conclusions

Given the importance of chicken production and the remarkable contribution of the intestinal microbiota to ~~the~~ host nutrition and health, numerous ~~endeavors~~ efforts have been made to construct chicken gut MAGs and gene catalogs. In the present study, using high-fidelity long reads of the five intestinal compartments of chickens, we assembled 461 microbial genomes at strain level (ANI > 99%) and 337 microbial genomes at species level (ANI > 95%), of which 246 (53%) and 187 (55%) are circular genomes, respectively~~, using high-fidelity long reads of the five intestinal compartments of chickens~~. In addition, many circular plasmids and viral genomes were also successfully obtained. Among the 461 microbial genomes, 439 (95%) genomes are “RNA complete”~~, which meet the criteria of~~ having at least one full-length rRNA operon coding for all three types of rRNA (16S, 23S and 5S rRNA) genes and at least 18 copies of full-length tRNA genes. ~~Besides human and sheep~~With this work, chicken is now the third animal species after human and sheep that have comprehensive HiFi gut metagenome assemblies.

In comparison to the chicken MAGs derived from short-read metagenome assemblies, the HiFi-assembled microbial genomes not only ~~have huge~~provide substantial advantages in continuity, completeness, and contamination metrics, but also recovered 384 (83% of 461) and 89 (26% of 337) novel strains and species, respectively. In addition, the structure completeness ratio of the 2.5 M non-redundant gene catalog constructed from HiFi assembled contigs (>99%) is much higher than

Formatted: Font: (Default) Times New Roman, 10.5 pt
Formatted: Font: (Default) Times New Roman, 10.5 pt
Formatted: Font: (Default) Times New Roman, 10.5 pt
Formatted: Font: (Default) Times New Roman, 10.5 pt
Formatted: Font: (Default) Times New Roman, 10.5 pt
Formatted: Font: (Default) Times New Roman, 10.5 pt
Formatted: Font: (Default) Times New Roman, 10.5 pt
Formatted: Font: (Default) Times New Roman, 10.5 pt
Formatted: Font: (Default) Times New Roman, 10.5 pt

that of the short-read assembly derived gene catalogs (40-60%), and approximatelyabout one--third of the genes in the HiFi--derived gene catalog are novel compared tonot present in the short-read derived gene catalogs. Taken together, our results showed that HiFi metagenome sequencing not only bring yields genomes and genes with better qualities, but also providesd a substantial portion number of novel genomes and genes that were missed in short-read metagenome studies.

Phylogeny analysis showed that the dominant phyla in our HiFi assembled genomes are Firmicutes (82.5%), Bacteroidota (7.4%) and Actinobacteriota (4.2%). The foregut is highly enriched in five generaus in Lactobacillales (order), *Ligilactobacillus*, *Limosilactobacillus*, *Lactobacillus*, *Weissella*, and *Enterococcus* in Lactobacillales (order), whereas the hindgut has a much wider spectrum of species. Using GTDT-tkGTDB-Tk, 2, 35 and 189 genomes failed to be classified at the family, genus, and species levels, suggesting that they are novel assembled genomes at these respective levelsfor these family, genus, and species, respectively. The RDP CClassifier further assigned one genome at the family level, and 14 genomes at the genus level. Among the remaining unclassified genomes, 9 9 and 49 49 genomes have 16S -rRNA gene identities lower than 95% and 97% to in the Silva database, indicating that these genomes may represent for novel generaus and species, respectively. The HiFi metagenome assembly not only improves the genomic representation, but also enables the discovery of novel taxonomic units. With regard to the chicken production, these novel microbial genomes or species will serve as a valuable resource for functional future studies of functions, such as feed digestion and, fermentation, as well as the mechanisms of disease-prevention and growth-promotion effects of antibiotics and alternatives.

Methods

Chickens husbandryraising and disease prevention

The Lingnan yellow broilers were studied for a 42-day feeding trailtrial, with free access to feed and water. The baby chicks were bought purchased from Zhiwei Guangdong company at 1 day of1 age, and raised in battery cages at the farm–house of the Agricultural Ggenomics Iinstitute of Shenzhen. The lighting schedule was 16 h light and 8 h dark throughout the experiment. The room temperature was controlled with heaters, gradually reduced from 35 °C on day Day 1 to 24 °C on day Day 21, and then maintained at 24 °C do not change until day Day 42. The diets were based on the Nutrient Requirements of Poultry: Ninth Revised Edition, 1994 (NRC, 1994) and Feeding Standard of Chicken (NY/T 33-2004).

The chickens were injected with Marek's Disease Vaccine and Cephalosporin on day Day 1, vaccinated againstwith Newcastle disease virus (NDV, La Sota) and infectious bronchitis virus (IBV, H120) on day Day 7 through intranasal administration, vaccinated with against NDV La Sota and IBV M41 and avian influenza H9-NJ02 on day Day 9 through hypodermic injection, vaccinated againstwith iInfectious bBursal dDisease vVirus (IBD B87) on day Day 14 through water drinking, vaccinated againstwith fowlpox virus (FPV, CVCC AV1003) on day Day 21 through wing puncture, vaccinated againstwith Newcastle disease virus (NDV, La Sota) on day Day 28 through in the water drinking water. The chicks also received preventative treatment for coccidiosis and other parasitic diseases with the application of diclazuril on Days 17-18, sulfaquinoxaline on Days 24-25, and albendazole on Days 31-32. The Coccidiosis and other parasite diseases were also prevented by

Formatted: Font: (Default) Times New Roman, 10.5 pt
Formatted: Font: (Default) Times New Roman
Formatted: Font: (Default) Times New Roman, 10.5 pt
Formatted: Font: (Default) Times New Roman, 10.5 pt
Formatted: Font: (Default) Times New Roman, 10.5 pt
Formatted: Font: (Default) Times New Roman, 10.5 pt
Formatted: Font: (Default) Times New Roman, 10.5 pt
Formatted: Font: (Default) Times New Roman, 10.5 pt
Formatted: Font: (Default) Times New Roman, 10.5 pt
Formatted: Font: (Default) Times New Roman
Formatted: Font: (Default) Times New Roman
Formatted: Font: (Default) Times New Roman
Formatted: Font: (Default) Times New Roman
Formatted: Font: (Default) Times New Roman
Formatted: Font: (Default) Times New Roman
Formatted: Font: (Default) Times New Roman
Formatted: Font: (Default) Times New Roman

applying Diclazuril on day 17-18, Sulfaquinoxaline on day 24-25, and Albendazole on day 31-32,

Body weight records and digesta sample collections

The bBody weight and feed intake of the chickens were recorded for each replicate on day Day 42.,. Thewith an average feed intake of was 3.74 Kg, an the average body weight of was 1.99 Kg, and a the feed conversion ratio of was 1.93, which are consistent with the growth characteristics of this chicken breed. Then, randomly selected chickens were slaughtered on day Day 42, and the intestines were immediately removed and dissected. Fresh digesta samples from the duodenum, jejunum, ileum, cecum, and colorectum were collected and frozen in a dry-ice pack, transported to the laboratory and stored at -80 °C until DNA extraction.

DNA extraction, library preparation, and sequencing

The digesta samples for each intestinal compartment from a total number of 30 chickens were collected for metagenomic DNA extraction. Mainly due to the volume of digesta, it was difficult to process all of the samples at one time. For the convenience of processing, the duodenum digesta from every 5 chickens were pooled together and then washed for microbial cell enrichment and DNA extraction. After processing all duodenum samples, the metagenomic DNA was finally pooled, and further purified with VAHTS DNA Clean Beads (N411-02, Vazyme). The metagenomic DNA samples of the jejunum, ileum, cecum and colorectum were processed in the same way, except that for the cecum, due to its relatively high microbial density, only a subfraction of the pooled and thoroughly mixed digesta was used for microbial cell enrichment and DNA extraction.

The following steps were performed for microbial cell enrichment. The pooled digesta samples were mixed thoroughly with saline buffer containing 0.1% Tween 80 (pre-cooled at 4 °C) by vortexing. The microbial cells were separated through differential centrifugation to remove the undigested feed particles (Huang, et al., 2018) and DNA was extracted from the enriched microbial cells with a DNeasy PowerSoil Pro kit (47014, Qiagen). For the bead beating and lysis options of the DNeasy PowerSoil Pro kit, we added approximately 200 mg of the enriched cells and 800 uL of Solution CD1 into each PowerBead Pro Tube. The tubes were vortexed briefly to mix and incubated at 65 °C for 10 min before the bead beating step. Then, the tubes were placed horizontally and properly balanced on a Vortex Adapter for 24 (1.5–2.0 ml) tubes (QIAGEN, 13000-V1-24) on a Kylin-Bell VORTEX-6. The samples were vortexed in the tubes at maximum speed for 10 min. To ensure the efficiency of the homogenization step, fewer than 12 tubes were vortexed at one time. All the other steps were carried out according to the manufacturer's standard protocol.
The 30 chickens were separated into 6 groups, with each group containing 5 chickens. For each group, the digesta samples for each intestinal compartment were pooled and mixed well with saline buffer containing 0.1% Tween 80 (pre-cooled at 4 °C). The microbial cells were separated through differential centrifugation to remove the undigested feed particles (Huang, et al., 2018) and the enriched microbial cells were used for DNA extraction with DNeasy PowerSoil Pro kit (47014, Qiagen) according to the manufacture's protocol. The metagenomic DNA extracted from 6 groups of the same intestinal compartment were pooled together, and further purified with VAHTS DNA Clean Beads (N411-02, Vazyme). Finally, the combined DNA samples from 30 chicken individuals for duodenum, jejunum, ileum, cecum, and colorectum were generated independently.
The DNA quality and quantity were measured by a Invitrogen Qubit 4 Fluorometer with Qubit™

Formatted: Font: (Default) Times New Roman
Formatted: Font: (Default) Times New Roman
Formatted: Font: (Default) Times New Roman
Formatted: Font: (Default) Times New Roman
Formatted: Font: (Default) Times New Roman
Formatted: Font: (Default) Times New Roman
Formatted: Font: (Default) Times New Roman
Formatted: Font: (Default) Times New Roman
Formatted: Font: (Default) Times New Roman

dsDNA BR (Invitrogen, Q32850) and by a Nanodrop 2000c Microvolume Spectrophotometer. The integrity of the DNA was evaluated on field electrophoresis agarose gels.

The high-integrity genomic DNA was fragmented ~~in~~to 15-20 kb ~~inserts~~ using g-TUBEs (Covaris, USA), and sequencing libraries were prepared by SMRTbell Express Template Prep Kit 2.0 (PacBio, USA). Then, high-fidelity long reads were generated on a PacBio Sequel II ~~with~~ in Circular Consensus Sequence (CCS) mode (PacBio, USA). ~~Based on the fact~~Because microbial diversity ~~is~~ gradually ~~increasing~~ increases from the head to the end point of the intestinal tract, one, two, two, three, and three PacB~~b~~io CCS cells were used for sequencing the duodenum, jejunum, ileum, cecum, and colorectum, respectively.

Metagenome contig assembly and MAG binning

To ensure assembly quality, the raw HiFi sequencing reads were filtered, ~~by~~ requiring read lengths over 2 ~~Kb~~ kb and average read accuracy over 99%. In addition, the remaining reads were ~~also~~ mapped to the host chicken genome ~~as well as~~and feed genomes (maize and soybean) by minimap2 (Minimap2, RRID:SCR_018550) v2-2.20 (Li, 2018) with parameter "-x map-hifi" to remove contaminant sequences, ~~and~~ eliminating approximately~~about~~ 2%, 0.5%, 0.5%, 0.1%, and 0.1% of the reads ~~were filtered as contaminations~~ for the duodenum, jejunum, ileum, cecum, and colorectum samples, respectively. Hifiasm-meta (hifiasm-meta, RRID:SCR_022771) r058 (Feng, et al., 2022) with default parameters was used to assemble the pre-filtered HiFi reads into contigs. By exploiting the contig linkages from the resulting GFA files with Bandage (Bandage, RRID:SCR_022772) v0.8.1 (Wick, et al., 2015), the Hifiasm-meta contigs were divided into 3 classes: (1) circular contig, complete genome assembly of a given species; (2) tangled "circular", many ~~fragmental~~ fragmented contigs linked into a tangled ~~"~~circular genome~~"~~, formed by various heterozygous strains of a species; and (3) linear contig, representing incomplete genome assembly of a species, often due to low coverage depth. T~~Then, t~~he circular contigs were left alone, and each tangled "circular" was independently re-assembled by Hifiasm-meta r058 with default parameters~~independently~~, using these ~~fragmental~~fragmented contigs as input reads. Furthermore, the linear contigs were grouped into metagenome assembled genomes (MAGs) by a binning algorithm MetaBAT2 (MetaBAT, RRID:SCR_019134) v2.12.1 with the parameter "-a depth_file" (Kang, et al., 2019)~~,~~ ~~with~~ using the contig depth obtained from the Hifiasm-meta GFA files. CheckM (CheckM, RRID:SCR_016646) (lineage_wf) v1.1.3 (Parks, et al., 2015) with parameter "lineage_wf" was utilized to evaluate the assembly quality, and 3 quality ranks were adopted: near complete (≥ 90% completeness and < 5% contamination), high~~-~~quality (≥ 70% completeness and < 10% contamination), and medium~~-~~quality (≥ 50% completeness and < 10% contamination).

Construction of non-redundant microbial genome assemblies

~~Limited by computer memory, t~~The sequencing data of each intestinal compartment (duodenum, jejunum, ileum, cecum, and colorectum) was assembled independently, due to the limitations of our computer memory. Then, the microbial genome assemblies (near-complete, high-quality, medium-quality) from all intestinal compartments were put together, and pairwise identity (0~~—~~100) ~~were~~ was calculated by FastANI (FastANI, RRID:SCR_021091) v1.32 (https://github.com/ParBLiSS/FastANI) with default parameters. The identity values were converted into distance values by (100 - identity)/100, and a hierarchical clustering algorithm with

maximum distance was~~ere~~ applied (https://github.com/fanagislab/bioinfo_versatiles/blob/master/hcluster.pl). The stop distances for hierarchical clustering ~~was~~ were set to 0.01 and 0.05 to ~~get~~ obtain strain-level and species-level clusters, respectively. Then, in each cluster, a circular genome was preferred ~~than~~ over a non-circular MAG;~~,~~ in addition, a genome assembly with a larger ~~checkM~~CheckM score (completeness – 5 * contamination) was preferred. After taking the best genome assembly as the representative, the other genome assemblies were taken as redundance and removed. Finally, the non-redundant sets of microbial genomes at the strain-level (ANI 99%) and the species-level (ANI 95%) were generated, respectively.

Taxonomy classification and genome annotation

GTDB-Tk (GTDB-Tk, RRID:SCR_019136) (classify_wf) v1.5.1 (Chaumeil, et al., 2019) with parameter "classify_wf" and its database version r202 ~~was~~ were used for phylogenetic placement and classification of the assembled microbial genomes, and GraPhlAn (GraPhlAn, RRID:SCR_016130) v1.1.3 (Asnicar, et al., 2015) was used for tree visualization. The Ribosomal Database Project (RDP) Classifier (RDP Classifier, RRID:SCR_022773) (http://rdp.cme.msu.edu/classifier/classifier.jsp) V2.11 (Wang, et al., 2007) was used to classify the genome lower taxonomic ranks with 16S rRNA gene sequences, requiring >= 70% confidence. The best hits of the BLAST (BLAST, RRID:SCR_008419) V2.3.1 alignments with the parameters "blastn -task megablast -evalue 1e-5" to the Silva database (r138) (Quast, et al., 2013) were further used to validate the novelty of the taxonomic units. ViralVerify (viralVerify, RRID:SCR_022774) v 1.1 (https://github.com/ablab/viralVerify) with the parameter "--hmm nbc_hmms.hmm" was adopted to classify the assembled genomes into bacteria~~-~~/~~-~~archaea, plasmid and viral genomes. RNAmmer (RNAmmer, RRID:SCR_017075) v1.2 (Lagesen, et al., 2007) with the parameter~~s~~ "-S arc/bac -m lsu,ssu,tsu" was adopted to annotate the 5S, 16S, and 23S rRNA genes, tRNAscan-SE (tRNAscan-SE, RRID:SCR_010835) v2.0.3 (Chan, et al., 2021) with the parameter~~s~~ "-G -H" was adopted to predict tRNA genes, and Prodigal (Prodigal, RRID:SCR_011936) (v2.6.3) (Hyatt, et al., 2010) with parameter "-p single" was used to predict protein-coding genes ~~on~~ from the assembled microbial genomes.

Non-redundant gene catalog construction

Protein-coding gene prediction was performed on the contigs of each intestinal compartment by Prodigal (v2.6.3) (Hyatt, et al., 2010) with the parameter "-p meta". Then, ~~in order~~ to ~~get~~ obtain a non-redundant chicken gut gene catalog at the species-level, the gene models from all the intestinal compartments were put together and redundance were removed by the criteria of identity > 95% and overlap > 90% of the shorter genes, using cd-hit-est (CD-HIT, RRID:SCR_007105) v4.6.6 (Li and Godzik, 2006) with the parameter "-c 0.95 -n 10 -G 0 -aS 0.9". Then, the non-redundant gene catalog was taxonomically annotated using Kaiju (Kaiju, RRID:SCR_022775) v1.9.0 (Menzel, et al., 2016) with the option "-a greedy" based on the NCBI-NR v2020-03-20 database.

To compare the overlap of our gene catalog (HiFi-RGC) with two published chicken gut metagenome gene catalogs (CGM-RGC and GG-IGC) (Feng, et al., 2021; Huang, et al., 2018)~~(Feng, et al., 2021; Huang, et al., 2018)~~, pairwise alignments of HiFi-RGC to CGM-RGC and HiFi-RGC to GG-IGC were performed using BLAT (BLAT, RRID:SCR_011919) (Kent, 2002) with identity ≥

Formatted: Font: (Default) Times New Roman

Formatted: Font: (Default) Times New Roman

Formatted: Font: (Default) Times New Roman

Formatted: Font: (Default) Times New Roman

Formatted: Font color: Auto

95% and overlap ≥ 90% of the shorter genes as the criteria for shared genes.

Acknowledgements

We thank Dan Liu and Wenshu Liu for ~~giving~~ suggestions ~~on the selection of~~ chicken breed selection. We thank Yazhi Qin for assistance ~~helping~~in– raising and slaughtering ~~of~~ the chickens. The work was funded by the National Natural Science Foundation of China (Grant No. 32000408), the Agricultural Science and Technology Innovation Program of CAAS, and fund of Key Laboratory of Shenzhen (ZDSYS20141118170111640).

References

Formatted: Font: (Default) Times New Roman

Asnicar, F., *et al.* Compact graphical representation of phylogenetic data and metadata with GraPhlAn. *PeerJ* 2015;3:e1029.

Bickhart, D.M., *et al.* Generating lineage-resolved, complete metagenome-assembled genomes from complex microbial communities. *Nat Biotechnol* 2022;40(5):711-719.

Chan, P.P., *et al.* tRNAscan-SE 2.0: improved detection and functional classification of transfer RNA genes. *Nucleic Acids Res* 2021;49(16):9077-9096.

Chaumeil, P.A., *et al.* GTDB-Tk: a toolkit to classify genomes with the Genome Taxonomy Database. *Bioinformatics* 2019.

Feng, X., *et al.* Metagenome assembly of high-fidelity long reads with hifiasm-meta. *Nat Methods* 2022.

Feng, Y., *et al.* Metagenome-assembled genomes and gene catalog from the chicken gut microbiome aid in deciphering antibiotic resistomes. *Commun Biol* 2021;4(1):1305.

Gilroy, R., *et al.* Extensive microbial diversity within the chicken gut microbiome revealed by metagenomics and culture. *PeerJ* 2021;9:e10941.

Glendinning, L., *et al.* Assembly of hundreds of novel bacterial genomes from the chicken caecum. *Genome Biol* 2020;21(1):34.

Huang, P., *et al.* The chicken gut metagenome and the modulatory effects of plant-derived benzylisoquinoline alkaloids. *Microbiome* 2018;6(1):211.

Hyatt, D., *et al.* Prodigal: prokaryotic gene recognition and translation initiation site identification. *BMC Bioinformatics* 2010;11:119.

International Chicken Genome Sequencing, C. Sequence and comparative analysis of the chicken genome provide unique perspectives on vertebrate evolution. *Nature* 2004;432(7018):695-716.

Kang, D.D., *et al.* MetaBAT 2: an adaptive binning algorithm for robust and efficient genome reconstruction from metagenome assemblies. *PeerJ* 2019;7:e7359.

Kent, W.J. BLAT--the BLAST-like alignment tool. *Genome Res* 2002;12(4):656-664.

Kolmogorov, M., *et al.* metaFlye: scalable long-read metagenome assembly using repeat graphs. *Nat Methods* 2020;17(11):1103-1110.

Lagesen, K., *et al.* RNAmmer: consistent and rapid annotation of ribosomal RNA genes. *Nucleic Acids Res* 2007;35(9):3100-3108.

Li, H. Minimap2: pairwise alignment for nucleotide sequences. *Bioinformatics* 2018;34(18):3094-3100.

Li, W. and Godzik, A. Cd-hit: a fast program for clustering and comparing large sets of protein or nucleotide sequences. *Bioinformatics* 2006;22(13):1658-1659.

Ludwig, W., *et al.* Bacterial phylogeny based on comparative sequence analysis. *Electrophoresis* 1998;19(4):554-568.

Menzel, P., Ng, K.L. and Krogh, A. Fast and sensitive taxonomic classification for metagenomics with Kaiju. *Nat Commun* 2016;7:11257.

Oakley, B.B., *et al.* The chicken gastrointestinal microbiome. *FEMS Microbiol Lett* 2014;360(2):100-112.

Parks, D.H., *et al.* CheckM: assessing the quality of microbial genomes recovered from isolates, single cells, and metagenomes. *Genome Res* 2015;25(7):1043-1055.

Pellow, D., *et al.* SCAPP: an algorithm for improved plasmid assembly in metagenomes. *Microbiome* 2021;9(1):144.

Quast, C., *et al.* The SILVA ribosomal RNA gene database project: improved data processing and web-based tools. *Nucleic Acids Res* 2013;41(Database issue):D590-596.

Rubin, C.J., *et al.* Whole-genome resequencing reveals loci under selection during chicken domestication. *Nature* 2010;464(7288):587-591.

Sarao, L.K. and Arora, M. Probiotics, prebiotics, and microencapsulation: A review. *Crit Rev Food Sci Nutr* 2017;57(2):344-371.

Segura-Wang, M., *et al.* Genome-Resolved Metagenomics of the Chicken Gut Microbiome. *Front Microbiol* 2021;12:726923.

Sergeant, M.J., *et al.* Extensive microbial and functional diversity within the chicken cecal microbiome. *PLoS One* 2014;9(3):e91941-e91941.

Stackebrandt, E. and Goebel, B.M. Taxonomic note: A place for DNA:DNA reassociation and 16s rRNA sequence analysis in the present spec. In.; 1994.

Sun, Z., *et al.* Expanding the biotechnology potential of lactobacilli through comparative genomics of 213 strains and associated genera. *Nat Commun* 2015;6:8322.

Wang, Q., *et al.* Naive Bayesian classifier for rapid assignment of rRNA sequences into the new bacterial taxonomy. *Appl Environ Microbiol* 2007;73(16):5261-5267.

Wen, C., *et al.* The gut microbiota is largely independent of host genetics in regulating fat deposition in chickens. *The ISME journal* 2019;13(6):1422-1436.

Wick, R.R., *et al.* Bandage: interactive visualization of de novo genome assemblies. *Bioinformatics* 2015;31(20):3350-3352.

Wong, G.K., *et al.* A genetic variation map for chicken with 2.8 million single-nucleotide polymorphisms. *Nature* 2004;432(7018):717-722.

Yeoman, C.J., *et al.* The microbiome of the chicken gastrointestinal tract. *Anim Health Res Rev* 2012;13(1):89-99.

Yue, Y., *et al.* Evaluating metagenomics tools for genome binning with real metagenomic datasets and CAMI datasets. *BMC Bioinformatics* 2020;21(1):334.

Data availability

The HiFi sequencing reads can be found under BioProject ID PRJNA748109: SRR19683891 for duodenum, SRR19732514 and SRR19726169 for jejunum, SRR19736685 for ileum, SRR15214153 and SRR19732730 for cecum, and SRR19683890 and SRR19732729 for colorectum. The assembled contigs, microbial genomes for each intestinal compartments, ~~and~~ non-redundant

Formatted: Font: (Default) Times New Roman

Formatted: Font: (Default) Times New Roman

Formatted: Font: (Default) Times New Roman

Formatted: Font: (Default) Times New Roman

Formatted: Font: (Default) Times New Roman

genome sets at species and strain levels, non-redundant gene catalog, as well asand plasmid and viral annotations are available at AGIS website (ftp://ftp.agis.org.cn/~fanwei/Chicken_gut_metagenome_Hifi/).

Author contributions

Y.Z. and W.F. designed and coordinated the research. Y.Z. and B.Y. prepared the chicken gut materials for sequencing. F.J. and B.Y. performed the data analysis. W.F. wrote the manuscript, and all authors provided suggestions and revised the manuscript.

Ethics approval

This study was approved by the Life Science Ethics Committee of Agricultural Genomics Institute, Chinese Academy of Agricultural Sciences.

Competing interests

The authors declare no competing interests.

Tables

Table 1. Statistics of PacBbio HiFi sequencing data

Intestinal compartment	PacBbio Cell number	Number of reads	Number of bases (bp)	N50 read length (bp)	Median read quality (Phred)
**Duodenum**	1	2,734,871	22,233,516,165	9,778	39
**Jejunum**	2	2,669,321	44,559,115,216	16,417	35
**Ileum**	2	4,282,202	72,828,594,344	16,856	33
**Cecum**	3	5,045,925	80,959,163,166	17,319	31
**Colorectum**	3	5,865,946	111,891,321,947	19,258	31
**All**	11	20,598,265	332,471,710,838	17,316	32

Formatted: Font: (Default) Arial
Formatted Table
Formatted: Font: (Default) Arial
Formatted: Font: (Default) Arial
Formatted: Right
Formatted: Font: (Default) Arial
Formatted: Right
Formatted: Font: (Default) Arial
Formatted: Right
Formatted: Font: (Default) Arial
Formatted: Right
Formatted: Font: (Default) Arial
Formatted: Right
Formatted: Font: (Default) Arial
Formatted: Right

Figures

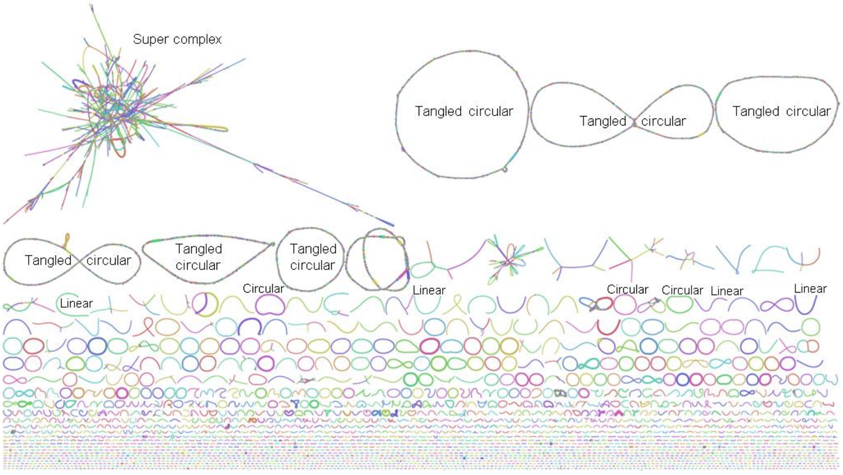

Figure 1. Graphic display of the contig assembly graph. Random colors were chosen for different contigs. The line length is proportional to the~~in proportion to~~ contig length, and the line width is proportional to~~in proportion to~~ the contig coverage depth. Some examples for super complex, tangled circular, individual circular and linear contigs were labeled. This plot ~~is for~~shows the colorectum assembly drawn by Bandage~~, and those for the other intestinal compartments are shown in Figure S1~~.

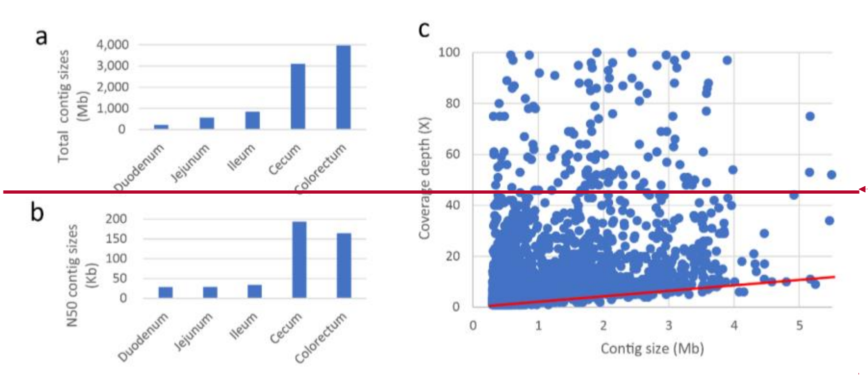

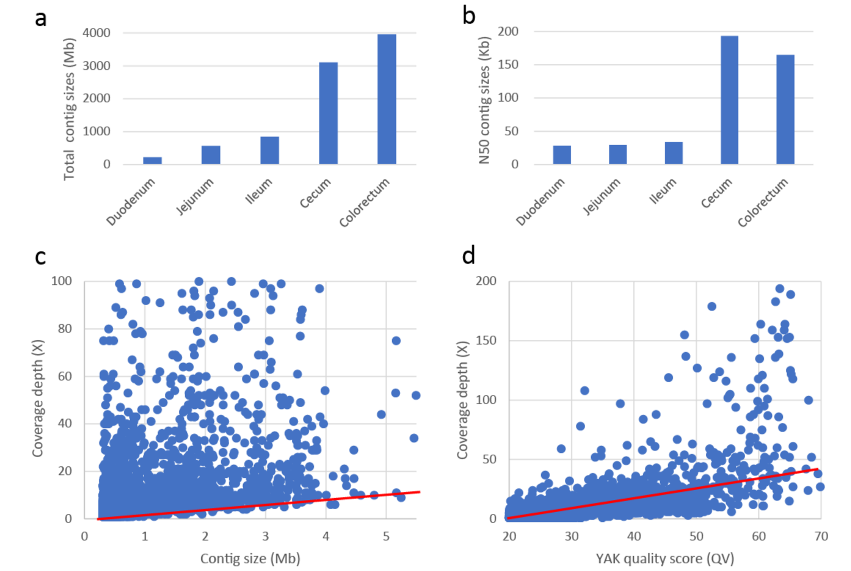

Figure 2. Contig assembly ~~Statistics~~ statistics~~of contig assembly~~. (a) Histogram of total assembled contig sizes for each intestinal compartment. (b) Histogram of N50 contig sizes for each intestinal compartment. (c) Correlation plot of contig length and coverage depth, generated using contig data from all intestinal compartments. The red marker line indicates that sufficient coverage depth contributes to ~~the~~ contig continuity. (d) Correlation plot of the YAK quality score (QV) and coverage depth, using contigs with lengths over 100 kb from all intestinal compartments. The K-mer frequency was calculated with the parameters “yak count -b37 -t48” and the yak QV was calculated with the parameters “yak qv -t80 -p -K3.2g -l100k”. The red marker line indicates that a higher coverage depth improves the single-base quality of the contig sequences.

Formatted: Centered

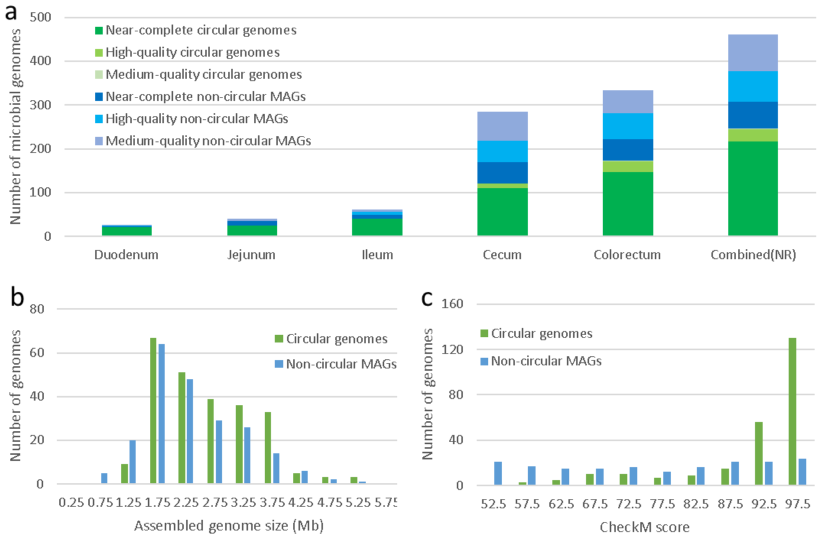

Figure 3. Evaluation and ranking of assembled microbial genomes. (a) “Circular genomes” refers to circular contigs, and “non-circular MAGs” refers to incomplete genome assemblies derived from contig binning or merging algorithms. A circular genome or non-circular MAG is defined as ‘near-complete’~~,~~ if its CheckM completeness is ≥90% and its contamination level ≤5%, defined as ‘high-quality’ if completeness ≥70% and contamination ≤10%, or defined as ‘medium-quality’ if completeness ≥50% and contamination ≤10%. Combined (NR) is the non-redundant set of microbial genomes from all intestinal compartments. All the microbial genomes in Combined (NR) have ≤ 99% ANI to the other microbial genomes in Combined (NR). (b) Distribution of the assembled microbial genome sizes for circular genomes and non-circular MAGs. (c) Distribution of the ~~checkM~~CheckM scores (completeness – 5 * contamination) for circular genomes and non-circular MAGs.

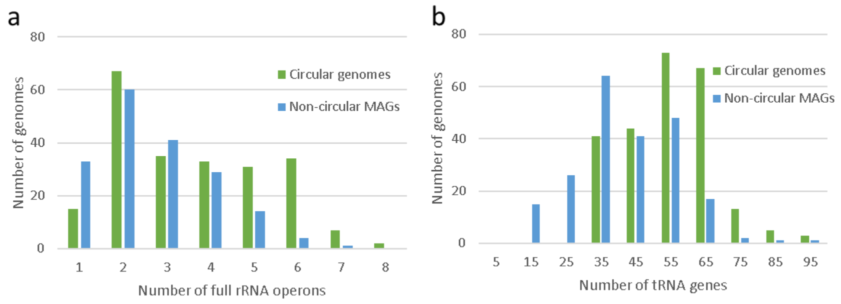

Figure 4. Statistics of non-coding RNA genes in assembled microbial genomes. (a) Distribution of the number of full rRNA operons (i.e., those that encode 5S, 16S, and 23S rRNA)~~, which codes for a 5S, a 16S, and a 23S rRNA~~. (b) Distribution of the number of tRNA genes.

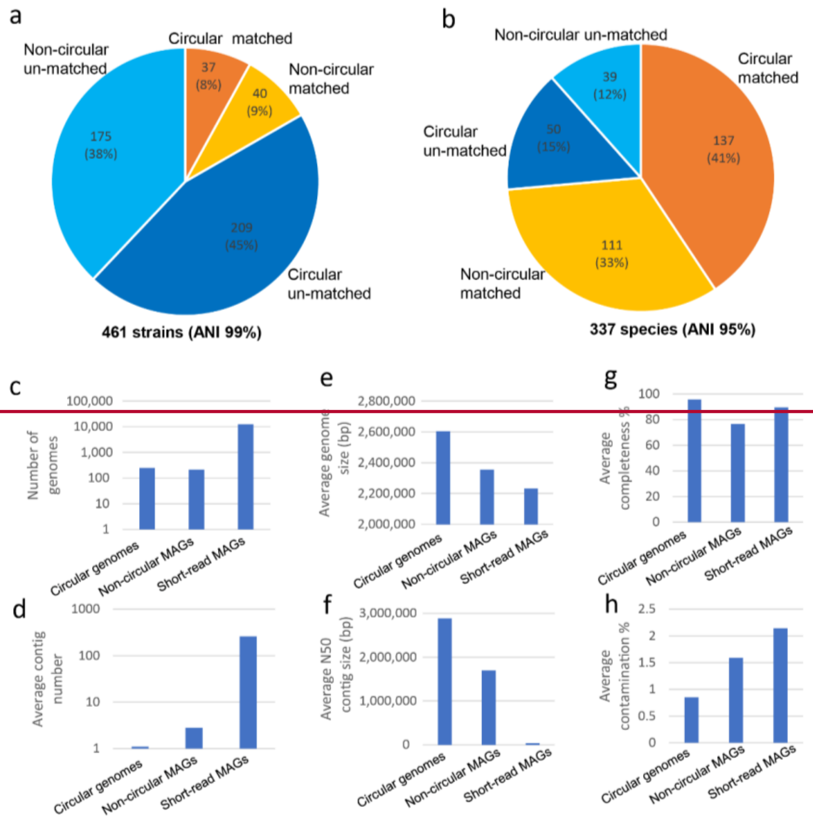
a
Non-circular un-matched
Circular matched
Non-circular matched
Circular un-matched
37 (8%)
40 (9%)
175 (38%)
209 (45%)
461 strains (ANI 99%)
b
Non-circular un-matched
Circular matched
Circular un-matched
Non-circular matched
39 (12%)
50 (15%)
137 (41%)
111 (33%)
337 species (ANI 95%)
c
Number of genomes
e
Average genome size (bp)
g
Average completeness %
d
Average contig number
f
Average N50 contig size (bp)
h
Average contamination %
Circular genomes
Non-circular MAGs
Short-read MAGs

769

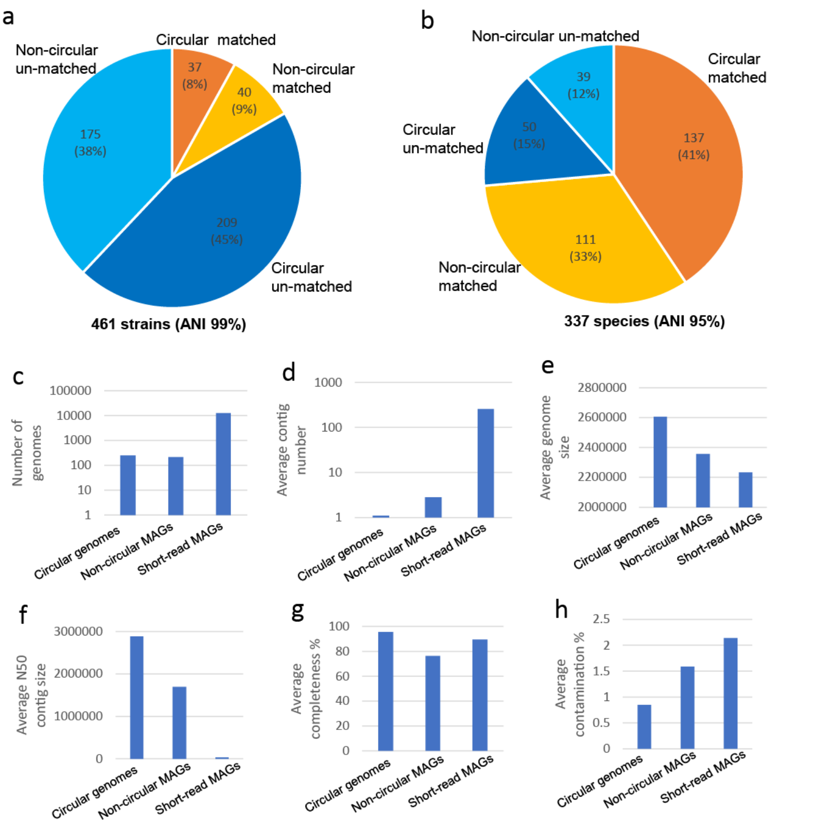

Figure 5. Comparison of HiFi-~~-~~assembled microbial genomes with short-read assembled MAGs. (a) Matching of our 461 assembled microbial strain-level genomes (99% average nucleotide identity, ANI) with ~~public~~ 12,339 public dereplicated MAGs (99% ANI) derived from short-reads. The HiFi-assembled microbial genome was considered a match if its ANI was higher than 99% for any short-read assembled MAG.~~Criterion for matching: HiFi assembled microbial genome has ANI higher than 99% to any short-read assembled MAG.~~ (b) Matching of our 337 assembled microbial species-level genomes (95% ANI) with ~~public~~ 1,978 public dereplicated MAGs (95% ANI) derived from short-reads. The HiFi-assembled microbial genome was considered a match if its ANI was higher than 95% for any short-read assembled MAG~~Criterion for matching: HiFi assembled microbial genome has ANI higher than 95% to any short-read assembled MAG~~. The un-matched microbial genomes unveil candidates of novel strains and species. (c) Number of genomes, (d) average contig number, (e) averaged assembled genome size, (f) average N50 contig size, (g) average ~~checkM~~CheckM completeness, (h) average ~~checkM~~CheckM contamination of the circular genomes, non-circular MAGs and public chicken gut MAGs assembled from short-reads.

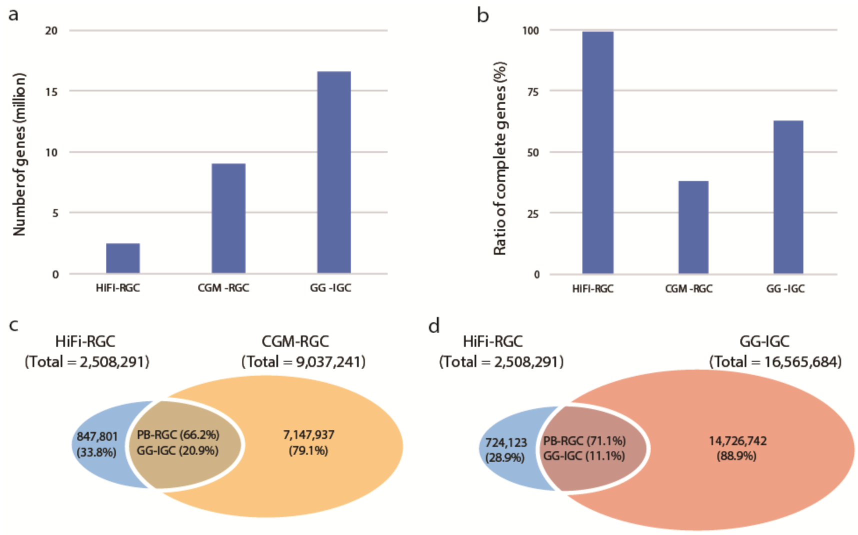

Figure 6. Comparison of <u>the</u> HiFi-derived reference gene catalog (HiFi-RGC) with two published gene catalogs derived from short-read sequencing data (CGM-RGC and GG-IGC). CGM-RGC refer<u>s</u> to chicken gut metagenome – reference gene catalog published by Huang et al. in 2018, GG-IGC refer<u>s</u> to Gallus gallus – Integrated gene catalog published by Feng et al. in 2021. (a) Gene number and (b) gene structure completeness ratio of the 3 gene catalogs. Overlap of HiFi-RGC and CGM-RGC (c) and GG-IGC (d). A confident ~~share~~ <u>overlap</u> is defined by the criteria of sequence identity ≥95% and length overlap ≥90% of the shorter sequence.

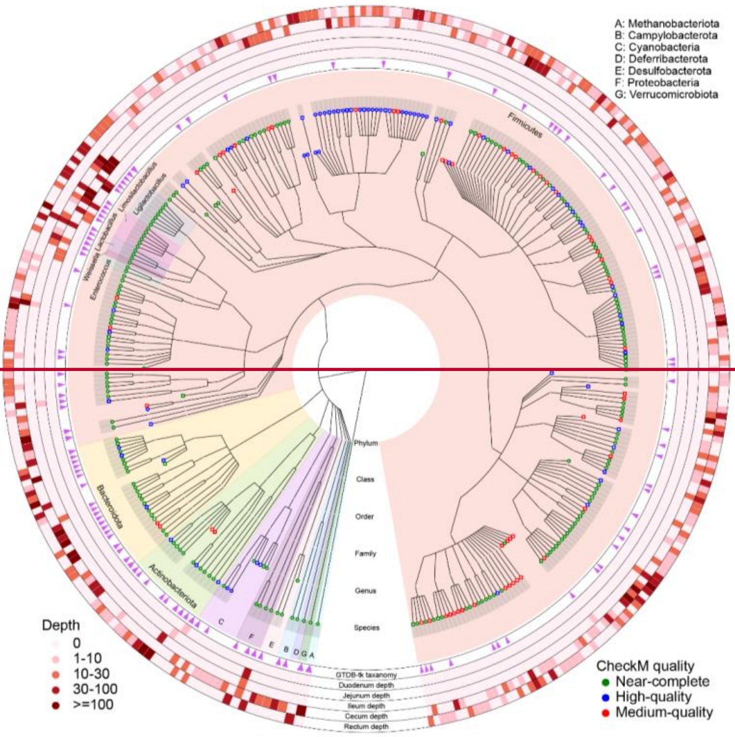
A: Methanobacteriota
B: Campylobacterota
C: Cyanobacteria
D: Deferribacterota
E: Desulfobacterota
F: Proteobacteria
G: Verrucomicrobiota
Firmicutes
Bacteroidota
Actinobacteriota
Phylum
Class
Order
Family
Genus
Species
GTDB-tk taxanomy
Duodenum depth
Jejunum depth
Ileum depth
Cecum depth
Rectum depth
Depth
0
1-10
10-30
30-100
>=100
CheckM quality
Near-complete
High-quality
Medium-quality

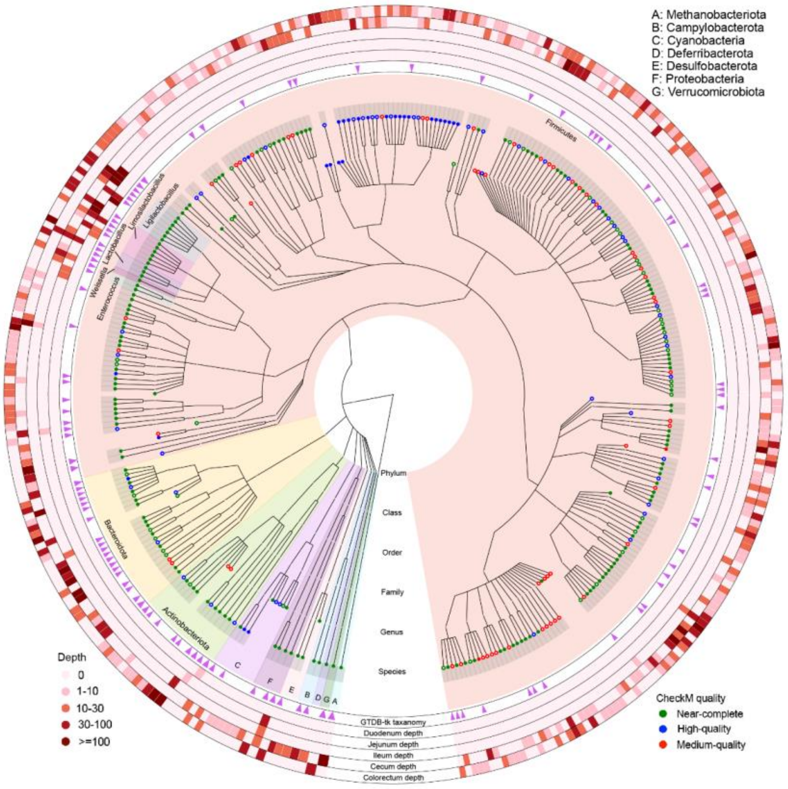

Figure 7. Phylogeny of the HiFi-assembled microbial genomes. ~~A~~ Each colored clade corresponds to a phylum inferred by GTDB-Tk. Inside the largest phylum Firmicutes, five ~~genus~~ genera *Ligilactobacillus*, *Limosilactobacillus*, *Lactobacillus*, *Weissella*, and *Enterococcus* are also colored for highlighting. The leaf nodes of the phylogenetic tree have two shapes: a "solid circle" represents a circular genome, and a "hollow circle~~rectangle~~" represents a non-circular MAG. The colors of the leaf nodes represent ~~checkM~~CheckM quality ranks: "green" represents~~refers to~~ N~~n~~ear-complete assemblies, "blue" represents~~refers to~~ "High-quality assemblies", and "red" represents~~refers to~~ "Medium-quality assemblies". The inner ring shows the ~~GTDB-tk~~GTDB-Tk classification, and a triangle ~~means~~ indicates that the corresponding leaf node ~~is matched to~~matches an existing genome in the GTDB database. The five outer rings show the sequencing coverage depth for each assembled microbial genome from each intestinal compartment~~, respectively~~. From inner to outer: duodenum, jejunum, ileum, cecum, and colorectum.

Formatted: Font: 10.5 pt
Formatted: Font: 10.5 pt
Formatted: Font: 10.5 pt
Formatted: Font: 10.5 pt
Formatted: Font: 10.5 pt
Formatted: Font: 10.5 pt, Not Highlight
Formatted: Font: 10.5 pt
Formatted: Font: 10.5 pt
Formatted: Font: 10.5 pt
Formatted: Font: 10.5 pt, Not Highlight
Formatted: Font: 10.5 pt
Formatted: Font: 10.5 pt
Formatted: Font: 10.5 pt
Formatted: Font: 10.5 pt
Formatted: Font: 10.5 pt
Formatted: Font: 10.5 pt
Formatted: Font: 10.5 pt
Formatted: Font: 10.5 pt
Formatted: Font: 10.5 pt
Formatted: Font: 10.5 pt
Formatted: Font: 10.5 pt
Formatted: Font: 10.5 pt
Formatted: Font: 10.5 pt
Formatted: Font: 10.5 pt

Click here to access/download
Supplementary Material
Supplemental_Revison1_20220920.docx

 Click here to access/download;Personal Cover;Response to reviewers.docx

Dear Dr Fan,

Your manuscript "Improved microbial genomes and gene catalog of chicken gut from metagenomic sequencing of high-fidelity long reads" (GIGA-D-22-00175) has been assessed by our reviewers. Although it is of interest, we are unable to consider it for publication in its current form. The reviewers have raised a number of points which we believe would improve the manuscript and may allow a revised version to be published in GigaScience.

Their reports, together with any other comments, are below. Please also take a moment to check our website at https://www.editorialmanager.com/giga/ for any additional comments that were saved as attachments.

In addition, please register any new software application in the bio.tools and SciCrunch.org databases to receive RRID (Research Resource Identification Initiative ID) and biotoolsID identifiers, and include these in your manuscript. This will facilitate tracking, reproducibility and re-use of your tool.

Reply: we have registered new software in the SciCrunch.org databases, and included RRIDs of all the used software in our manuscript.

If you are able to fully address these points, we would encourage you to submit a revised manuscript to GigaScience. Once you have made the necessary corrections, please submit online at:

https://www.editorialmanager.com/giga/

If you have forgotten your username or password please use the "Send Login Details" link to get your login information. For security reasons, your password will be reset.

Please include a point-by-point within the 'Response to Reviewers' box in the submission system. Please ensure you describe additional experiments that were carried out and include a detailed rebuttal of any criticisms or requested revisions that you disagreed with. Please also ensure that your revised manuscript conforms to the journal style, which can be found in the Instructions for Authors on the journal homepage. If the data and code has been modified in the revision process please be sure to update the public versions of this too.

Reply: We have addressed all the questions raised by the reviewers, and submitted a revised manuscript and a point-by-point response letter for your consideration.

The due date for submitting the revised version of your article is 11 Dec 2022.

I look forward to receiving your revised manuscript soon.

Best wishes,

Hongling Zhou
GigaScience
www.gigasciencejournal.com

Reviewer reports:

Reviewer #1: The authors sequenced the chicken gut microbiome using HiFi sequencing based on PacBio Sequel II platform, and further revealed its advantages compared with traditional short-read sequencing: higher quality of assemblies for gene structures and more recovered novel genes and genomes. Although the conclusions obtained in this study are right, this work meets our expectations and is a commonsense. In addition, this study is a little bit simple because they only compare the two sequencing technologies. The authors should perform some analysis about scientific findings in chicken gut microbiome. The detailed comments are as following：

1. This paper has spent large effort to prove the advantage of the high-fidelity (HiFi) sequencing, but a lot of previous research have reported the advantage of the high-fidelity (HiFi) sequencing and it is a commonsense. What is really new in this paper? The authors should reveal the scientific findings among different chicken-gut microbiota.

Reply: Thanks to the reviewer's suggestions. As this is the first HiFi metagenome project for chicken, we have performed detailed comparisons of the HiFi metagenome data and short-read metagenome data. Although the advantage of HiFi metagenome sequencing has been shown by a few previous studies, our analysis has used different assembly method, so we think it is necessary to make detailed comparisons again, which can assure that the chicken HiFi-derived microbial genomes and gene catalogue are both in high quality.

We admit that the experiment design of this study is a bit simple. However, we think that the quick release of the generated resources, including the assembled novel strain and species genomes, as well as the predicted novel genes, will be very helpful to the scientific world and chicken production industry. We have also made several scientific findings from our metagenome data analysis, such as: (1) The dominant phyla in our HiFi assembled genomes is Firmicutes (82.5%), and the foregut is highly enriched in five genera *Ligilactobacillus*, *Limosilactobacillus*, *Lactobacillus*, *Weissella*, and *Enterococcus*. (2) Among the 337 species-level microbial genomes, 9 and 49 of them may belong to novel genera and species, respectively.

Now we have added some more analysis and descriptions in the manuscript, and the original Result section "Phylogeny of HiFi assembled microbial genomes and novel genomic representation"was split into two sections "Phylogeny of HiFi assembled microbial genomes and differences among intestinal compartments" and "Novel genomic representation and novel genus and species discovery". Some important revisions were listed below:

(1) In Result section “Phylogeny of HiFi assembled microbial genomes and differences among intestinal compartments”, we have added:

“The remaining genomes were classified as Cyanobacteria, Proteobacteria, Desulfobacterota, Campylobacterota, Deferribacterota, Methanobacteriota, and Verrucomicrobiota.”;

“The foregut contains the duodenum, jejunum, and ileum, which mainly function in feed digestion and nutrient absorption. The hindgut contains the cecum and colorectum, which function in fermentation, detoxification and recycling of residual water and salt.”;

(2) In Result section “Novel genomic representation and novel genus and species discovery”, we have added:

“Some of these new genomic sequences may have potential benefits to industry or medical applications. Lactobacillus has traditionally been used in the fermentation industry, producing lactate from raw carbohydrates and synthetic media (Sun, et al., 2015). In recent years, Lactobacillus and its close relatives Ligilactobacillus and Limosilactobacillus have also been widely adopted as probiotic supplements, either in animal feed to promote growth or human foods to improve human health (Sarao and Arora, 2017). Among our 337 assembled microbial species genomes, 3 genomes belong to Lactobacillus, 7 genomes belong to Ligilactobacillus, and 6 genomes belong to Limosilactobacillus. All these genomes have been successfully classified to the genus level, and most of the genomes were successfully classified to the species level by GTDB-Tk. However, 2 Ligilactobacillus genomes and 1 Limosilactobacillus genome have not been classified to the species level, suggesting that these 3 species-level genomes may represent novel genomic resources for probiotic development.”;

“In addition, approximately one-third of these newly discovered genera and half of these newly discovered species were not found in the short-read MAG data, suggesting that they are derived only from HiFi metagenome data, which further shows the advantage of HiFi sequencing in metagenomic studies.”;

(3) In Result section “Advantage of HiFi-derived gene catalog over gene catalogs from short-reads”, we have added a paragraph:

“By comparing the pairwise overlap at the gene sequence level, we found that 847,801 (33.8%) and 724,123 (28.9%) genes are unique in HiFi-RGC compared to CGM-RGC and GG-IGC, respectively (Figure 6c-d), suggesting that the HiFi-derived gene catalog recovered a substantial portion of the genes that were missed by short-read technologies. Because GG-IGC is more comprehensive than CGM-RGC, we considered the 724,123 (28.9%) genes in HiFi-RGC as unique genes and the remaining genes (71.1%) in HiFi-RGC as shared genes. Then, the microbial communities derived from the unique and shared genes in HiFi-RGC were compared. The results showed that 36.8% of unique genes were unclassified at the phylum level, which was obviously higher than the proportion of shared genes (24.9%), suggesting that the unique genes are enriched in unknown phyla (Figure S6).”

2. Method section: "The 30 chickens were separated into 6 groups, with each group containing 5 chickens.". However, the digesta samples of the 30 chickens were finally pooled, mixed together and analyzed based on the difference of intestinal compartment. Pls elucidate what the meaning of classification is?

Reply: The mentioned “classification” was just for the convenience of experimental processing, as the total volume of digesta from all chickens was large and difficult to process in one time. For each intestinal compartment， samples from all the 30 chickens were pooled altogether in two steps of pooling (digesta pooling and extracted DNA pooling). The manuscript has been revised with more detailed and clear description：

“Mainly due to the volume of digesta, it was difficult to process all of the samples at one time. For the convenience of processing, the duodenum digesta from every 5 chickens were pooled together and then washed for microbial cell enrichment and DNA extraction. After processing all duodenum samples, the metagenomic DNA was finally pooled, and further purified with VAHTS DNA Clean Beads (N411-02, Vazyme). The metagenomic DNA samples of the jejunum, ileum, cecum and colorectum were processed in the same way, except that for the cecum, due to its relatively high microbial density, only a subfraction of the pooled and thoroughly mixed digesta was used for microbial cell enrichment and DNA extraction.”

3. The line number should be added in the manuscript, and English needs to be polished throughout the MS.

Reply: We have added the line number and polished the English by a native speaker.

Reviewer #2: The authors present work that expands the databases of chicken microbiome genomes with high quality assemblies and represents a valuable resource. The authors also demonstrate a step towards moving from short read assembly of metagenomes towards long read assemblies, which is undoubtedly the direction the field should be moving in.

While I expect these genomes to be high quality and not heavily impacted by indels given the accuracy of hifi and the high completeness statistics, a common criticism of long-reads is the error rate. Have the authors considered demonstrating that this is not impacting their high quality genomes through, for example, the ideel tool or some other assessment of error rate in the assemblies? This may strengthen the message that long reads are suitable for this kind of work, particularly if the result is independent of the depth of coverage over each species.

Reply: Before metagenome assembly, we have pre-filtered low quality HiFi reads, and only HiFi reads with quality over Q20 were used for metagenome assembly. The high base quality of input reads makes sure that the high base quality of the contig assembly.

In respect to the hifiasm-meta algorithm, it first performs HiFi-reads error correction from overlapped reads, which is equivalent to call the consensus sequences from multiple aligned reads. So, higher reads coverage will improve the error correction of the HiFi reads, and finally improve the base accuracy of the contig assemblies. In the hifiasm-meta paper (Feng, X., et al. Metagenome assembly of high-fidelity long reads with hifiasm-meta. Nat Methods 2022), Extended Data Fig.2 shows Yak quality value (QV) score correlated with contig coverage, and higher coverage will result in higher Yak QV. Yak QV is a rough estimation of the single base accuracy, including both mismatch and indel errors.

Here, we performed the same analysis using our chicken metagenome contig data, and obtained a similar result, which was shown in Figure 2d: Correlation plot of YAK quality score (QV) and coverage depth, using contigs with length over 100 Kb from all intestinal compartments. The K-mer frequency was calculated with parameters "yak count -b37 -t48" and yak QV was calculated with parameters "yak qv -t80 -p -K3.2g -l100k". The red marker line indicates that higher coverage depth will improve the single base quality of the contig sequences.

We have also added descriptions in the maintext (Results/Longer contigs of the chicken metagenome assembled from high-fidelity long reads): "Moreover, the coverage depth is positively related with the single-base quality values, indicating that higher coverage depth will improve the single-base accuracy of the contig sequences (Figure 2d).".

In addition, we also used the checkM completeness to evaluate the single base accuracy of the assembled genomes: We added a supplementary figure S5: Correlation plot of genome coverage depth and checkM completeness score. The 187 circular genomes out of 337 non-redundant species-level genomes were used here. Considering all these genomes have complete genome assemblies, the difference of checkM completeness scores should only be caused by the single base accuracy, due to the marker gene prediction method adopted by checkM. Genome assemblies with higher single base accuracy will have higher checkM completeness values.The plots clearly shows that higher coverage depth will result in higher checkM completeness scores, indicating that higher coverage depth will improve the single base accuracy of genome assemblies.

We also added descriptions in the maintext "Using the 187 circular species-level genomes, which all have complete genome assemblies, we showed that higher coverage depth is positively correlated with CheckM completeness score, indicating that a higher coverage depth will improve the single-base accuracy of the genome assemblies (Figure S5)."

Can the authors elaborate on the methods they used to compare the existing gene catalogs to their own as this appears to not be described in the methods section?

Reply: We have added the methods for comparing the gene catalogs in Methods/ Non-redundant gene catalog construction:

To compare the overlap of our gene catalog (HiFi-RGC) with two published chicken gut metagenome gene catalogs (CGM-RGC and GG-IGC) (Feng, et al., 2021; Huang, et al., 2018), pairwise alignments of HiFi-RGC to CGM-RGC and HiFi-RGC to GG-IGC were performed using BLAT (BLAT, RRID:SCR_011919) (Kent, 2002) with identity ≥ 95% and overlap ≥ 90% of the shorter genes as the criteria for shared genes.

Can the authors include in the supplement some stats on the quantity and quality of DNA extracted from each sample type? It is very useful for people considering using the same protocol especially when long-read sequencing typically requires high levels of high quality starting material.

Reply: We have added the quantity and quality information in Table S1 and Figure S1. The DNA quality and quantity were measured by Invitrogen Qubit 4 Fluorometer with Qubit™ dsDNA BR(Invitrogen, Q32850) and by Nanodrop 2000c Microvolume Spectrophotometer (Table S1). The integrity of DNA was evaluated on field electrophoresis agarose gels (Figure S1).

Table S1. Quality and Quantity assessment of the extracted DNA

**Metagenomic DNA samples**	**Volume (µl)**	**Qubit 4**		**Nanodrop 2000c**			
		**Quantity (ug)**	**Concentration (ng/µl)**	**Quantity (ug)**	**Concentration (ng/µl)**	**A260/A280**	**A260/A230**
Duodenum	60	3.66	61	4.62	77	1.91	2.19
Jejunum	120	13.68	114	16.92	141	1.86	2.04
Ileum	120	27.36	228	31.68	264	1.86	2.21
Cecum	120	83.52	696	84.72	706	1.87	2.18
Colorectum	120	56.16	468	70.80	590	1.87	2.15

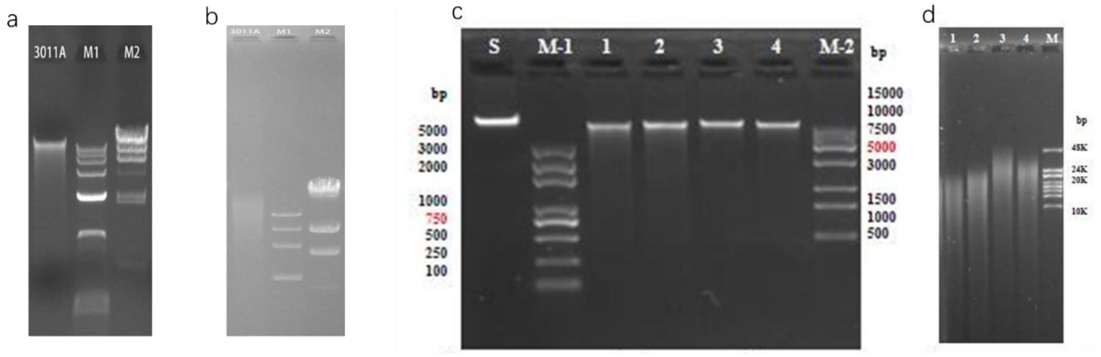

Figure S1. Agarose gel electrophoresis. (a) DC electrophoresis (0.7% gel, 100V, 1h) for Duodenum microbiota DNA (3011A); M1 15kb DNA Marker(15000、10000、7500、5000、2500、1000、250bp); M2 λDNA /HindIII(23130、9416、6557、4361、2322、2027、564bp). (b) Pulse electrophoresis (0.7% gel, pulse 5~80kb, 16h) for Duodenum microbiota DNA (3011A); M1 15kb DNA Marker; M2 λDNA /HindIII; (c) DC electrophoresis (1% gel, 180V, 20min) for Jejunum microbiota DNA (lane 1), Ileum microbiota DNA (lane 2), Cecum microbiota DNA (lane 3), and Colorectum microbiota DNA (lane 4); S stantard sample (50ng); M-1 trans 2k plus; M-2 trans 15k plus. (d) Pulse electrophoresis (0.8% gel, pulse 5~80kb, 17h) for Jejunum microbiota DNA (lane 1), Ileum microbiota DNA (lane 2), Cecum microbiota DNA (lane 3), and Colorectum microbiota DNA (lane 4); M 48kb DNA Extension Ladder. In summary, the microbiota DNA from all intestinal fragments are intact except for the Duodenum, which is slightly degraded. The microbiota DNA from all intestinal fragments are qualified for HiFi sequencing.

The authors state that computational limitations prevented them from doing a co-assembly, but did they include coverage data from multiple gut regions while binning their non-circular contigs with metabat2? Using many samples from different individuals and regions of the gut aligned to the contigs you are binning can improve the outcome from binning and uses more data without requiring the extreme computational power of the assembly stage.

Reply: Thanks for the reviewer's suggestion. We agreed that using many samples from different individuals and regions of the gut may improve the binning process. If we have taken a co-assembly of all the intestinal data and generated one contig data set, then that will be a certain choice.

However, a big limitation in metagenome assembly is the computer memory, for example, only the assembly of cecum data needs about 800 G memory, and it will take over 3000 G memory for a co-assembly of all the 5 intestinal fragments. Considering that the maximum memory of our computer server is 1000 G, we assembled each of the 5 intestinal fragments separately, resulting in 5 hifiasm-meta contig data sets. Then, a major task is to merge them into one assembly result. If

we merge all the contigs firstly, then the linkage and depth information from the hifiasm-meta GFA files will be lost.

To fully utilize the linkage and depth information from the hifiasm-meta GFA file, we decided to get genome-level assembly for each of the 5 intestinal fragment firstly, including 3 parts: (1) the circular contigs were taken as complete genome; (2) “tangled”circular were re-assembled into non-redundant contig assembly; (3) linear contig were binned by Metabat2 using the depth information obtained from the hifiasm-meta GFA file of the corresponding intestinal fragment. In this way, the contigs and the depth information matched well. Finally, the genome-level assemblies from all the 5 intestinal fragments were merged into a non-redundant genome-level assembly. We think this strategy is more suitable for our data analysis. Although the results here may not be the best, but it should be reliable.

There are a couple of instances of GTDB-tk being written as GTDT-tk, please double check this is correct throughout.

Reply: We have corrected all “GTDT-tk”to “GTDB-tk”.

I find the panel order in figure 5 to be unintuitive, I suggest that the panels below A and B go in order from left to right rather than top to bottom.

Reply: We have re-ordered the panel order of figure 5, let c, d, e in one row, and f, g, h in another row.

The last paragraph of page 7 is a little fiddly to follow, it might be better presented as a table or just referring the reader to see the data in the figure.

Reply: We have added a supplementary Table (Table S5) to make it easy to follow:

Table S5. Unclassified number of genomes at each taxonomic level

	**GTDB-tk**	**GTDB-tk + RDP**	**GTDB-tk + RDP + Silva**
**Family level**	2	1	0
**Genus level**	35	21	9
**Species level**	189	189	49

Note: Here the 337 species-level microbial genomes were used as input, and the unclassified number of genomes at each taxonomic levels (family, genus, species) were shown by three classification methods: (1) GTDB-tk alone; (2) GTDB-tk and Ribosomal Database Project (RDP) Classifier; (3) GTDB-tk and Ribosomal Database Project (RDP) Classifier and alignments to the Silva 16S rRNA database. The last method classified the maximum number of genomes, and only 9 and 49 genomes

failed to be classified into known genus and species, indicating that they may be novel genus and species which haven’t been reported before.

Page 7 refers to "figure c-d" without a figure number.

Reply: "figure c-d" has been changed to “"Figure 6c-d"”.

I like figure 7, but a couple of parts are difficult to interpret. The circles and squares are not very distinct, is it possible for the squares to be another shape that is less similar to the circles? With the aid of zooming in it is possible to read the labels on the depth tracks, but for convenience I would recommend also listing the labels in order in the caption.

Reply: We have used hollow circles to replace the squares, and used solid circles to replace the circles. We also listed the labels in order in the caption “From inner to outer: duodenum, jejunum, ileum, cecum, and colorectum.”.

I find this sentence on page 10 a bit ambiguous and suggest adjusting for clarification "Finally, the combined DNA samples from 30 chicken individuals for duodenum, jejunum, ileum, cecum, and colorectum were generated independently." (i.e. what is combined and what is independent)

Reply: For each intestinal compartment, all the extracted DNA from all chickens were combined. The digesta samples from different intestinal compartment were processed separately. Finally，there were only 5 metagenomic DNA samples of duodenum, jejunum, ileum, cecum, and colorectum. The manuscript was revised for a better clarification：

“Mainly due to the volume of digesta, it was difficult to process all of the samples at one time. For the convenience of processing, the duodenum digesta from every 5 chickens were pooled together and then washed for microbial cell enrichment and DNA extraction. After processing all duodenum samples, the metagenomic DNA was finally pooled, and further purified with VAHTS DNA Clean Beads (N411-02, Vazyme). The metagenomic DNA samples of the jejunum, ileum, cecum and colorectum were processed in the same way, except that for the cecum, due to its relatively high microbial density, only a subfraction of the pooled and thoroughly mixed digesta was used for microbial cell enrichment and DNA extraction.”

Can the authors clarify in the methods section the method of bead beating used in the protocol, for reproducibility.

Reply: The details about bead beating were added into the manuscript："For the bead beating and lysis options of the DNeasy PowerSoil Pro kit, we added approximately 200 mg of the enriched cells and 800 uL of Solution CD1 into each PowerBead Pro Tube. The tubes were vortexed briefly to mix and incubated at 65 °C for 10 min before the bead beating step. Then, the tubes were placed horizontally and properly balanced on a Vortex Adapter for 24 (1.5–2.0 ml) tubes (QIAGEN, 13000-V1-24) on a Kylin-Bell VORTEX-6. The samples were vortexed in the tubes at maximum speed for 10 min. To ensure the efficiency of the homogenization step, fewer than 12 tubes were vortexed at one time. All the other steps were carried out according to the manufacturer's standard protocol."

--

Please also take a moment to check our website at https://www.editorialmanager.com/giga/l.asp?i=118960&l=WV0ET124 for any additional comments that were saved as attachments. Please note that as GigaScience has a policy of open peer review, you will be able to see the names of the reviewers.

In compliance with data protection regulations, you may request that we remove your personal registration details at any time. (Use the following URL:https://www.editorialmanager.com/giga/login.asp?a=r). Please contact the publication office if you have any questions.
