# Supplementary material for: Improved microbial genomes and gene catalog of the chicken gut from metagenomic sequencing of high-fidelity long reads
Source: Gigascience. 2022 Nov 18;11:giac116. doi: 10.1093/gigascience/giac116 (PMC9673493; doi:10.1093/gigascience/giac116)
Supplement: giac116_GIGA-D-22-00175_Revision_2 [file giac116_giga-d-22-00175_revision_2.pdf]

GigaScience

Improved microbial genomes and gene catalog of chicken gut from metagenomic sequencing of high-fidelity long reads

--Manuscript Draft--

**Manuscript Number:**	GIGA-D-22-00175R2
**Full Title:**	Improved microbial genomes and gene catalog of chicken gut from metagenomic sequencing of high-fidelity long reads
**Article Type:**	Research
**Funding Information:**	National Natural Science Foundation of China (Grant No. 32000408) — Dr. Yan Zhang
**Abstract:**	Background<br><br>Due to the importance of chicken production and the remarkable influence of gut microbiota on the host's health and growth, tens of thousands of metagenome-assembled genomes (MAGs) have been constructed for the chicken gut microbiome. However, limited by the short-read sequencing and assembly technologies, most of these MAGs are far from complete and have lower qualities with contamination.<br><br>Results<br><br>We generated 332 Gb high-fidelity (HiFi) long reads from the five chicken intestinal compartments, and assembled 461 and 337 microbial genomes at species and strain level, of which 53% and 55% are circular genomes, respectively. For the assembled microbial genomes, about 95% were regarded as complete according to the "RNA complete" criteria, which requires at least one full-length rRNA operon coding for all three types of rRNA(16S, 23S and 5S rRNA) and at least 18 copies of full-length tRNA genes. In comparison with the short-read derived chicken MAGs, 384 (83% of 461) and 89 (26% of 337) strain-level and species-level genomes in this study are novel and do not have any matches. At the gene level, a third of the 2.5 million genes in HiFi derived gene catalog are novel and cannot be matched to the short-read derived gene catalogs. Moreover, the HiFi derived genomes have much higher continuity and completeness, as well as lower contamination; the HiFi derived gene catalog has much higher ratio of complete gene structures. The dominant phyla in our HiFi assembled genomes is Firmicutes (82.5%), and the foregut is highly enriched in five genera Ligilactobacillus , Limosilactobacillus , Lactobacillus , Weissella , and Enterococcus , all of which belongs to the order Lactobacillales. Using GTDB-tk, all the 337 species-level genomes were successfully classified to order level, however, 2, 35 and 189 genomes could not be classified into any known family, genus, and species, respectively. Among these not-fully classified genomes, 9 and 49 of them may belong to novel genera and species, respectively, for that their 16S-rRNA genes have identities lower than 95% and 97% to any known 16S-rRNA genes.<br><br>Conclusions<br><br>HiFi sequencing not only remarkably improved the quality of metagenome assemblies and gene structures, but also recovered a substantial portion of novel genomes and genes that were missed in short-read metagenome studies. The novel genomes or novel species obtained in this study will facilitate the gut microbiome and host-microbiota interaction studies, which would benefit the sustainable development of poultry.
**Corresponding Author:**	Wei Fan<br>Chinese Academy of Agricultural Sciences<br>shenzhen, guangdong CHINA
**Corresponding Author Secondary Information:**	
**Corresponding Author's Institution:**	Chinese Academy of Agricultural Sciences

Powered by Editorial Manager® and ProduXion Manager® from Aries Systems Corporation

**Corresponding Author's Secondary Institution:**	
**First Author:**	Wei Fan
**First Author Secondary Information:**	
**Order of Authors:**	Wei Fan
	Yan Zhang
	Fan Jiang
	Boyuan Yang
	Sen Wang
	Hengchao Wang
	Anqi Wang
	Dong Xu
**Order of Authors Secondary Information:**	
**Response to Reviewers:**	I've made some revisions and added some comments in your manuscript, please check all changes and resolve all my comments.<br>Reply: Thanks for the revisions of our manuscript. We have checked all the changes and reformatted the references and URLs to the GigaScience style. We also searched the two questioned RRIDs, RRID:SCR_022773 and RRID:SCR_022774, and found they both can be found in the RRID database （https://www.rrids.org/）, but were displayed at the second item. The searching results are listed below:<br><br>RRID:SCR_022773<br>https://sourceforge.net/projects/rdp-classifier/<br>Software tool as naive Bayesian classifier that can rapidly and accurately provide taxonomic assignments from domain to genus, with confidence estimates for each assignment.<br>Proper citation: RDP Classifier (RRID:SCR_022773)<br><br>RRID:SCR_022774<br>https://github.com/ablab/viralVerify<br>Software viral contig verification tool. Classifies contigs as viral, non-viral or uncertain, based on gene content. Also for non-viral contigs it can optionally provide plasmid/non-plasmid classification.<br>Proper citation: viralVerify (RRID:SCR_022774)<br><br><br>Also please meet the following requirements:<br>1.Figures should be provided as separate files, not embedded in the main manuscript file.<br>2.Each figure of a manuscript should be submitted as a single file that fits on a single page in portrait format.<br>3.Figure titles (max 15 words) and legends (max 300 words) should be provided in the main manuscript, not in the graphic file.<br>Reply: Each figure has been submitted as single file, and the figure titles and legends meet the requirements.<br><br>4.All references, including URLs, must be numbered consecutively, in square brackets, in the order in which they are cited in the text, followed by any in tables or legends.<br>Reply: We have reformatted all the reference papers and URLs.<br><br><br><br>Reviewer reports:<br>Reviewer #1: The revised manuscript has addressed all my concerns except a new nomenclature table for all abbreviations.<br>Reply: We have provided a nomenclature list for all abbreviations.

Powered by Editorial Manager® and ProduXion Manager® from Aries Systems Corporation

Additional Information:	
Question	Response
Are you submitting this manuscript to a special series or article collection?	No
**Experimental design and statistics**<br><br>Full details of the experimental design and statistical methods used should be given in the Methods section, as detailed in our Minimum Standards Reporting Checklist. Information essential to interpreting the data presented should be made available in the figure legends.<br><br>Have you included all the information requested in your manuscript?	Yes
**Resources**<br><br>A description of all resources used, including antibodies, cell lines, animals and software tools, with enough information to allow them to be uniquely identified, should be included in the Methods section. Authors are strongly encouraged to cite Research Resource Identifiers (RRIDs) for antibodies, model organisms and tools, where possible.<br><br>Have you included the information requested as detailed in our Minimum Standards Reporting Checklist?	Yes
**Availability of data and materials**<br><br>All datasets and code on which the conclusions of the paper rely must be either included in your submission or deposited in publicly available repositories (where available and ethically appropriate), referencing such data using a unique identifier in the references and in the "Availability of Data and Materials" section of your manuscript.	Yes

Powered by Editorial Manager® and ProduXion Manager® from Aries Systems Corporation

Have you have met the above requirement as detailed in our Minimum Standards Reporting Checklist?	

Manuscript

Click here to access/download;Manuscript;manuscript_Revison2 - URL -

Improved microbial genomes and gene catalog of the chicken gut from metagenomic sequencing of high-fidelity long reads

Yan Zhang*, Fan Jiang*, Boyuan Yang*, Sen Wang, Hengchao Wang, Anqi Wang, Dong Xu, and Wei Fan

Guangdong Laboratory for Lingnan Modern Agriculture (Shenzhen Branch), Genome Analysis Laboratory of the Ministry of Agriculture and Rural Affairs, Agricultural Genomics Institute at Shenzhen, Chinese Academy of Agricultural Sciences, Shenzhen, Guangdong, 518120, China.

*These authors contributed equally to this work. Correspondence should be addressed to fanwei@caas.cn.

Yan Zhang [0000-0003-2281-7807];
Fan Jiang [0000-0003-1359-0970];
Boyuan Yang [0000-0001-7116-8167];
Sen Wang [0000-0001-9793-4472];
Hengchao Wang [0000-0002-8754-4195];
Anqi Wang [0000-0001-9367-2524];
Dong Xu [0000-0001-6180-182X];
Wei Fan [0000-0001-5036-8733]

Abstract

Background: Due to the importance of chicken production and the remarkable influence of the gut microbiota on host health and growth, tens of thousands of metagenome-assembled genomes (MAGs) have been constructed for the chicken gut microbiome. However, due to the limitations of short-read sequencing and assembly technologies, most of these MAGs are far from complete, are of lower quality, and include contaminant reads.
Results: We generated 332 Gb of high-fidelity (HiFi) long reads from the five chicken intestinal compartments and assembled 461 and 337 microbial genomes, of which 53% and 55% are circular, at the species and strain levels, respectively. For the assembled microbial genomes, approximately 95% were regarded as complete according to the “RNA complete” criteria, which requires at least one full-length rRNA operon encoding all three types of rRNA (16S, 23S and 5S) and at least 18

copies of full-length tRNA genes. In comparison with the short-read-derived chicken MAGs, 384 (83% of 461) and 89 (26% of 337) strain-level and species-level genomes in this study are novel, with no matches to previously reported sequences. At the gene level, one-third of the 2.5 million genes in the HiFi-derived gene catalog are novel and cannot be matched to the short-read-derived gene catalog. Moreover, the HiFi-derived genomes have much higher continuity and completeness, as well as lower contamination; the HiFi-derived gene catalog has a much higher ratio of complete gene structures. The dominant phylum in our HiFi-assembled genomes was Firmicutes (82.5%), and the foregut was highly enriched in five genera *Ligilactobacillus*, *Limosilactobacillus*, *Lactobacillus*, *Weissella*, and *Enterococcus*, all of which belong to the order Lactobacillales. Using GTDB-Tk, all 337 species-level genomes were successfully classified at the order level; however, 2, 35 and 189 genomes could not be classified into any known family, genus, and species, respectively. Among these incompletely classified genomes, 9 and 49 may belong to novel genera and species, respectively, because their 16S rRNA genes have identities lower than 95% and 97% to any known 16S rRNA genes.

Conclusions: HiFi sequencing not only produced metagenome assemblies and gene structures with markedly improved quality, but also recovered a substantial portion of novel genomes and genes that were missed in previous short-read-based metagenome studies. The novel genomes and species obtained in this study will facilitate gut microbiome and host-microbiota interaction studies, and thereby contribute to the sustainable development of poultry resources.

Introduction

The domestic chicken, *Gallus gallus* (NCBI:txid9031), has long been used as a model avian species, and chicken eggs and meat provide a primary source of animal-derived protein in the human diet. The first draft genome sequence of chicken was published in 2004, providing unique perspectives on vertebrate evolution [1]. Subsequent population resequencing studies revealed not only the phylogeny history and population structure of this species but also information about locus selection during chicken domestication [2, 3]. The gut microbiota can degrade dietary polysaccharides, detoxify xenobiotics, produce nutrients and energy sources such as vitamins, amino acids, short-chain fatty acids (SCFA), and can also modulate the immune system, thus playing important roles in chicken nutrition, physiology, immunity, and health. However, the gut microbiota also contains many zoonotic pathogens, posing threats to the poultry industry and to human health [4, 5]. Due to the importance of the chicken gut microbiota, its composition and host-interactions have been studied intensively in recent years.

High-throughput short-read sequencing technologies have extensively facilitated metagenome studies to explore the taxonomic and functional compositions of the chicken gut microbiota. Studies that aim to decipher taxonomic compositions tend to sequence 16S rRNA gene amplicons [6, 7], while studies that focus on both taxonomy and functions have used whole-genome shotgun sequencing [8]. In 2018, Huang et al. constructed the first comprehensive gene catalog of the chicken gut microbiome containing ~9 million genes through sequencing of 495 chicken samples from seven different farms in China [9]. Then, several endeavors have been made to construct the metagenome assembled genomes (MAGs) from the fragmented contigs. In 2020, Glendinning et al.

constructed 469 draft MAGs using the gut metagenomes of 24 chicken samples [10]. In 2021, Segura-Wang et al. reconstructed 155 MAGs from metagenomes of 751 chicken samples [11]; Gilroy et al. constructed over 5,595 MAGs based on 632 chicken metagenomes [12]; Feng et al. assembled 12,339 MAGs by integrating 799 public chicken gut microbiome samples from ten countries [8]. These MAGs and gene catalogs constructed from short-read metagenome data provide an overview of the chicken gut microbiota landscape.

Due to the technical limitation of short-read sequencing, these metagenome assemblies often produce fragmented contigs, with a contig N50 less than 10 kb, and a certain portion of contigs that are less than 500 bp in length are usually excluded for downstream analyses [9]. Although these short contigs can be grouped into MAGs with binning algorithms, binning introduces several types of errors, such as incompleteness and contamination [13]. Therefore, MAGs cannot be taken as microbial reference genomes. Indeed, a considerable portion of the gene structures in the non-redundant gene catalog are incomplete, limiting their use in various applications. The advent of highly accurate long-read HiFi (high-fidelity) sequencing promises to resolve these problems. Recently, a sheep fecal metagenome study using ~200 Gb HiFi read data assembled by metaFlye produced 44 circular contigs, each corresponding to a complete reference genome [14, 15]. Furthermore, using the same data, Hifiasm-meta software generated even better assembly result, producing 279 circular complete reference genomes [16]. In this study, we used high-fidelity long-read technology to improve the metagenome assemblies and gene catalogs of the chicken gut microbiomes.

Results

Longer contigs of the chicken metagenome assembled from high-fidelity long reads

We collected 150 digesta samples from the five intestinal compartments (duodenum, jejunum, ileum, cecum, and colorectum) of 30 chickens (Lingnan yellow broilers) slaughtered on Day 42, extracted the metagenomic DNA and combined the DNA samples, evaluated the DNA quality and quantity (Table S1 and Figure S1), and constructed sequencing libraries for each intestinal compartment. Then, we generated 22 Gb, 45 Gb, 73 Gb, 81 Gb, and 112 Gb PacBio HiFi reads for duodenum, jejunum, ileum, cecum, and colorectum, respectively (Table 1). For the total 332 Gb HiFi reads, the N50 read length is 17 kb, and the median read quality value is 32; these values are comparable to those of previous HiFi metagenome studies [14, 16]. The increasing amount of HiFi reads from the duodenum to the colorectum was associated with the increase in microbial diversity along the different intestinal compartments [9], permitting the recovery of more microbial species.

We assembled the HiFi reads into contigs for each intestinal compartment independently with Hifiasm-meta [16], which produced linkage graphs of the contigs. Taking the colorectum as an example, we observed a single “super complex”, several tangled circular, hundreds of circular, and many linear topologies in the contig graph (Figure 1, Figure S2). Upon reviewing the taxonomic components and read coverage depth for each topology, we found that the super complex contains tens of various microbial genomes sharing some similar genomic fragments; the tangled circles contain many different strains of one species, and the high redundancy of overlapped contigs makes the tangled circles seem much larger than the real genome size of the species; and the circular and linear contigs represent complete and incomplete genomes for single microbial strain or species,

respectively.

The total contig sizes are 0.22 Gb, 0.56 Gb, 0.85 Gb, 3.11 Gb, and 3.96 Gb and the contig N50 sizes are 28 kb, 29 kb, 34 kb, 193 kb and 165 kb for the duodenum, jejunum, ileum, cecum, and colorectum, respectively (Figure 2ab, Table S2). In comparison, the contig N50 sizes from short-read metagenome assemblies are usually lower than 10 kb [9], suggesting that HiFi reads assembly provides a substantial improvement in contig continuity. The foregut (duodenum, jejunum, ileum) assemblies contain more fragmented contigs than the hindgut (cecum, colorectum), which may be explained by the fact that the foregut contains only a few of dominant microbial species and other species with very low abundance. In comparison, the hindgut (cecum, colorectum) contains hundreds of abundant microbial species, and their abundance distribution is relatively more even. Although genomic complexity may also lead to fragmented contigs, we observed a non-trivial correlation between contig size and coverage depth, indicating that insufficient coverage depth of microbes with very low abundance is the primary reason for most of the fragmented contigs (Figure 2c, Figure S3). Moreover, the coverage depth is positively related with the single-base quality values, indicating that higher coverage depth will improve the single-base accuracy of the contig sequences (Figure 2d).

Hundreds of complete circular genomes and binned non-circular MAGs

For the duodenum, jejunum, ileum, cecum, and colorectum, respectively, we obtained 22, 25, 41, 120 and 173 reference microbial genomes of circular contigs and recovered 5, 15, 21, 165 and 161 metagenome-assembled genomes (MAGs) from the binning of non-circular contigs, resulting in a total of 27, 40, 62, 285 and 334 assembled microbial genomes that passed the medium-quality criteria (Figure 3a, Table S3). Most of the circular genomes met the near-complete criteria, while the non-circular MAGs include more genomes with relatively lower qualities, referred to as high-quality and medium-quality. Previously, the Hifiasm-meta project used a small portion of the data generated in this study for software testing and assembled 62 circular microbial genomes that met near-complete criteria using 33.6 Gb of chicken cecum data [16]. In this study, using a total of 81 Gb of cecum data, we successfully assembled 110 circular microbial genomes with near-complete quality. This result indicates that more complete genomes can be assembled by increasing the sequencing depth.

For the assembled microbial genomes within each intestinal compartment, the sequence divergences are mostly above 1%. i.e. have an average nucleotide identity (ANI) below 99%, which represents a strain-level assembly. To remove the assembly redundancy among intestinal compartments, we removed redundant genomes (those with a sequence divergence lower than 1%) and thereby generated 461 non-redundant genomes of microbial strains within the chicken gut (Figure 3a). Furthermore, to remove the redundant genomes at the species level, these 461 non-redundant microbial strain genomes were reduced to 337 non-redundant genomes with sequence divergences greater than 5%. Of the 461 strain-level and 337 species-level microbial genomes, 246 (53%) and 187 (55%) are circular genomes, respectively. According to the distribution analysis, the circular genomes have larger assembly sizes and higher CheckM scores than the non-circular MAGs (Figure 3bc), and the assembled genome sizes are positively correlated with the CheckM scores (Figure S4). Using the 187 circular species-level genomes, which all have complete genome assemblies, we

showed that higher coverage depth is positively correlated with CheckM completeness score, indicating that a higher coverage depth will improve the single-base accuracy of the genome assemblies (Figure S5).

Although plasmids were reported to be more difficult to assemble than host genomes in metagenomes [17], we were able to identify 61, 67, 71, 81 and 78 circular plasmid genomes in the Hifiasm-meta contigs for duodenum, jejunum, ileum, cecum, and colorectum, respectively (Table S4). Moreover, we identified 33, 14, 14, 52 and 50 circular viral genomes among the corresponding intestinal compartments. The average plasmid genome size is 69 kb, which is slightly larger than the average virus genome size of 52 kb. The success in assembling these circular plasmid and virus genomes is encouraging; many more plasmid and virus fragments exist in the tangled or linear contigs and should be investigated further.

The presence of rRNA and tRNA genes confirms the high assembly quality

In prokaryotes, the 5S, 16S, and 23S ribosomal RNA (rRNA) genes are commonly colocated and transcribed together, forming rRNA operons. Usually, multiple copies of rRNA operons exist in one genome, and the repetitive nature makes them difficult to assemble from short reads. Transfer RNA (tRNA) genes are randomly distributed in the genome, often in multiple redundant copies. The identification of rRNA and tRNA genes has traditionally been used as an important measurement for the completeness of genome assembly [16]. We annotated the rRNA and tRNA genes in the 461 non-redundant microbial genomes, and found that 447 (97%) genomes have at least one full-length rRNA operon encoding all three types of rRNA (5S, 16S, 23S) genes, 450 (98%) genomes have at least 18 copies of full-length tRNA genes, and 439 (95%) genomes are "RNA complete", meeting both the rRNA and tRNA criteria. Our results showed that most microbial genomes have 1-6 rRNA operons (Figure 4a) and 35-65 copies of tRNA genes (Figure 4b). In addition, the number of rRNA operon and tRNA genes in circular genomes is larger than that in non-circular MAGs (Figure 4ab), which is consistent with the results of the completeness analysis of the microbial genomes.

Superiority of HiFi assembled genomes over short-read assembled MAGs

Numerous efforts have been made to construct MAGs from short-read assembled contigs [18]. A recent study on the chicken gut metagenome reported the generation of 12,339 dereplicated strain-level MAGs (ANI < 99%) and 1,978 dereplicated species-level MAGs (ANI < 95%) by integrating the short-read assembly of 799 public chicken gut microbiome samples from ten countries [8]. Compared to the reported strain-level MAGs, 384 (83%) of our 461 strain-level genomes are novel (ANI < 99%), including 209 (45%) circular genomes and 175 (38%) non-circular MAGs (Figure 5a). Compared to the reported species-level MAGs, 89 (26%) of our 337 species-level genomes are novel (ANI < 95%), including 50 (15%) circular genomes and 39 (12%) non-circular MAGs (Figure 5b). Although the currently limited sample sizes and HiFi sequencing depth produces a smaller number of assembled microbial genomes than are generated by the short-read assembly (Figure 5c), HiFi assembly can recover genomes of novel species and especially novel strains, which cannot be successfully resolved by short read assembly, because short reads cannot distinguish the highly similar sequences of closely related microorganisms.

The quality of the HiFi assembled microbial genomes is highly superior to that of the short-read assembled MAGs. The average contig numbers for our assemblies are 1 for our circular genomes and 2.8 for non-circular MAGs, in comparison to 257 for the short-read assembled MAGs (Figure 5d). Our average assembled genome sizes are 2.61 Mb, 2.35 Mb, and 2.23 Mb, and the average contig N50 sizes are 2,884 kb, 1,697 kb and 38 kb for the circular genomes, non-circular MAGs, and short-read MAGs, respectively (Figure 5e-f). Moreover, the average CheckM completeness percentages are 95.5, 76.4 and 89.5, and the average CheckM contamination percentages are 0.85, 1.59, and 2.14 for the circular genomes, non-circular MAGs, and short-read MAGs, respectively (Figure 5g-h). Almost all the evaluations of our circular genomes and non-circular MAGs are better or much better than those of the short-read assembled MAGs, except for the CheckM completeness of our non-circular MAGs, which is slightly lower than that of the short-read MAGs, because the two genome datasets used different completeness cutoffs (50% versus 80%). Overall, the HiFi-assembled microbial genomes are not only more continuous and complete than the short-read MAGs but also have less contamination.

Advantage of HiFi-derived gene catalog over gene catalogs from short-reads

In addition to MAGs, the non-redundant gene catalog is another important resource in metagenome studies. Based on Illumina sequencing data, in 2018, Huang et al. published the first 9.0 M gene catalog (CGM-RGC) for the chicken gut metagenome [9], and in 2021, Feng et al. published a more comprehensive 16.6 M gene catalog (GG-IGC) [8] that integrated all the available public chicken metagenome sequencing data. Here, we constructed a 2.5 M non-redundant gene catalog (HiFi-RGC) with the HiFi assembled contigs from all intestinal compartments. Although the gene number of our gene catalog is smaller than those of the two published gene catalogs due to the limited sample sizes, the structure completeness ratio of our gene catalog is 99%, much higher than the 38% and 63% reported for CGM-RGC and GG-IGC, respectively (Figure 6a-b).

By comparing the pairwise overlap at the gene sequence level, we found that 847,801 (33.8%) and 724,123 (28.9%) genes are unique in HiFi-RGC compared to CGM-RGC and GG-IGC, respectively (Figure 6c-d), suggesting that the HiFi-derived gene catalog recovered a substantial portion of the genes that were missed by short-read technologies. Because GG-IGC is more comprehensive than CGM-RGC, we considered the 724,123 (28.9%) genes in HiFi-RGC as unique genes and the remaining genes (71.1%) in HiFi-RGC as shared genes. Then, the microbial communities derived from the unique and shared genes in HiFi-RGC were compared. The results showed that 36.8% of unique genes were unclassified at the phylum level, which was obviously higher than the proportion of shared genes (24.9%), suggesting that the unique genes are enriched in unknown phyla (Figure S6).

Phylogeny of HiFi assembled microbial genomes and differences among intestinal compartments

We used GTDB-Tk to align the 337 HiFi assembled species-level genomes to the 47,894 species clusters (45,555 bacterial and 2,339 archaeal) in the GTDB database (r202) and assign taxonomic classification to the HiFi assembled genomes based on their phylogenetic placement [19]. Only one genome was classified as archaea, and the other 336 genomes were all classified as bacteria. The

dominant phylum is Firmicutes containing 278 (82.5%) genomes, followed by Bacteroidota and Actinobacteriota, which contain 25 (7.4%) and 14 (4.2%) genomes, respectively. In total, these three phyla covered 317 (94%) of all the assembled genomes. The remaining genomes were classified as Cyanobacteria, Proteobacteria, Desulfobacterota, Campylobacterota, Deferribacterota, Methanobacteriota, and Verrucomicrobiota.

The foregut contains the duodenum, jejunum, and ileum, which mainly function in feed digestion and nutrient absorption. The hindgut contains the cecum and colorectum, which function in fermentation, detoxification and recycling of residual water and salt. Noticeably, there was a distinctive difference in the microbial composition between the foregut and hindgut. The foregut was highly enriched in five genera *Ligilactobacillus*, *Limosilactobacillus*, *Lactobacillus*, *Weissella*, and *Enterococcus*, all belonging to the order Lactobacillales. In contrast, the species diversity of the hindgut was much higher, and the species were more dispersed (Figure 7, Figure S7). This difference in species composition between the foregut and hindgut is consistent with previous reports from short-read metagenome studies [9], and is caused by the difference in morphology and physiology between the foregut and hindgut. The sampling of all intestinal compartments contributes to more comprehensive microbial genome assemblies. Microbes of very low abundance in some intestinal compartments but of relatively higher abundance in other compartments could also be recovered.

Novel genomic representation and novel genus and species discovery

Although all the species-level genomes have been successfully classified at the order level by GTDB-Tk [19], 2, 35 and 189 genomes could not be classified at the lower taxonomic levels of family, genus, and species, respectively, suggesting that they are novel genome assemblies for these families, genera, and species (Figure 7). Some of these new genomic sequences may have potential benefits to industry or medical applications. *Lactobacillus* has traditionally been used in the fermentation industry, producing lactate from raw carbohydrates and synthetic media [20]. In recent years, *Lactobacillus* and its close relatives *Ligilactobacillus* and *Limosilactobacillus* have also been widely adopted as probiotic supplements, either in animal feed to promote growth or human foods to improve human health [21]. Among our 337 assembled microbial species genomes, 3 genomes belong to *Lactobacillus,* 7 genomes belong to *Ligilactobacillus*, and 6 genomes belong to *Limosilactobacillus*. All these genomes have been successfully classified to the genus level, and most of the genomes were successfully classified to the species level by GTDB-Tk. However, 2 *Ligilactobacillus* genomes and 1 *Limosilactobacillus* genome have not been classified to the species level, suggesting that these 3 species-level genomes may represent novel genomic resources for probiotic development.

To further classify these genomes at lower taxonomic ranks, we used the Ribosomal Database Project (RDP) Classifier and alignments to the Silva 16S rRNA database with the annotated 16S rRNA gene sequences from their genomes. RDP successfully classified one genome at the family level and 14 genomes at the genus level, leaving 1, 21, and 189 genomes still unclassified at the family, genus, and species levels, respectively (Table S5). Then, the alignment identities to the Silva database were used to validate the taxonomic novelty for these genomes. We found that 58 genomes have 16S-rRNA gene identities lower than 97%, which is the threshold for demarcating bacterial

species [22]. Among these, 9 genomes have 16S-rRNA gene identities lower than 95%, which is the threshold generally used to delineate a new genus [23], indicating that 9 and 49 of these genomes may correspond to novel genera and species, respectively, which broadens our knowledge of the microbial world (Table S6-7). In addition, approximately one-third of these newly discovered genera and half of these newly discovered species were not found in the short-read MAG data, suggesting that they are derived only from HiFi metagenome data, which further shows the advantage of HiFi sequencing in metagenomic studies.

Conclusions

Given the importance of chicken production and the remarkable contribution of the intestinal microbiota to host nutrition and health, numerous efforts have been made to construct chicken gut MAGs and gene catalogs. In the present study, using high-fidelity long reads of the five intestinal compartments of chickens, we assembled 461 microbial genomes at strain level (ANI > 99%) and 337 microbial genomes at species level (ANI > 95%), of which 246 (53%) and 187 (55%) are circular genomes, respectively. In addition, many circular plasmids and viral genomes were also successfully obtained. Among the 461 microbial genomes, 439 (95%) genomes are "RNA complete", having at least one full-length rRNA operon coding for all three types of rRNA (16S, 23S and 5S rRNA) genes and at least 18 copies of full-length tRNA genes. With this work, chicken is now the third animal species after human and sheep that have comprehensive HiFi gut metagenome assemblies.

In comparison to the chicken MAGs derived from short-read metagenome assemblies, the HiFi-assembled microbial genomes not only provide substantial advantages in continuity, completeness, and contamination metrics, but also recovered 384 (83% of 461) and 89 (26% of 337) novel strains and species, respectively. In addition, the structure completeness ratio of the 2.5 M non-redundant gene catalog constructed from HiFi assembled contigs (>99%) is much higher than that of the short-read assembly derived gene catalogs (40-60%), and approximately one-third of the genes in the HiFi-derived gene catalog are not present in the short-read derived gene catalogs. Taken together, our results showed that HiFi metagenome sequencing not only yields genomes and genes with better qualities, but also provides a substantial number of novel genomes and genes that were missed in short-read metagenome studies.

Phylogeny analysis showed that the dominant phyla in our HiFi assembled genomes are Firmicutes (82.5%), Bacteroidota (7.4%) and Actinobacteriota (4.2%). The foregut is highly enriched in five genera in Lactobacillales (order), *Ligilactobacillus*, *Limosilactobacillus*, *Lactobacillus*, *Weissella*, and *Enterococcus*, whereas the hindgut has a much wider spectrum of species. Using GTDB-Tk, 2, 35 and 189 genomes failed to be classified at the family, genus, and species levels, suggesting that they are novel assembled genomes at these respective levels. The RDP Classifier further assigned one genome at the family level and 14 genomes at the genus level. Among the remaining unclassified genomes, 9 and 49 genomes have 16S rRNA gene identities lower than 95% and 97% in the Silva database, indicating that these genomes may represent novel genera and species, respectively. The HiFi metagenome assembly not only improves the genomic representation but also enables the discovery of novel taxonomic units. With regard to chicken production, these novel

microbial genomes or species will serve as a valuable resource for future studies of functions such as feed digestion and fermentation as well as the mechanisms of disease-prevention and growth-promotion effects of antibiotics and alternatives.

Methods

Chicken husbandry and disease prevention

Lingnan yellow broilers were studied for a 42-day feeding trial, with free access to feed and water. The baby chicks were purchased from Zhiwei Guangdong company at 1 day of age, and raised in battery cages at the farmhouse of the Agricultural Genomics Institute of Shenzhen. The lighting schedule was 16 h light and 8 h dark throughout the experiment. The room temperature was controlled with heaters, gradually reduced from 35 °C on Day 1 to 24 °C on Day 21, and then maintained at 24 °C until Day 42. The diets were based on the Nutrient Requirements of Poultry: Ninth Revised Edition, 1994 (NRC, 1994) and Feeding Standard of Chicken (NY/T 33-2004).

The chickens were injected with Marek's Disease Vaccine and Cephalosporin on Day 1, vaccinated against Newcastle disease virus (NDV, La Sota) and infectious bronchitis virus (IBV, H120) on Day 7 through intranasal administration, vaccinated against NDV La Sota and IBV M41 and avian influenza H9-NJ02 on Day 9 through hypodermic injection, vaccinated against infectious bursal disease virus (IBD B87) on Day 14 through water drinking, vaccinated against fowlpox virus (FPV, CVCC AV1003) on Day 21 through wing puncture, vaccinated against Newcastle disease virus (NDV, La Sota) on Day 28 in the drinking water. The chicks also received preventative treatment for coccidiosis and other parasitic diseases with the application of diclazuril on Days 17-18, sulfaquinoxaline on Days 24-25, and albendazole on Days 31-32.

Body weight records and digesta sample collections

The body weight and feed intake of the chickens were recorded for each replicate on Day 42. The average feed intake was 3.74 Kg, the average body weight was 1.99 Kg, and the feed conversion ratio was 1.93, which are consistent with the growth characteristics of this chicken breed. Then, randomly selected chickens were slaughtered on Day 42, and the intestines were immediately removed and dissected. Fresh digesta samples from the duodenum, jejunum, ileum, cecum, and colorectum were collected and frozen in a dry-ice pack, transported to the laboratory and stored at -80 °C until DNA extraction.

DNA extraction, library preparation, and sequencing

The digesta samples for each intestinal compartment from a total of 30 chickens were collected for metagenomic DNA extraction. Mainly due to the volume of digesta, it was difficult to process all of the samples at one time. For the convenience of processing, the duodenum digesta from every 5 chickens were pooled together and then washed for microbial cell enrichment and DNA extraction. After processing all duodenum samples, the metagenomic DNA was finally pooled, and further purified with VAHTS DNA Clean Beads (N411-02, Vazyme). The metagenomic DNA samples of the jejunum, ileum, cecum and colorectum were processed in the same way, except that for the cecum, due to its relatively high microbial density, only a subfraction of the pooled and thoroughly mixed digesta was used for microbial cell enrichment and DNA extraction.

The following steps were performed for microbial cell enrichment. The pooled digesta samples were mixed thoroughly with saline buffer containing 0.1% Tween 80 (pre-cooled at 4 °C) by vortexing. The microbial cells were separated through differential centrifugation to remove the undigested feed particles [9] and DNA was extracted from the enriched microbial cells with a DNeasy PowerSoil Pro kit (47014, Qiagen). For the bead beating and lysis options of the DNeasy PowerSoil Pro kit, we added approximately 200 mg of the enriched cells and 800 uL of Solution CD1 into each PowerBead Pro Tube. The tubes were vortexed briefly to mix and incubated at 65 °C for 10 min before the bead beating step. Then, the tubes were placed horizontally and properly balanced on a Vortex Adapter for 24 (1.5–2.0 ml) tubes (QIAGEN, 13000-V1-24) on a Kylin-Bell VORTEX-6. The samples were vortexed in the tubes at maximum speed for 10 min. To ensure the efficiency of the homogenization step, fewer than 12 tubes were vortexed at one time. All the other steps were carried out according to the manufacturer's standard protocol.

The DNA quality and quantity were measured by a Invitrogen Qubit 4 Fluorometer with Qubit™ dsDNA BR (Invitrogen, Q32850) and by a Nanodrop 2000c Microvolume Spectrophotometer. The integrity of the DNA was evaluated on field electrophoresis agarose gels. The high-integrity genomic DNA was fragmented to 15-20 kb using g-TUBEs (Covaris, USA), and sequencing libraries were prepared by SMRTbell Express Template Prep Kit 2.0 (PacBio, USA). Then, high-fidelity long reads were generated on a PacBio Sequel II (PacBio Sequel II System, RRID:SCR_017990) in Circular Consensus Sequence (CCS, RRID:SCR_021174) mode (PacBio, USA). Because microbial diversity gradually increases from the head to the end point of the intestinal tract, one, two, two, three, and three PacBio CCS cells were used for sequencing the duodenum, jejunum, ileum, cecum, and colorectum, respectively.

Metagenome contig assembly and MAG binning

To ensure assembly quality, the raw HiFi sequencing reads were filtered, requiring read lengths over 2 kb and average read accuracy over 99%. In addition, the remaining reads were mapped to the host chicken genome and feed genomes (maize and soybean) by minimap2 (Minimap2, RRID:SCR_018550) v2-2.20 [24] with parameter "-x map-hifi" to remove contaminant sequences, eliminating approximately 2%, 0.5%, 0.5%, 0.1%, and 0.1% of the reads for the duodenum, jejunum, ileum, cecum, and colorectum samples, respectively. Hifiasm-meta (hifiasm-meta, RRID:SCR_022771) r058 [16] with default parameters was used to assemble the pre-filtered HiFi reads into contigs. By exploiting the contig linkages from the resulting GFA files with Bandage (Bandage, RRID:SCR_022772) v0.8.1 [25], the Hifiasm-meta contigs were divided into 3 classes: (1) circular contig, complete genome assembly of a given species; (2) tangled "circular", many fragmented contigs linked into a tangled circular genome, formed by various heterozygous strains of a species; and (3) linear contig, representing incomplete genome assembly of a species, often due to low coverage depth. The circular contigs were left alone, and each tangled "circular" was independently re-assembled by Hifiasm-meta r058 with default parameters, using these fragmented contigs as input reads. Furthermore, the linear contigs were grouped into metagenome assembled genomes (MAGs) by a binning algorithm MetaBAT2 (MetaBAT, RRID:SCR_019134) v2.12.1 with the parameter "-a depth_file" [18] using the contig depth obtained from the Hifiasm-meta GFA files. CheckM (CheckM, RRID:SCR_016646) (lineage_wf) v1.1.3 [26] with parameter "lineage_wf" was utilized to evaluate the assembly quality, and 3 quality ranks were adopted: near complete (≥ 90%

completeness and < 5% contamination), high-quality (≥ 70% completeness and < 10% contamination), and medium-quality (≥ 50% completeness and < 10% contamination).

Construction of non-redundant microbial genome assemblies

The sequencing data of each intestinal compartment (duodenum, jejunum, ileum, cecum, and colorectum) was assembled independently, due to the limitations of our computer memory. Then, the microbial genome assemblies (near-complete, high-quality, medium-quality) from all intestinal compartments were put together, and pairwise identity (0–100) was calculated by FastANI (FastANI, RRID:SCR_021091) v1.32 [27] with default parameters. The identity values were converted into distance values by (100 - identity)/100, and a hierarchical clustering algorithm with maximum distance was applied [28]. The stop distances for hierarchical clustering were set to 0.01 and 0.05 to obtain strain-level and species-level clusters, respectively. Then, in each cluster, a circular genome was preferred over a non-circular MAG; in addition, a genome assembly with a larger CheckM score (completeness – 5 * contamination) was preferred. After taking the best genome assembly as the representative, the other genome assemblies were taken as redundance and removed. Finally, the non-redundant sets of microbial genomes at the strain-level (ANI 99%) and the species-level (ANI 95%) were generated, respectively.

Taxonomy classification and genome annotation

GTDB-Tk (GTDB-Tk, RRID:SCR_019136) (classify_wf) v1.5.1 [19] with parameter "classify_wf" and its database version r202 were used for phylogenetic placement and classification of the assembled microbial genomes, and GraPhlAn (GraPhlAn, RRID:SCR_016130) v1.1.3 [29] was used for tree visualization. The Ribosomal Database Project (RDP) (Ribosomal Database Project, RRID:SCR_006633) Classifier (RDP Classifier, RRID:SCR_022773) V2.11 [30, 31] was used to classify the genome lower taxonomic ranks with 16S rRNA gene sequences, requiring >= 70% confidence. The best hits of the BLAST (BLAST, RRID:SCR_008419) V2.3.1 alignments with the parameters "blastn -task megablast -evalue 1e-5" to the Silva database (r138) [32] were further used to validate the novelty of the taxonomic units. ViralVerify (viralVerify, RRID:SCR_022774) v1.1 [33] with the parameter "--hmm nbc_hmms.hmm" was adopted to classify the assembled genomes into bacteria/archaea, plasmid and viral genomes. RNAmmer (RNAmmer, RRID:SCR_017075) v1.2 [34] with the parameter "-S arc/bac -m lsu,ssu,tsu" was adopted to annotate the 5S, 16S, and 23S rRNA genes, tRNAscan-SE (tRNAscan-SE, RRID:SCR_010835) v2.0.3 [35] with the parameter "-G -H" was adopted to predict tRNA genes, and Prodigal (Prodigal, RRID:SCR_011936) (v2.6.3) [36] with parameter "-p single" was used to predict protein-coding genes from the assembled microbial genomes.

Non-redundant gene catalog construction

Protein-coding gene prediction was performed on the contigs of each intestinal compartment by Prodigal (v2.6.3) [36] with the parameter "-p meta". Then, to obtain a non-redundant chicken gut gene catalog at the species-level, the gene models from all the intestinal compartments were put together and redundance were removed by the criteria of identity > 95% and overlap > 90% of the shorter genes, using cd-hit-est (CD-HIT, RRID:SCR_007105) v4.6.6 [37] with the parameter "-c 0.95 -n 10 -G 0 -aS 0.9". Then, the non-redundant gene catalog was taxonomically annotated using Kaiju (Kaiju, RRID:SCR_022775) v1.9.0 [38] with the option "-a greedy" based on the NCBI-NR

v2020-03-20 database.

To compare the overlap of our gene catalog (HiFi-RGC) with two published chicken gut metagenome gene catalogs (CGM-RGC and GG-IGC) [8, 9], pairwise alignments of HiFi-RGC to CGM-RGC and HiFi-RGC to GG-IGC were performed using BLAT (BLAT, RRID:SCR_011919) [39] with identity ≥ 95% and overlap ≥ 90% of the shorter genes as the criteria for shared genes.

Data availability

The HiFi sequencing reads can be found under BioProject ID PRJNA748109: SRR19683891 for duodenum, SRR19732514 and SRR19726169 for jejunum, SRR19736685 for ileum, SRR15214153 and SRR19732730 for cecum, and SRR19683890 and SRR19732729 for colorectum. The assembled contigs, microbial genomes for each intestinal compartments, non-redundant genome sets at species and strain levels, non-redundant gene catalog, and plasmid and viral annotations are available at AGIS website [40]. All supporting data are available in the *GigaScience* GigaDB database [41].

List of abbreviations

circular consensus sequence (CCS)
high-fidelity (HiFi)
metagenome-assembled genomes (MAGs)
circular and complete metagenome-assembled genomes (circular MAGs)
non-circular and incomplete metagenome-assembled genomes (non-circular MAGs)
average nucleotide identity (ANI)
non-redundant gene catalog derived from HiFi data(HiFi-RGC)
the first 9.0 M gene catalog for the chicken gut metagenome (CGM-RGC)
the integrated comprehensive 16.6 M gene catalog (GG-IGC)
Genome Taxonomy Database (GTDB)
Ribosomal Database Project (RDP)
assembly quality of ≥ 90% completeness and < 5% contamination (near-complete)
assembly quality of ≥ 70% completeness and < 10% contamination (high-quality)
assembly quality of ≥ 50% completeness and < 10% contamination (medium-quality)

Acknowledgements

We thank Dan Liu and Wenshu Liu for suggestions chicken breed selection. We thank Yazhi Qin for assistance in raising and slaughtering the chickens. The work was funded by the National Natural Science Foundation of China (Grant No. 32000408), the Agricultural Science and Technology Innovation Program of CAAS, and fund of Key Laboratory of Shenzhen (ZDSYS20141118170111640).

Author contributions

Y.Z. and W.F. designed and coordinated the research. Y.Z. and B.Y. prepared the chicken gut materials for sequencing. F.J. and B.Y. performed the data analysis. W.F. wrote the manuscript, and all authors provided suggestions and revised the manuscript.

Ethics approval

This study was approved by the Life Science Ethics Committee of Agricultural Genomics Institute, Chinese Academy of Agricultural Sciences.

Competing interests

The authors declare no competing interests.

References

1. International Chicken Genome Sequencing C. Sequence and comparative analysis of the chicken genome provide unique perspectives on vertebrate evolution. Nature. 2004;432 7018:695-716. doi:10.1038/nature03154.
2. Wong GK, Liu B, Wang J, Zhang Y, Yang X, Zhang Z, et al. A genetic variation map for chicken with 2.8 million single-nucleotide polymorphisms. Nature. 2004;432 7018:717-22. doi:10.1038/nature03156.
3. Rubin CJ, Zody MC, Eriksson J, Meadows JR, Sherwood E, Webster MT, et al. Whole-genome resequencing reveals loci under selection during chicken domestication. Nature. 2010;464 7288:587-91. doi:10.1038/nature08832.
4. Yeoman CJ, Chia N, Jeraldo P, Sipos M, Goldenfeld ND and White BA. The microbiome of the chicken gastrointestinal tract. Anim Health Res Rev. 2012;13 1:89-99. doi:10.1017/S1466252312000138.
5. Oakley BB, Lillehoj HS, Kogut MH, Kim WK, Maurer JJ, Pedroso A, et al. The chicken gastrointestinal microbiome. FEMS Microbiol Lett. 2014;360 2:100-12. doi:10.1111/1574-6968.12608.
6. Sergeant MJ, Constantinidou C, Cogan TA, Bedford MR, Penn CW and Pallen MJ. Extensive microbial and functional diversity within the chicken cecal microbiome. PLoS One. 2014;9 3:e91941-e. doi:10.1371/journal.pone.0091941.
7. Wen C, Yan W, Sun C, Ji C, Zhou Q, Zhang D, et al. The gut microbiota is largely independent of host genetics in regulating fat deposition in chickens. The ISME journal. 2019;13 6:1422-36. doi:10.1038/s41396-019-0367-2.
8. Feng Y, Wang Y, Zhu B, Gao GF, Guo Y and Hu Y. Metagenome-assembled genomes and gene catalog from the chicken gut microbiome aid in deciphering antibiotic resistomes. Commun Biol. 2021;4 1:1305. doi:10.1038/s42003-021-02827-2.
9. Huang P, Zhang Y, Xiao K, Jiang F, Wang H, Tang D, et al. The chicken gut metagenome and the modulatory effects of plant-derived benzylisoquinoline alkaloids. Microbiome. 2018;6 1:211. doi:10.1186/s40168-018-0590-5.

10. Glendinning L, Stewart RD, Pallen MJ, Watson KA and Watson M. Assembly of hundreds of novel bacterial genomes from the chicken caecum. Genome Biol. 2020;21 1:34. doi:10.1186/s13059-020-1947-1.
11. Segura-Wang M, Grabner N, Koestelbauer A, Klose V and Ghanbari M. Genome-Resolved Metagenomics of the Chicken Gut Microbiome. Front Microbiol. 2021;12:726923. doi:10.3389/fmicb.2021.726923.
12. Gilroy R, Ravi A, Getino M, Pursley I, Horton DL, Alikhan NF, et al. Extensive microbial diversity within the chicken gut microbiome revealed by metagenomics and culture. PeerJ. 2021;9:e10941. doi:10.7717/peerj.10941.
13. Yue Y, Huang H, Qi Z, Dou HM, Liu XY, Han TF, et al. Evaluating metagenomics tools for genome binning with real metagenomic datasets and CAMI datasets. BMC Bioinformatics. 2020;21 1:334. doi:10.1186/s12859-020-03667-3.
14. Bickhart DM, Kolmogorov M, Tseng E, Portik DM, Korobeynikov A, Tolstoganov I, et al. Generating lineage-resolved, complete metagenome-assembled genomes from complex microbial communities. Nat Biotechnol. 2022;40 5:711-9. doi:10.1038/s41587-021-01130-z.
15. Kolmogorov M, Bickhart DM, Behsaz B, Gurevich A, Rayko M, Shin SB, et al. metaFlye: scalable long-read metagenome assembly using repeat graphs. Nat Methods. 2020;17 11:1103-10. doi:10.1038/s41592-020-00971-x.
16. Feng X, Cheng H, Portik D and Li H. Metagenome assembly of high-fidelity long reads with hifiasm-meta. Nat Methods. 2022; doi:10.1038/s41592-022-01478-3.
17. Pellow D, Zorea A, Probst M, Furman O, Segal A, Mizrahi I, et al. SCAPP: an algorithm for improved plasmid assembly in metagenomes. Microbiome. 2021;9 1:144. doi:10.1186/s40168-021-01068-z.
18. Kang DD, Li F, Kirton E, Thomas A, Egan R, An H, et al. MetaBAT 2: an adaptive binning algorithm for robust and efficient genome reconstruction from metagenome assemblies. PeerJ. 2019;7:e7359. doi:10.7717/peerj.7359.
19. Chaumeil PA, Mussig AJ, Hugenholtz P and Parks DH. GTDB-Tk: a toolkit to classify genomes with the Genome Taxonomy Database. Bioinformatics. 2019; doi:10.1093/bioinformatics/btz848.
20. Sun Z, Harris HM, McCann A, Guo C, Argimon S, Zhang W, et al. Expanding the biotechnology potential of lactobacilli through comparative genomics of 213 strains and associated genera. Nat Commun. 2015;6:8322. doi:10.1038/ncomms9322.
21. Sarao LK and Arora M. Probiotics, prebiotics, and microencapsulation: A review. Crit Rev Food Sci Nutr. 2017;57 2:344-71. doi:10.1080/10408398.2014.887055.
22. Stackebrandt E and Goebel BM. Taxonomic note: A place for DNA:DNA reassociation and 16s rRNA sequence analysis in the present spec. In: 1994.
23. Ludwig W, Strunk O, Klugbauer S, Klugbauer N, Weizenegger M, Neumaier J, et al. Bacterial phylogeny based on comparative sequence analysis. Electrophoresis. 1998;19 4:554-68. doi:10.1002/elps.1150190416.
24. Li H. Minimap2: pairwise alignment for nucleotide sequences. Bioinformatics. 2018;34 18:3094-100. doi:10.1093/bioinformatics/bty191.
25. Wick RR, Schultz MB, Zobel J and Holt KE. Bandage: interactive visualization of de novo genome assemblies. Bioinformatics. 2015;31 20:3350-2. doi:10.1093/bioinformatics/btv383.
26. Parks DH, Imelfort M, Skennerton CT, Hugenholtz P and Tyson GW. CheckM: assessing the

quality of microbial genomes recovered from isolates, single cells, and metagenomes. Genome Res. 2015;25 7:1043-55. doi:10.1101/gr.186072.114.

27. Srinivas A, Chirag J, Patrick F and Tony P. Fast calculation of Average Nucleotide Identity (FastANI) v1.32 (Version 1.32). GitHub. 2020. https://github.com/ParBLiSS/FastANI.
28. Fan W. Hierarchical clustering algorithm (hcluster.pl) v1.0 (Version 1.0). GitHub. 2019. https://github.com/fanagislab/bioinfo_versatiles/blob/master/hcluster.pl.
29. Asnicar F, Weingart G, Tickle TL, Huttenhower C and Segata N. Compact graphical representation of phylogenetic data and metadata with GraPhlAn. PeerJ. 2015;3:e1029. doi:10.7717/peerj.1029.
30. Wang Q, Garrity GM, Tiedje JM and Cole JR. Naive Bayesian classifier for rapid assignment of rRNA sequences into the new bacterial taxonomy. Appl Environ Microbiol. 2007;73 16:5261-7. doi:10.1128/AEM.00062-07.
31. Qiong W, George MG, James MT and James RC. Ribosomal Database Project (RDP) Classifier v2.11 (Version 2.11). RDP website. 2007. http://rdp.cme.msu.edu/classifier/classifier.jsp.
32. Quast C, Pruesse E, Yilmaz P, Gerken J, Schweer T, Yarza P, et al. The SILVA ribosomal RNA gene database project: improved data processing and web-based tools. Nucleic Acids Res. 2013;41 Database issue:D590-6. doi:10.1093/nar/gks1219.
33. Anton K, Artem S, Mikhail D and Nikolay V. Viral contig verification tool (viralVerify) v1.1 (Version 1.1). GitHub. 2021.https://github.com/ablab/viralVerify.
34. Lagesen K, Hallin P, Rodland EA, Staerfeldt HH, Rognes T and Ussery DW. RNAmmer: consistent and rapid annotation of ribosomal RNA genes. Nucleic Acids Res. 2007;35 9:3100-8. doi:10.1093/nar/gkm160.
35. Chan PP, Lin BY, Mak AJ and Lowe TM. tRNAscan-SE 2.0: improved detection and functional classification of transfer RNA genes. Nucleic Acids Res. 2021;49 16:9077-96. doi:10.1093/nar/gkab688.
36. Hyatt D, Chen GL, Locascio PF, Land ML, Larimer FW and Hauser LJ. Prodigal: prokaryotic gene recognition and translation initiation site identification. BMC Bioinformatics. 2010;11:119. doi:10.1186/1471-2105-11-119.
37. Li W and Godzik A. Cd-hit: a fast program for clustering and comparing large sets of protein or nucleotide sequences. Bioinformatics. 2006;22 13:1658-9. doi:10.1093/bioinformatics/btl158.
38. Menzel P, Ng KL and Krogh A. Fast and sensitive taxonomic classification for metagenomics with Kaiju. Nat Commun. 2016;7:11257. doi:10.1038/ncomms11257.
39. Kent WJ. BLAT--the BLAST-like alignment tool. Genome Res. 2002;12 4:656-64. doi:10.1101/gr.229202.
40. Fan W. The chicken metagenome dataset from HiFi sequencing. AGIS website. 2022. ftp://ftp.agis.org.cn/~fanwei/Chicken_gut_metagenome_Hifi/.
41. Fan W, Zhang Y, Jiang F, Yang B, Wang S, Wang HC, Wang A, Xu D. Supporting data for "Improved microbial genomes and gene catalog of the chicken gut from metagenomic sequencing of high-fidelity long reads" GigaScience Database. 2022. http://dx.doi.org/10.5524/102330.

Tables

Table 1. Statistics of PacBio HiFi sequencing data

Intestinal compartment	PacBio Cell number	Number of reads	Number of bases (bp)	N50 read length (bp)	Median read quality (Phred)
**Duodenum**	1	2,734,871	22,233,516,165	9,778	39
**Jejunum**	2	2,669,321	44,559,115,216	16,417	35
**Ileum**	2	4,282,202	72,828,594,344	16,856	33
**Cecum**	3	5,045,925	80,959,163,166	17,319	31
**Colorectum**	3	5,865,946	111,891,321,947	19,258	31
**All**	11	20,598,265	332,471,710,838	17,316	32

Figures

Figure 1. Graphic display of the contig assembly graph. Random colors were chosen for different contigs. The line length is proportional to the contig length, and the line width is proportional to the contig coverage depth. Some examples for super complex, tangled circular, individual circular and linear contigs were labeled. This plot shows the colorectum assembly drawn by Bandage.

Figure 2. Contig assembly statistics. (a) Histogram of total assembled contig sizes for each intestinal compartment. (b) Histogram of N50 contig sizes for each intestinal compartment. (c) Correlation plot of contig length and coverage depth, generated using contig data from all intestinal compartments. The red marker line indicates that sufficient coverage depth contributes to contig continuity. (d) Correlation plot of the YAK quality score (QV) and coverage depth, using contigs with lengths over 100 kb from all intestinal compartments. The K-mer frequency was calculated with the parameters "yak count -b37 -t48" and the yak QV was calculated with the parameters "yak qv -t80 -p -K3.2g -l100k". The red marker line indicates that a higher coverage depth improves the single-base quality of the contig sequences.

Figure 3. Evaluation and ranking of assembled microbial genomes. (a) "Circular genomes" refers to circular contigs, and "non-circular MAGs" refers to incomplete genome assemblies derived from contig binning or merging algorithms. A circular genome or non-circular MAG is defined as 'near-complete' if its CheckM completeness is ≥90% and its contamination level ≤5%, defined as 'high-quality' if completeness ≥70% and contamination ≤10%, or defined as 'medium-quality' if completeness ≥50% and contamination ≤10%. Combined (NR) is the non-redundant set of microbial genomes from all intestinal compartments. All the microbial genomes in Combined (NR) have ≤

99% ANI to the other microbial genomes in Combined (NR). (b) Distribution of the assembled microbial genome sizes for circular genomes and non-circular MAGs. (c) Distribution of the CheckM scores (completeness – 5 * contamination) for circular genomes and non-circular MAGs.

Figure 4. Statistics of non-coding RNA genes in assembled microbial genomes. (a) Distribution of the number of full rRNA operons (i.e., those that encode 5S, 16S, and 23S rRNA). (b) Distribution of the number of tRNA genes.

Figure 5. Comparison of HiFi-assembled microbial genomes with short-read assembled MAGs. (a) Matching of our 461 assembled microbial strain-level genomes (99% average nucleotide identity, ANI) with 12,339 public dereplicated MAGs (99% ANI) derived from short-reads. The HiFi-assembled microbial genome was considered a match if its ANI was higher than 99% for any short-read assembled MAG. (b) Matching of our 337 assembled microbial species-level genomes (95% ANI) with 1,978 public dereplicated MAGs (95% ANI) derived from short-reads. The HiFi-assembled microbial genome was considered a match if its ANI was higher than 95% for any short-read assembled MAG. The unmatched microbial genomes unveil candidates of novel strains and species. (c) Number of genomes, (d) average contig number, (e) averaged assembled genome size, (f) average N50 contig size, (g) average CheckM completeness, (h) average CheckM contamination of the circular genomes, non-circular MAGs and public chicken gut MAGs assembled from short-reads.

Figure 6. Comparison of HiFi-derived gene catalog (HiFi-RGC) with two short-read derived gene catalogs (CGM-RGC and GG-IGC). CGM-RGC refers to chicken gut metagenome – reference gene catalog published by Huang et al. in 2018, GG-IGC refers to Gallus gallus – Integrated gene catalog published by Feng et al. in 2021. (a) Gene number and (b) gene structure completeness ratio of the 3 gene catalogs. Overlap of HiFi-RGC and CGM-RGC (c) and GG-IGC (d). A confident overlap is defined by the criteria of sequence identity ≥95% and length overlap ≥90% of the shorter sequence.

Figure 7. Phylogeny of the HiFi-assembled microbial genomes. Each colored clade corresponds to a phylum inferred by GTDB-Tk. Inside the largest phylum Firmicutes, five genera *Ligilactobacillus*, *Limosilactobacillus*, *Lactobacillus*, *Weissella*, and *Enterococcus* are also colored for highlighting. The leaf nodes of the phylogenetic tree have two shapes: a solid circle represents a circular genome, and a hollow circle represents a non-circular MAG. The colors of the leaf nodes represent CheckM quality ranks: green represents Near-complete assemblies, blue represents High-quality assemblies, and red represents Medium-quality assemblies. The inner ring shows the GTDB-Tk classification, and a triangle indicates that the corresponding leaf node matches an existing genome in the GTDB database. The five outer rings show the sequencing coverage depth for each assembled microbial genome from each intestinal compartment. From inner to outer: duodenum, jejunum, ileum, cecum, and colorectum.

Figure 1

Click here to access/download;Figure;Figure_1.Bandage view of Rectum.pdf

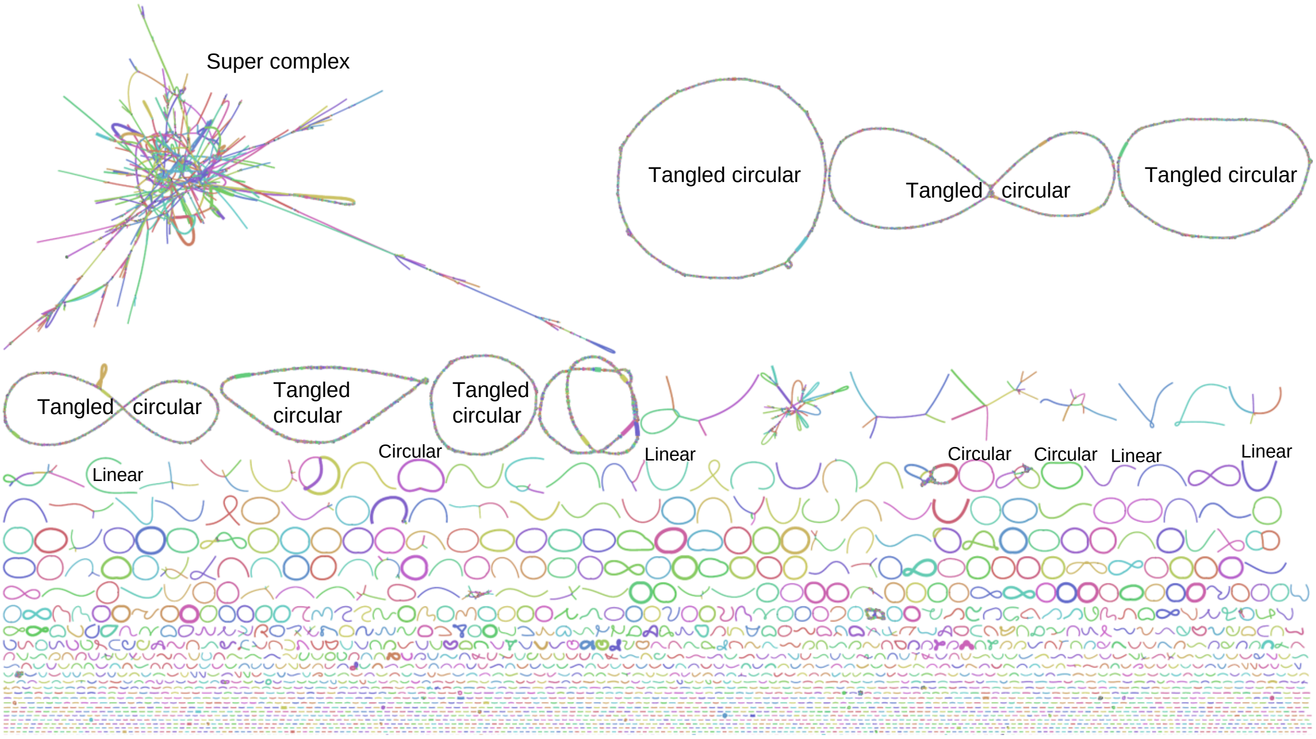

Figure 2

Click here to access/download;Figure;Figure_2.Statistics of assembly.pdf

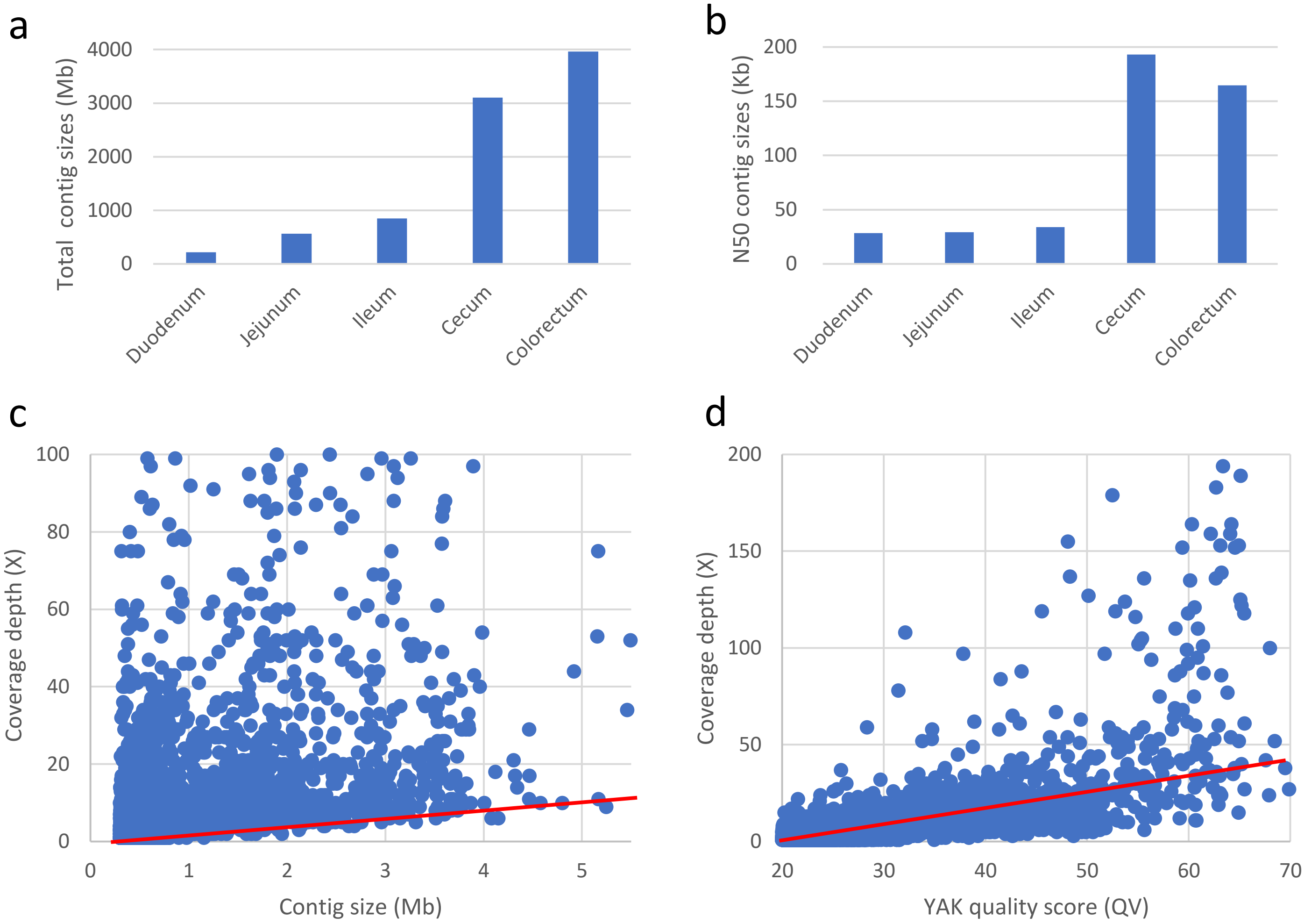

Figure 3

Click here to access/download;Figure;Figure_3.Statistics of genomes.pdf

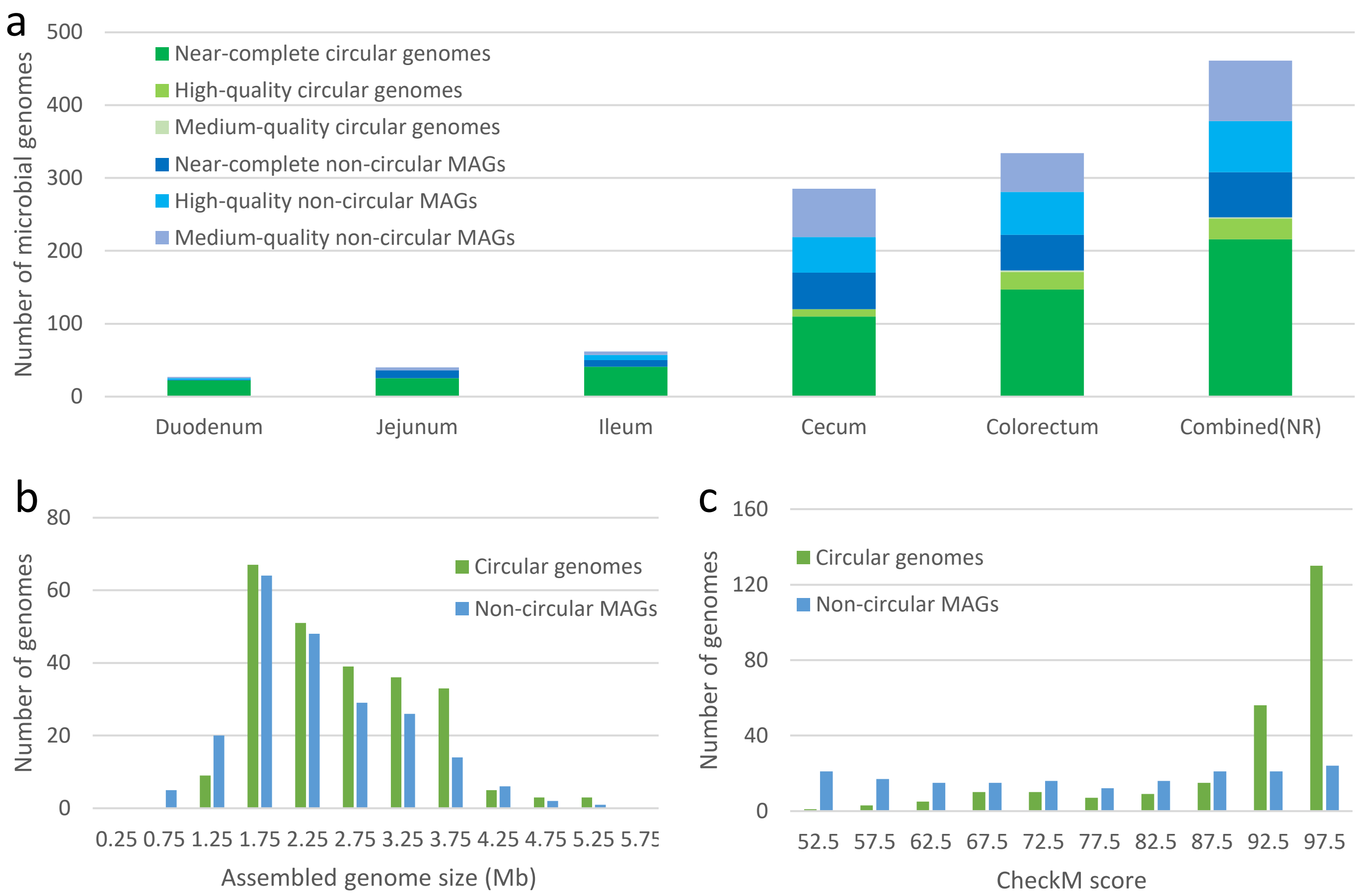

Figure 4

Click here to access/download;Figure;Figure_4.Statistics of rRNA and tRNA.pdf

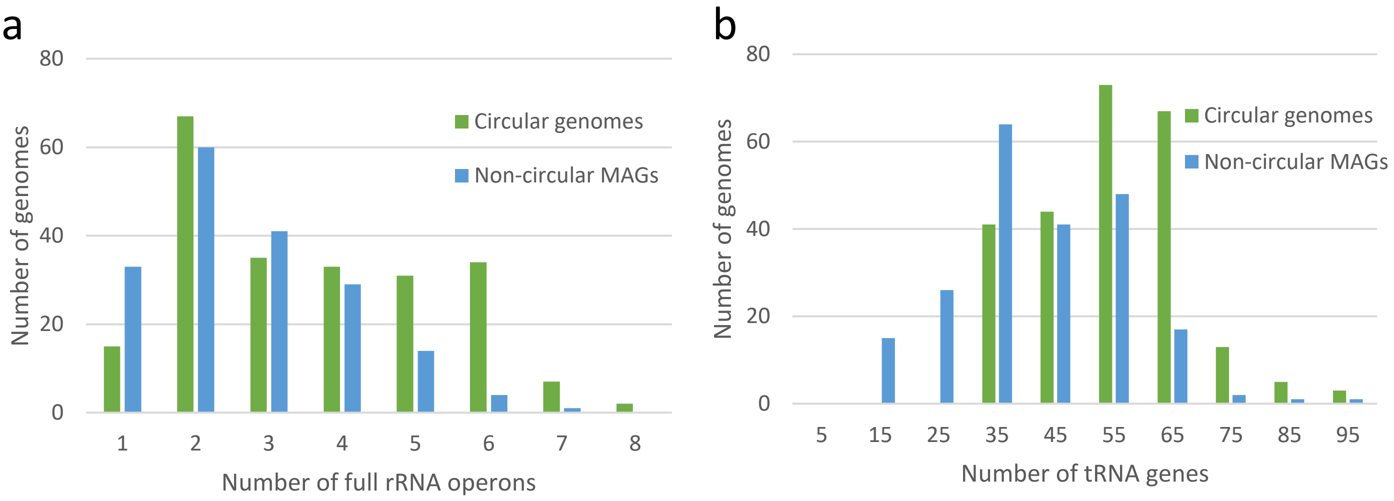

Figure 5

Click here to access/download;Figure;Figure_5.Comparison_of_MAG

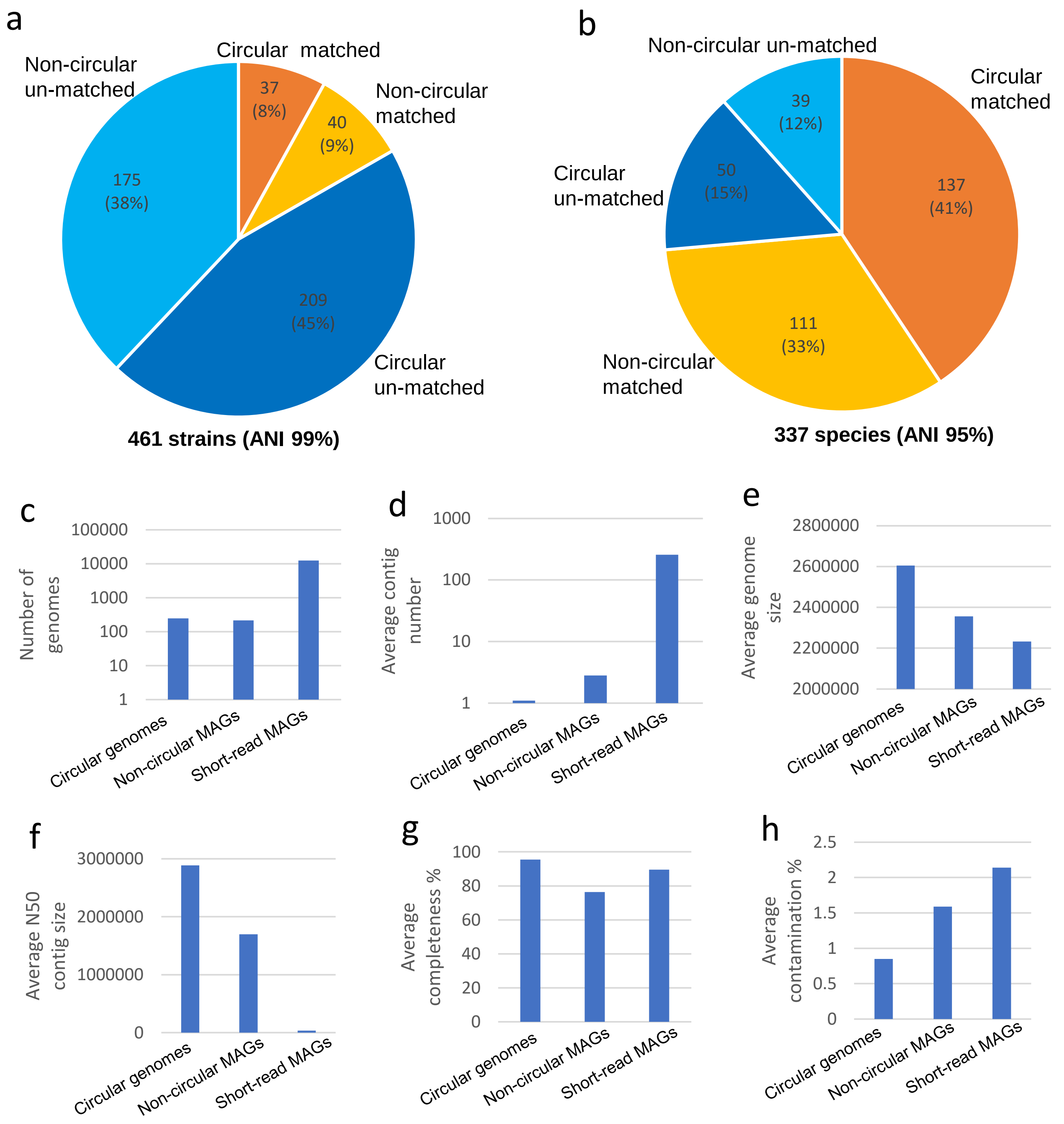

Figure 6

Click here to access/download;Figure;Figure_6.Comparion of gene catalogs2.pdf

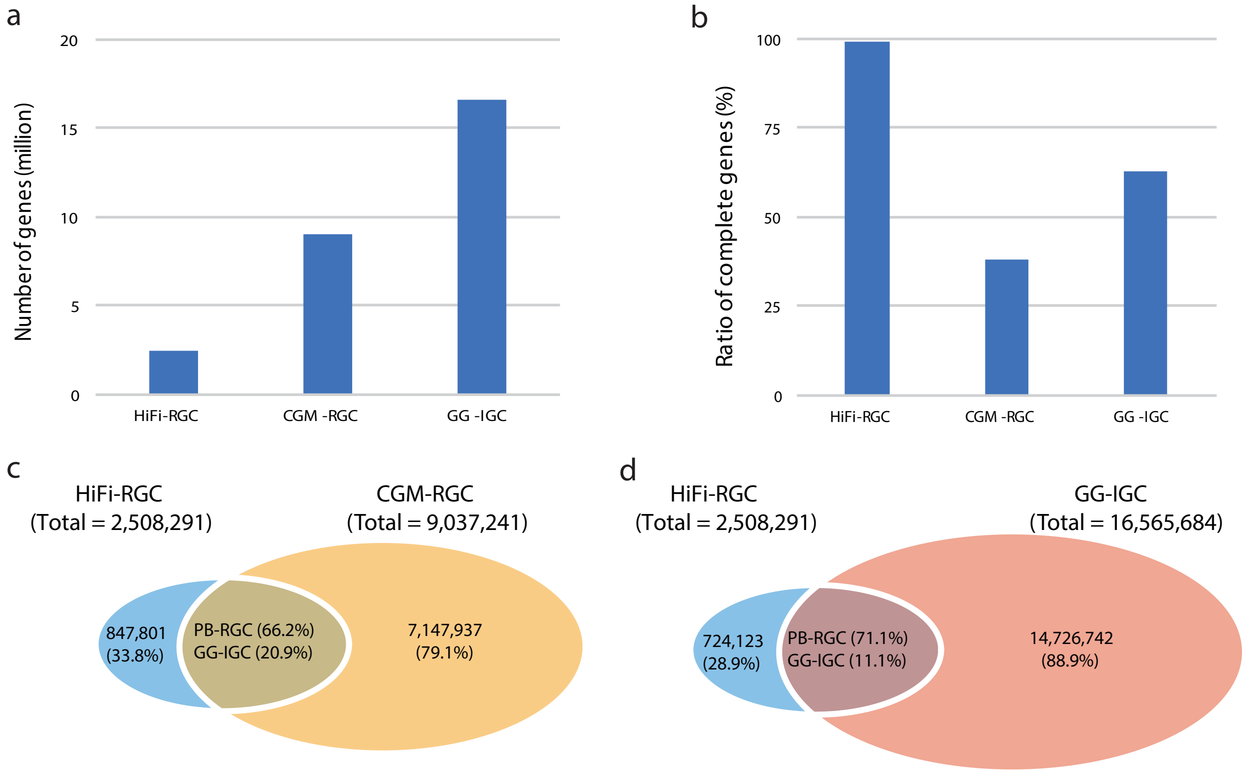

Figure 7

Click here to access/download;Figure;Figure_7.NR1.pdf

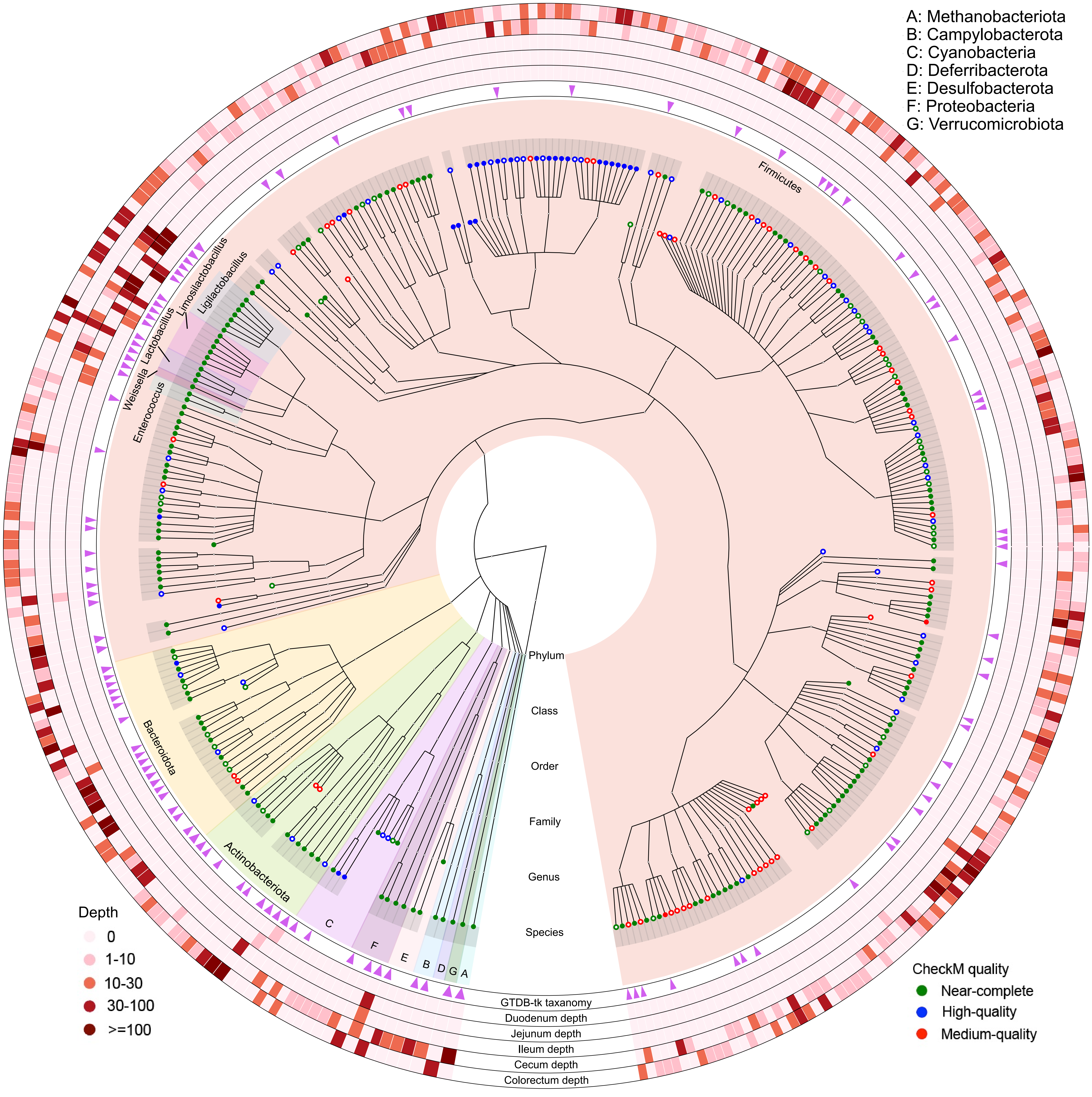

Click here to access/download
Supplementary Material
Supplemental_Revison1_20220920.docx
