# Supplementary material for: Improved microbial genomes and gene catalog of the chicken gut from metagenomic sequencing of high-fidelity long reads
Source: Gigascience. 2022 Nov 18;11:giac116. doi: 10.1093/gigascience/giac116 (PMC9673493; doi:10.1093/gigascience/giac116)
Supplement: giac116_Supplemental_File [file giac116_supplemental_file.docx]

## Supporting Information for

**Improved microbial genomes and gene catalog of chicken gut from metagenomic sequencing of** **high-fidelity long reads**

Yan Zhang*, Fan Jiang*, Boyuan Yang*, Sen Wang, Hengchao Wang, Anqi Wang, Dong Xu, and Wei Fan

Guangdong Laboratory for Lingnan Modern Agriculture (Shenzhen Branch), Genome Analysis Laboratory of the Ministry of Agriculture and Rural Affairs, Agricultural Genomics Institute at Shenzhen, Chinese Academy of Agricultural Sciences, Shenzhen, Guangdong, 518120, China.

*These authors contributed equally to this work. Correspondence should be addressed to [fanwei@caas.cn](mailto:fanwei@caas.cn).

## Supplementary figures


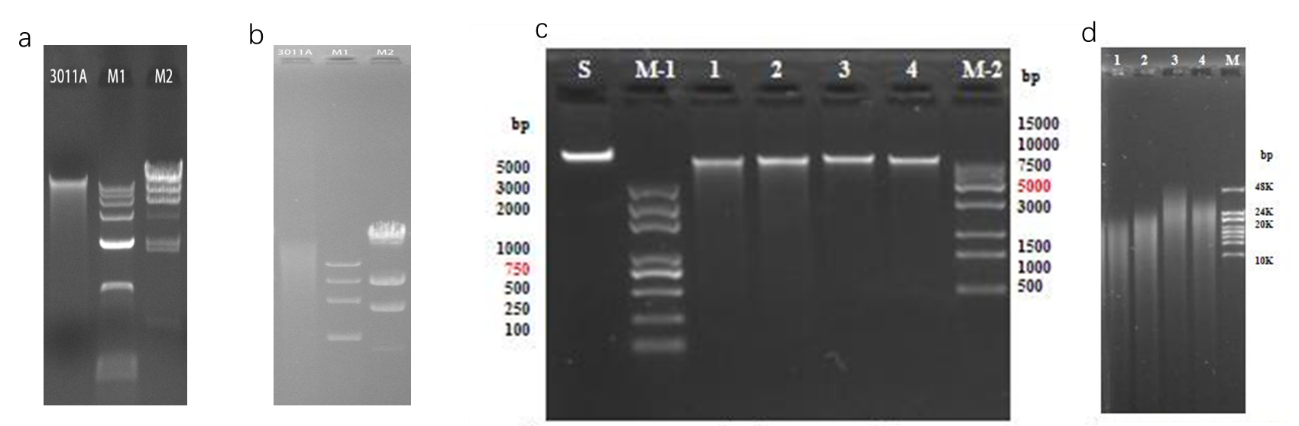


**Figure S1**. Agarose gel electrophoresis. (a) DC electrophoresis (0.7% gel, 100V, 1h) for Duodenum microbiota DNA (3011A); M1 15kb DNA Marker(15000、10000、7500、5000、2500、1000、250bp); M2 λDNA /HindIII(23130、9416、6557、4361、2322、2027、564bp). (b) Pulse electrophoresis (0.7% gel, pulse 5~80kb, 16h) for Duodenum microbiota DNA (3011A); M1 15kb DNA Marker; M2 λDNA /HindIII; (c) DC electrophoresis (1% gel, 180V, 20min) for Jejunum microbiota DNA (lane 1), Ileum microbiota DNA (lane 2), Cecum microbiota DNA (lane 3), and Colorectum microbiota DNA (lane 4); S stantard sample (50ng); M-1 trans 2k plus; M-2 trans 15k plus. (d) Pulse electrophoresis (0.8% gel, pulse 5~80kb, 17h) for Jejunum microbiota DNA (lane 1), Ileum microbiota DNA (lane 2), Cecum microbiota DNA (lane 3), and Colorectum microbiota DNA (lane 4); M 48kb DNA Extension Ladder. In summary, the microbiota DNA from all intestinal fragments are intact except for the Duodenum, which is slightly degraded. The microbiota DNA from all intestinal fragments are qualified for HiFi sequencing.


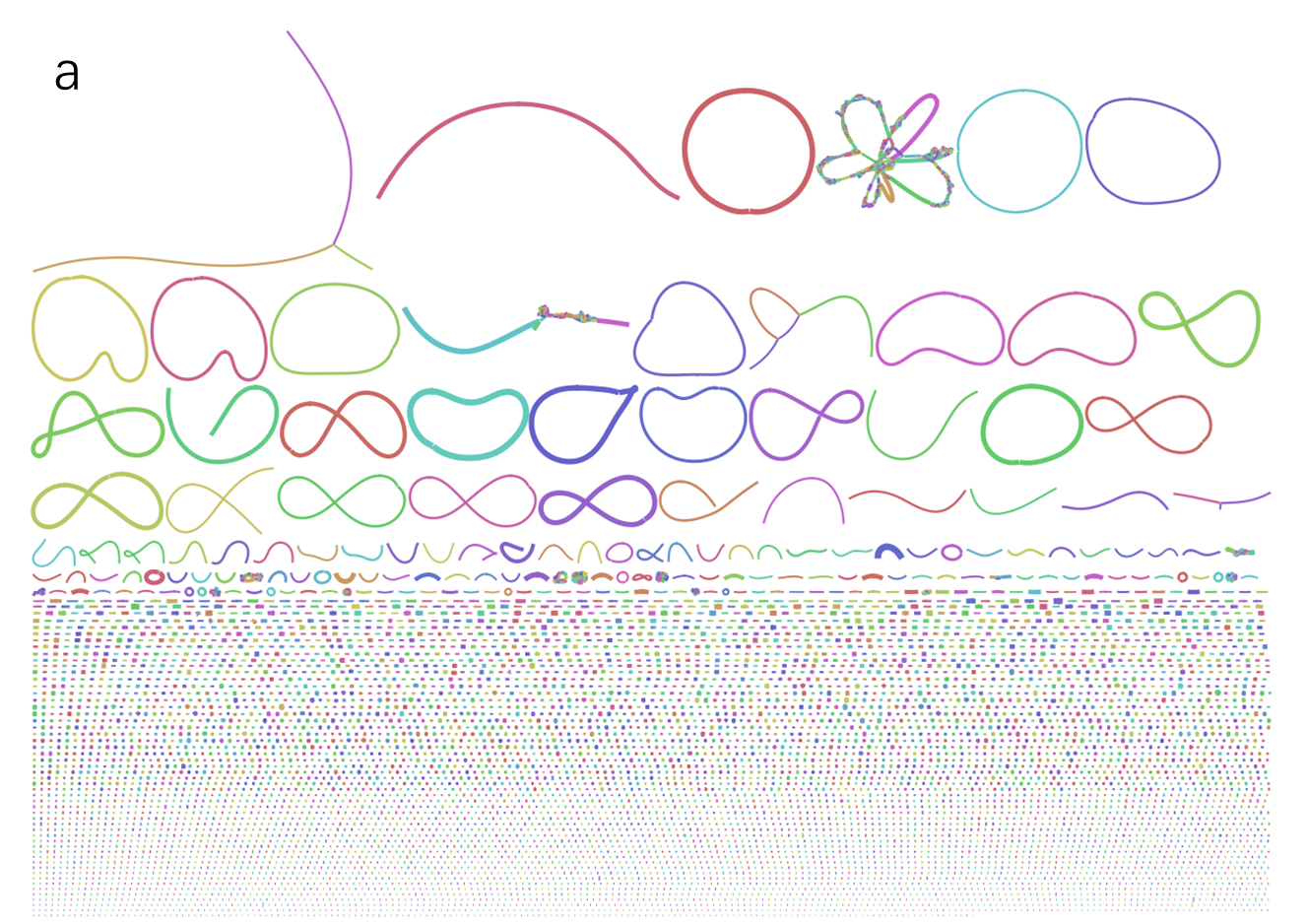


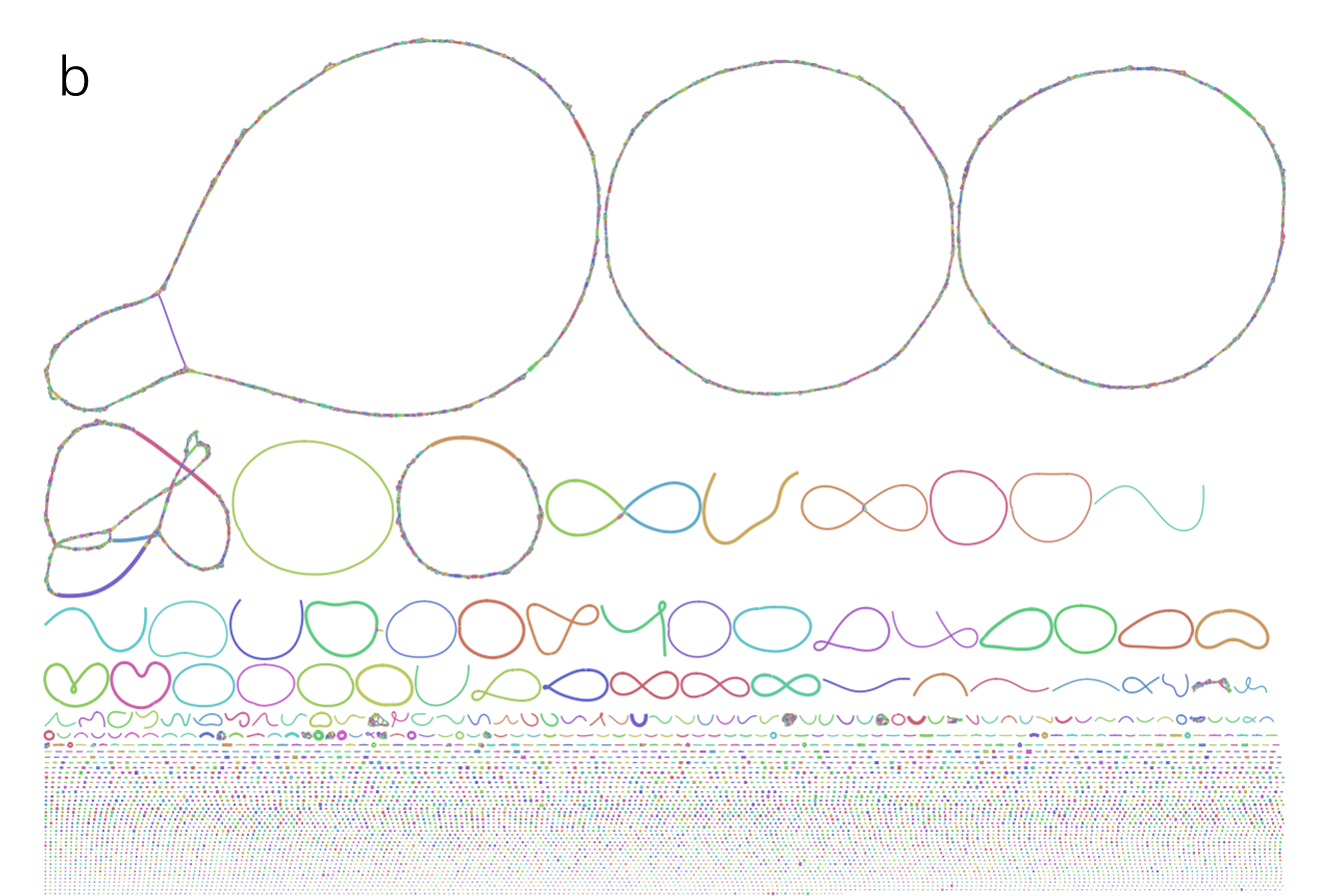


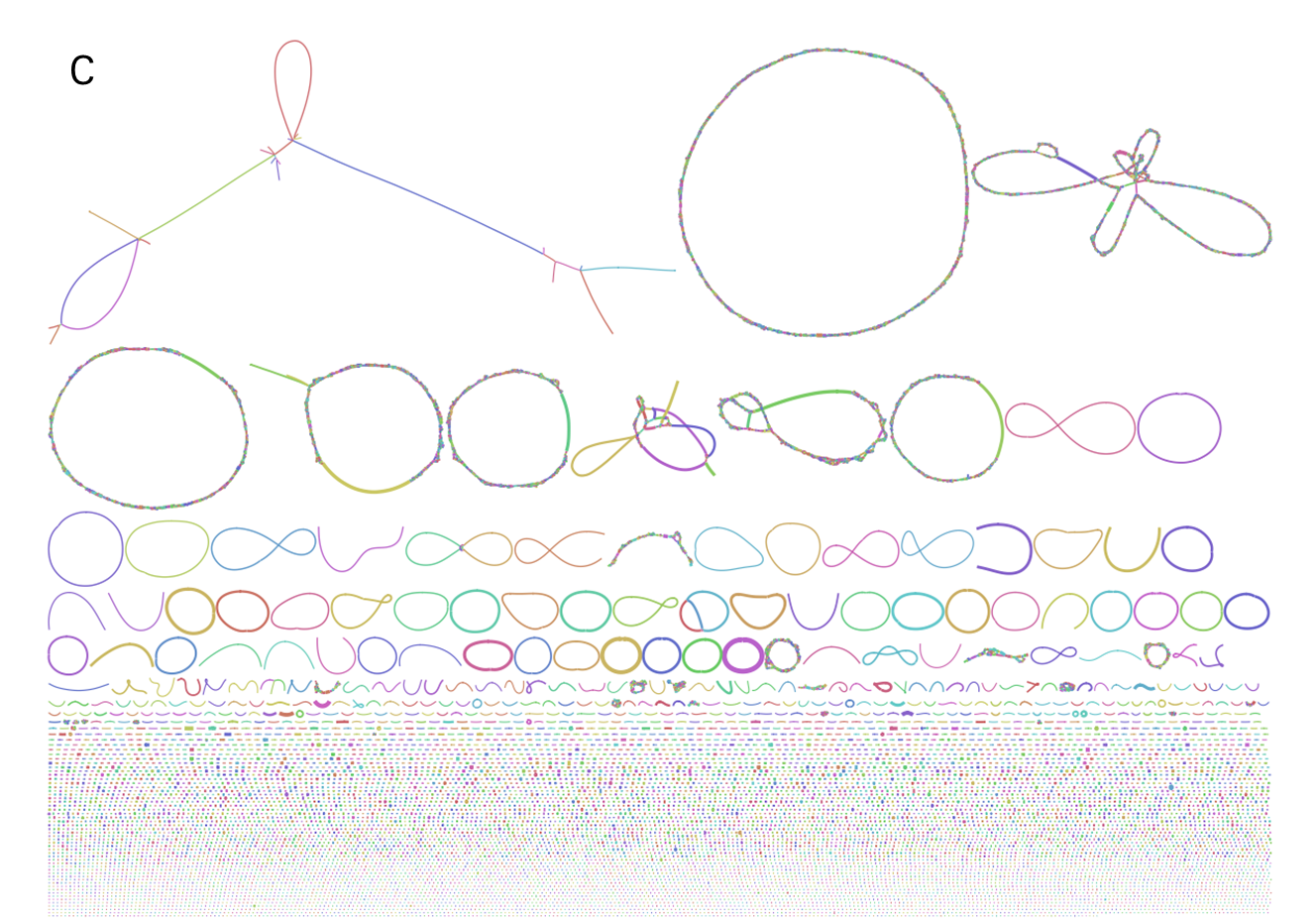


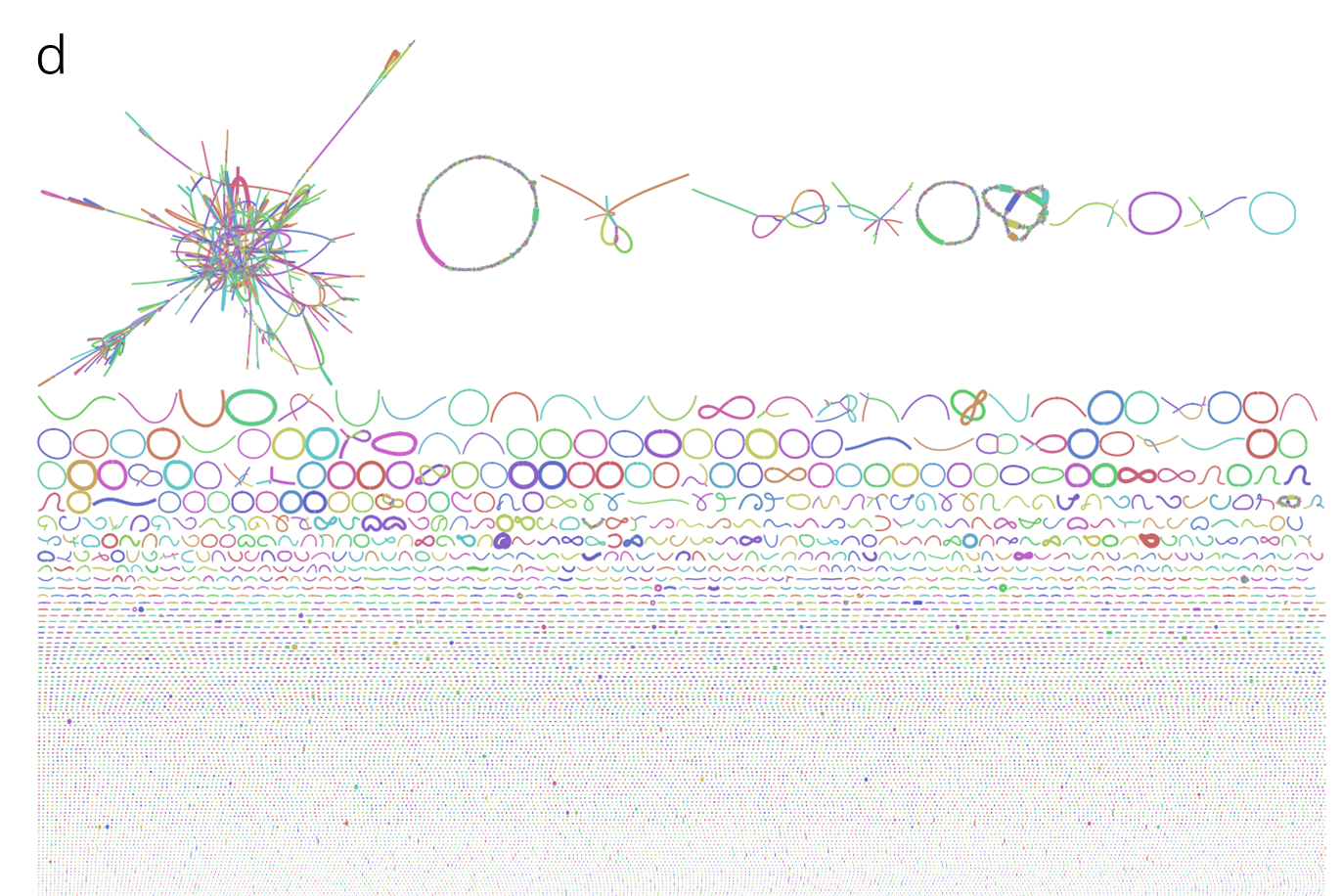


**Figure S2**. Graphic display of the contig assembly graphs for (a) Duodenum, (b) Jejunum, (c) Ileum and (d) Cecum. Random colors were chosen for different contigs. The line length is in proportion to contig length, and the line width is in proportion to contig coverage depth. These plots are drawn by Bandage, with the same style to Figure 1 in the maintext.

**Figure S3**. Correlation plot of contig length and coverage depth for each intestinal fragment: duodenum, jejunum, ileum, cecum, and colorectum. The average coverage depth for a contig is calculated from the reads data that used to assemble this contig. The plots have the same style to Figure 2c in the maintext.

**Figure S4.** Correlation plot of assembled genome size and checkM score (completeness – 5 * contamination) for each intestinal fragment: duodenum, jejunum, ileum, cecum, and colorectum.


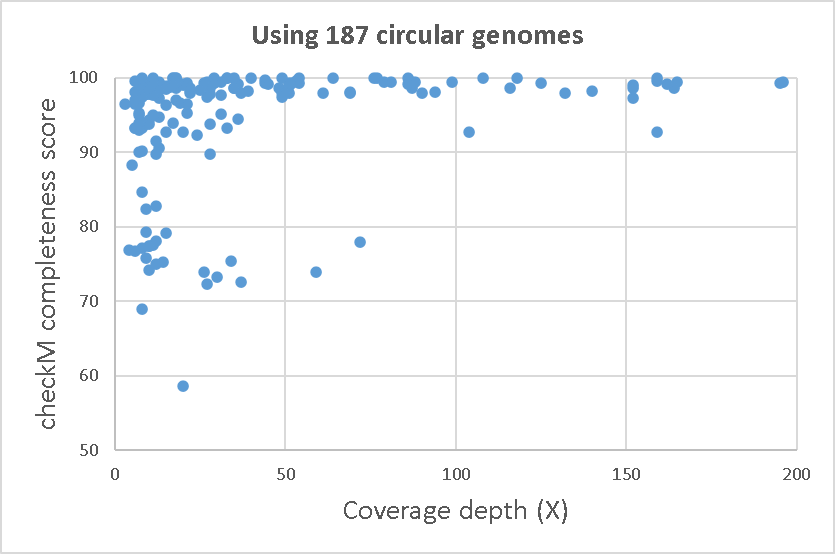


**Figure S5.** Correlation plot of genome coverage depth and checkM completeness score. The 187 circular genomes out of 337 non-redundant species-level genomes were used here. Considering all these genomes have complete genome assemblies, the difference of checkM completeness scores should only be caused by the single base accuracy, due to the marker gene prediction method adopted by checkM. Genome assemblies with higher single base accuracy will have higher checkM completeness values. The plots clearly shows that higher coverage depth will result in higher checkM completeness scores, indicating that higher coverage depth will improve the single base accuracy of genome assemblies.

**Figure S6**. Comparison of microbial composition at phylum level between the unique (28.9%) and the shared (71.1%) parts of genes in HiFi-RGC, which was determined by comparison to GG-IGC. The “Others” contains the phyla with the ratio of genes less than 0.5%. Unclassified means these genes haven’t been successfully classified to the phylum level.


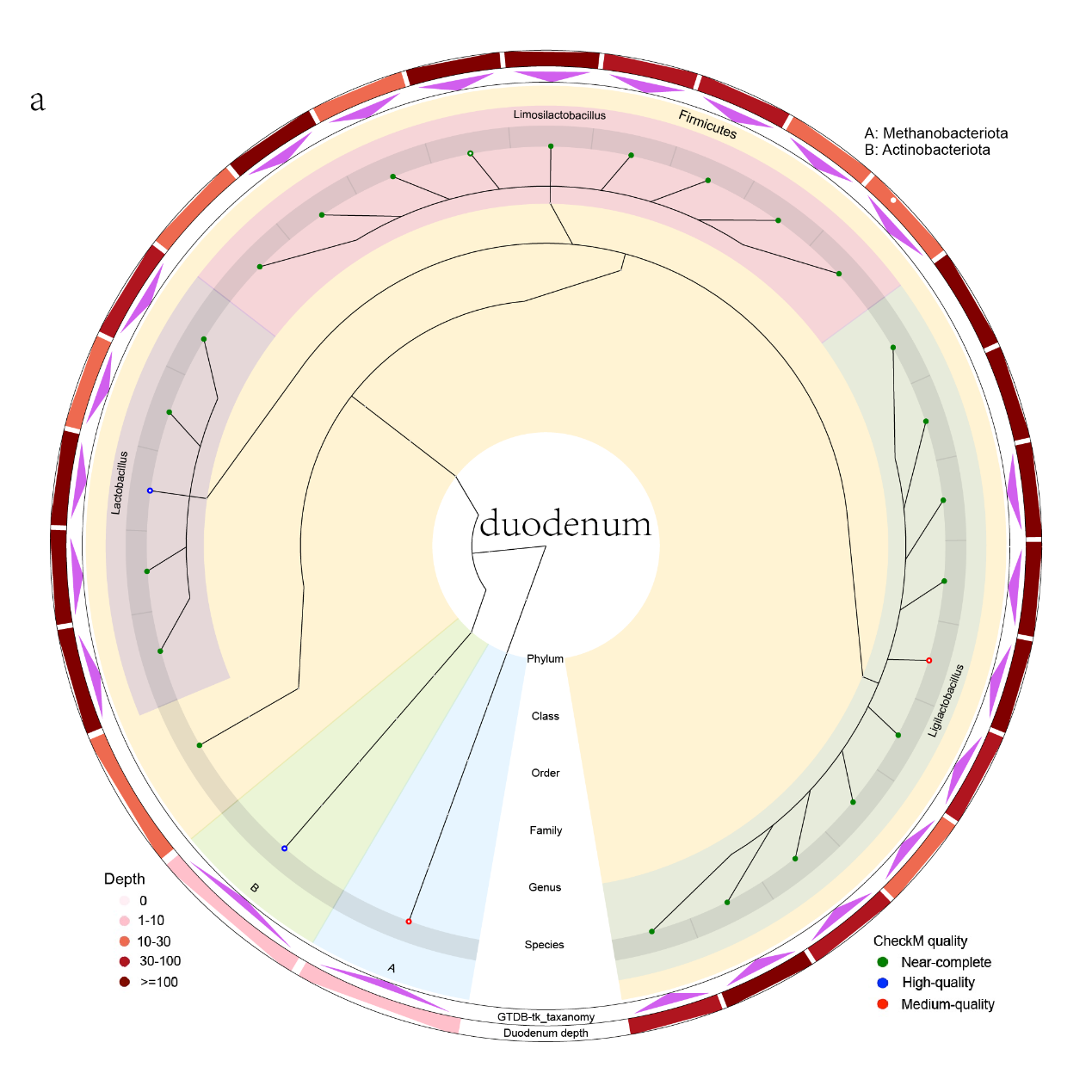


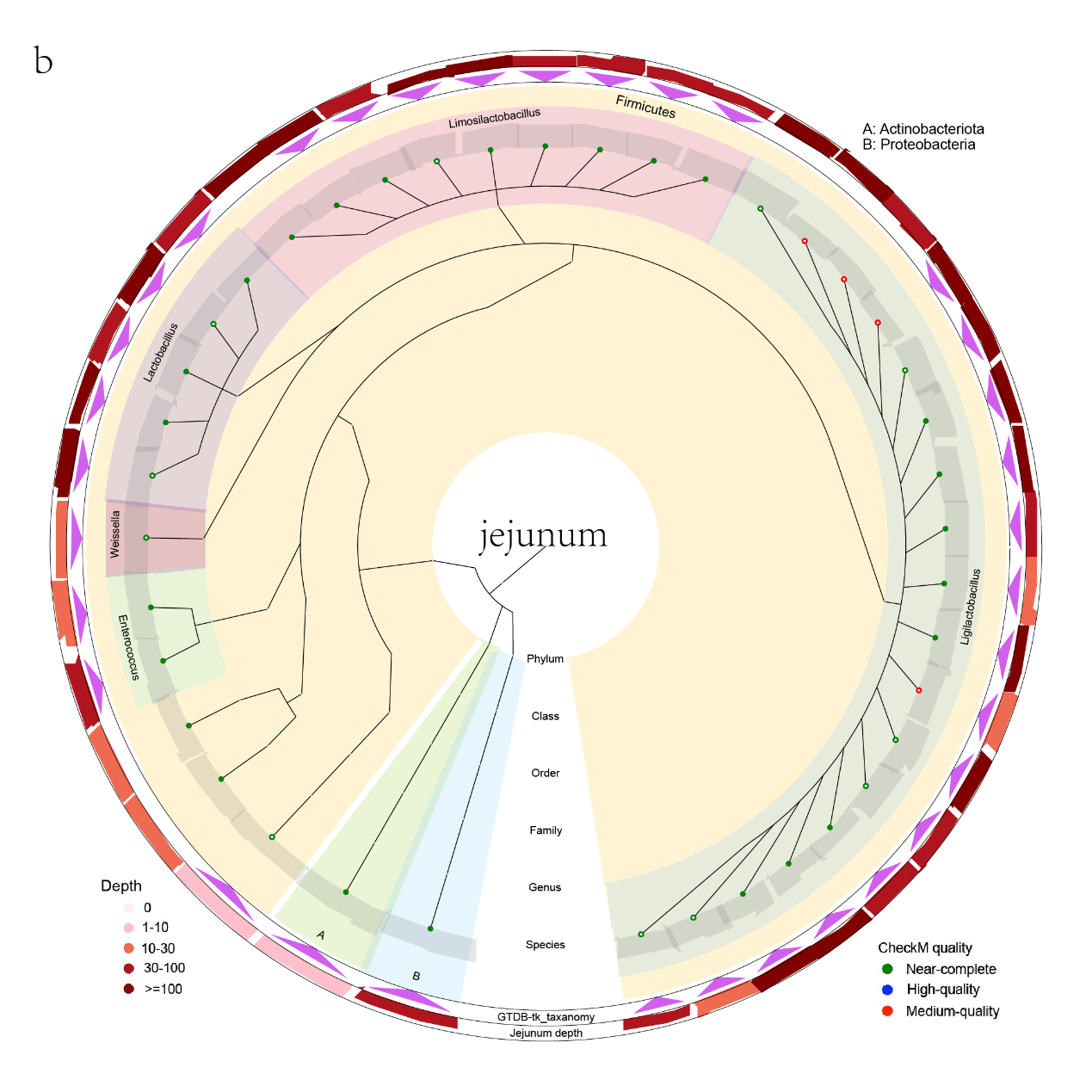


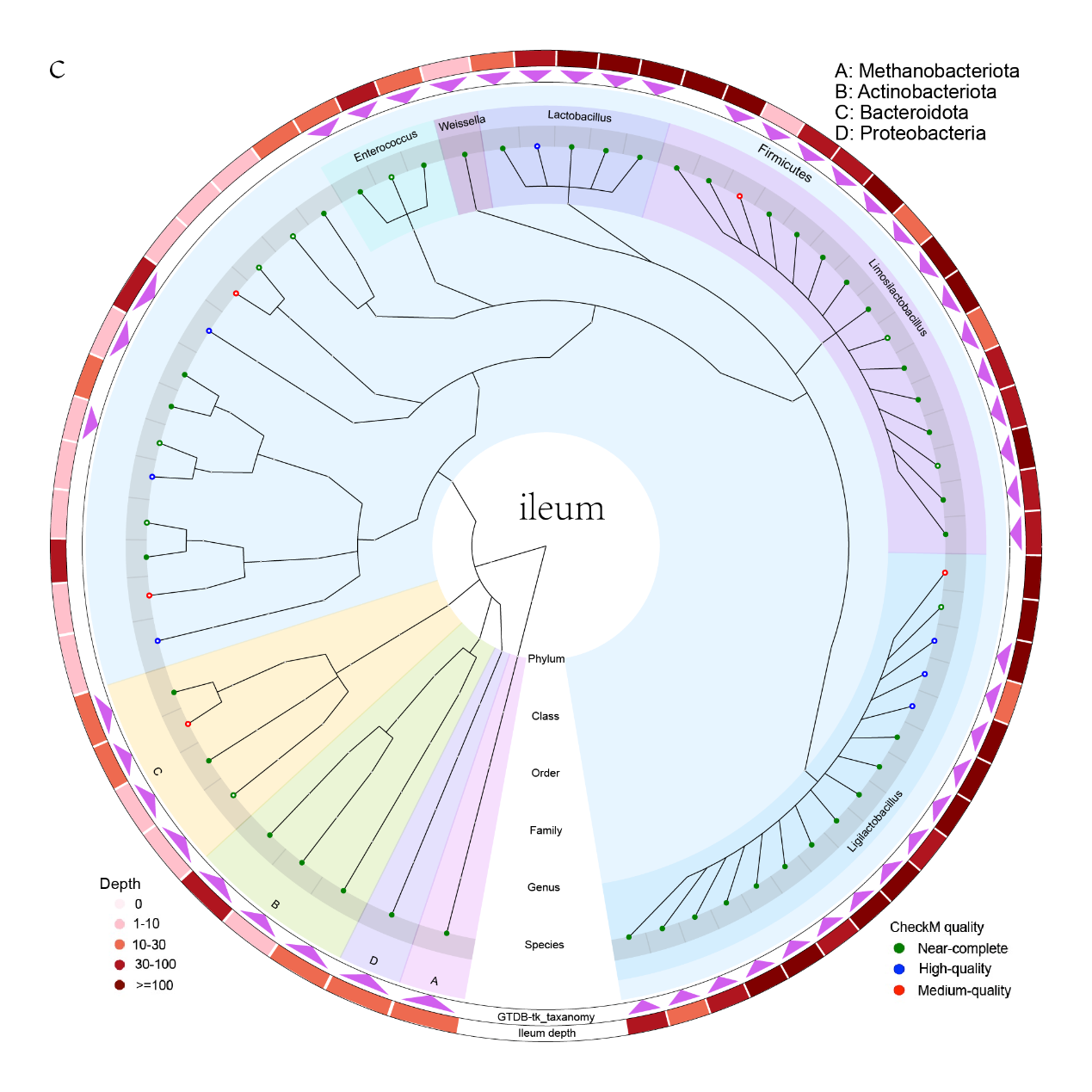


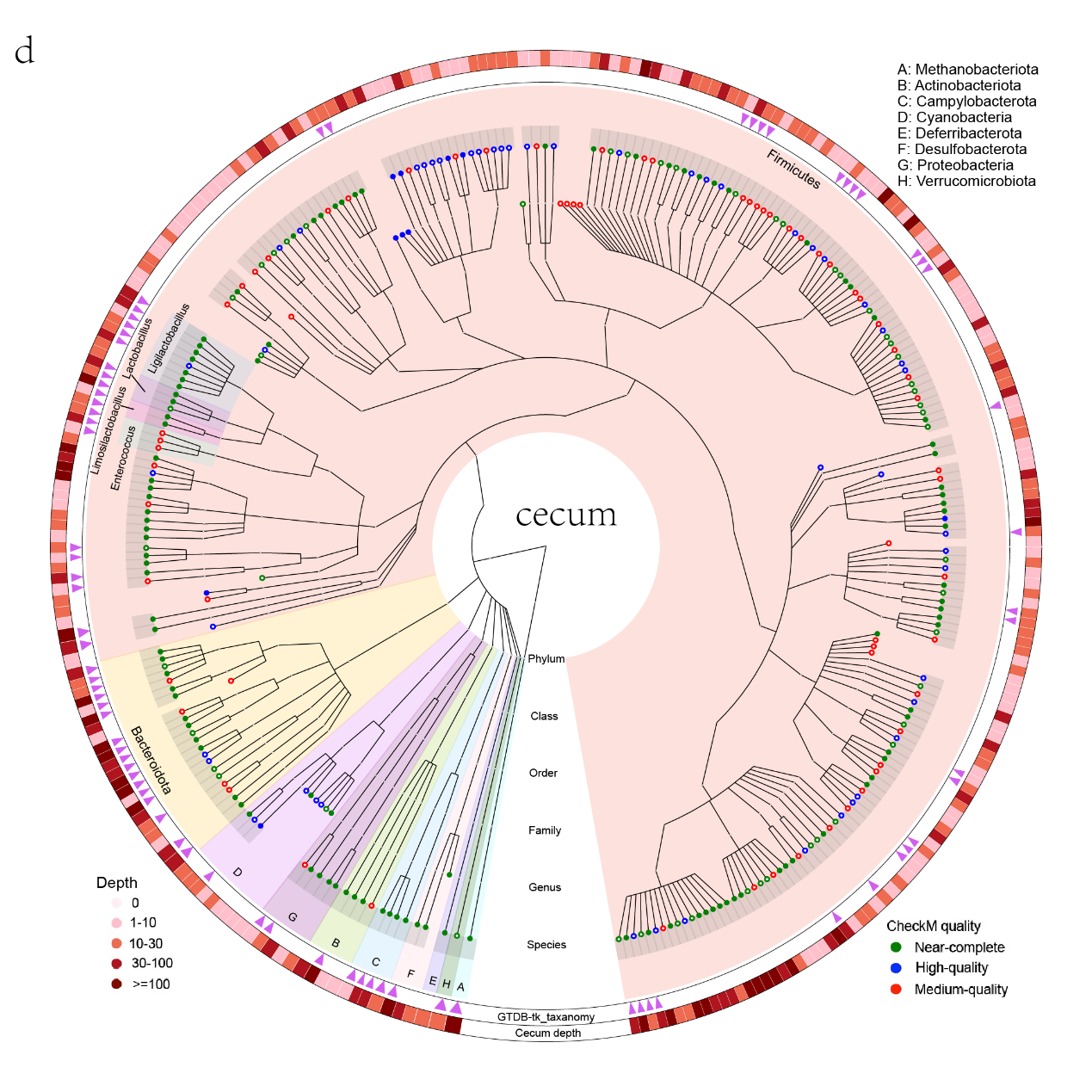


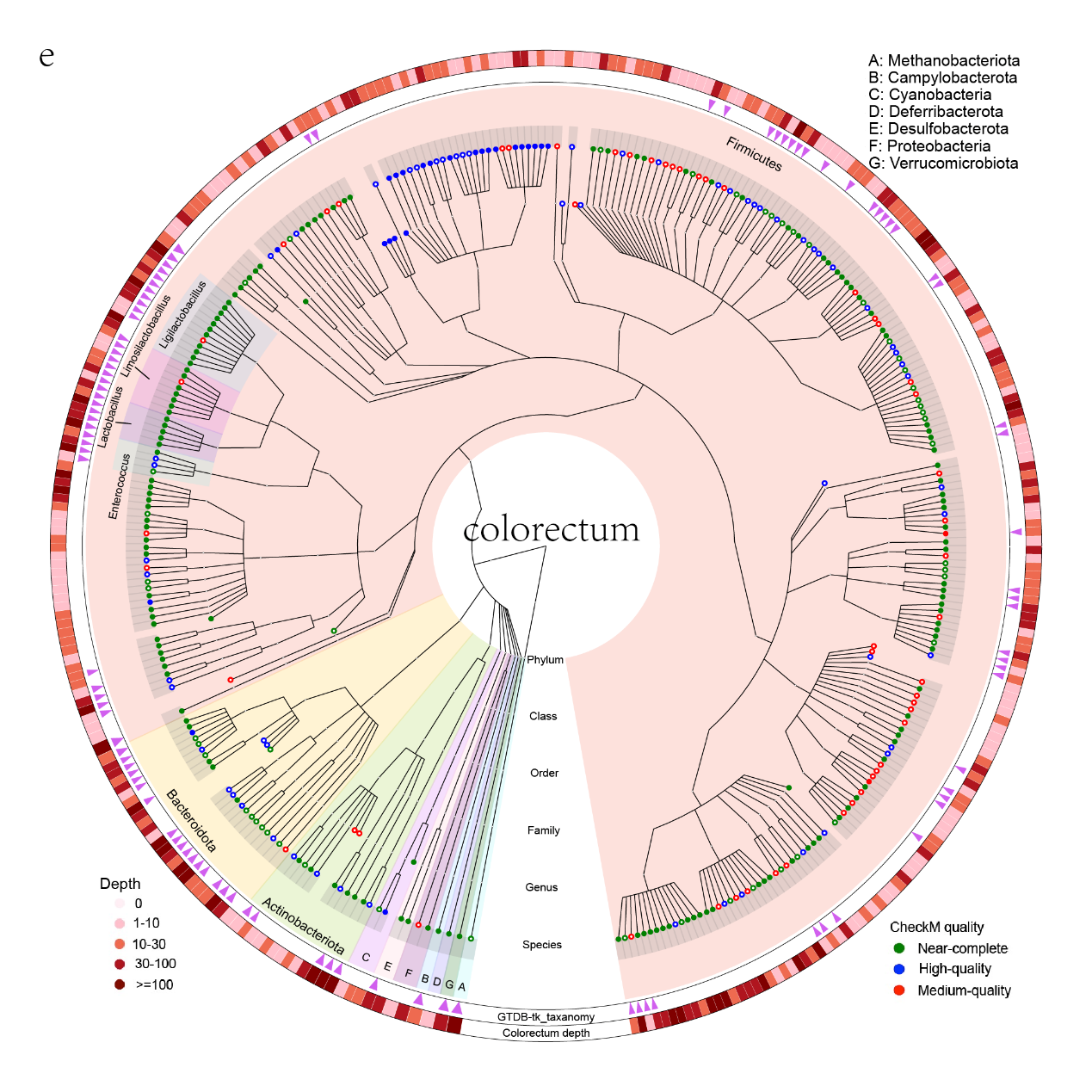


**Figure S7.** Phylogeny of the HiFi-assembled microbial genomes (strain-level) for each intestinal fragment: duodenum, jejunum, ileum, cecum, and colorectum. A colored clade corresponds to a phylum inferred by GTDB-Tk. Inside the largest phylum Firmicutes, five genus *Ligilactobacillus*, *Limosilactobacillus*, *Lactobacillus*, *Weissella*, and *Enterococcus* are also colored for highlighting. The leaf nodes of the phylogenic tree have two shapes: “solid circle” represents circular genome, “hollow circle” represents non-circular MAG. The colors of the leaf nodes represent checkM quality ranks: “green” refer to near-complete, “blue” refer to “High-quality”, and “red” refer to “Medium-quality”. The inner ring shows GTDB-tk classification, and a triangle means the corresponding leaf node is matched to a known species in the GTDB database. The outer ring shows the sequencing coverage depth for each assembled microbial genome. The plots are in similar style to Figure 7 in the maintext.

## Supplementary tables

**Table S1**. Quality and Quantity assessment of the extracted DNA

| **Megagenomic DNA samples** | **Volume（μl）** | **Qubit 4** | | **Nanodrop 2000c** | | | |
| --- | --- | --- | --- | --- | --- | --- | --- |
|  |  | **Quantity (ug)** | **Concentration (ng/μl)** | **Quantity (ug)** | **Concentration (ng/μl)** | **A260/A280** | **A260/A230** |
| Duodenum | 60 | 3.66 | 61 | 4.62 | 77 | 1.91 | 2.19 |
| Jejunum | 120 | 13.68 | 114 | 16.92 | 141 | 1.86 | 2.04 |
| Ileum | 120 | 27.36 | 228 | 31.68 | 264 | 1.86 | 2.21 |
| Cecum | 120 | 83.52 | 696 | 84.72 | 706 | 1.87 | 2.18 |
| Colorectum | 120 | 56.16 | 468 | 70.80 | 590 | 1.87 | 2.15 |

**Table S2**. Statistics of contig sizes for the five intestinal compartments

|  | Duodenum | Jejunum | Ileum | Cecum | Colorectum |
| --- | --- | --- | --- | --- | --- |
| Total | 215416290 | 563,926,391 | 845,725,856 | 3,106,222,594 | 3,964,544,903 |
| Maximum | 2140764 | 4,919,211 | 4,800,555 | 5,494,600 | 5,461,226 |
| N10 | 1863229 | 1,736,722 | 1,925,074 | 2,765,591 | 2,817,978 |
| N20 | 1579685 | 116,408 | 391,356 | 1,594,200 | 1,719,359 |
| N30 | 239141 | 35,584 | 79,867 | 798,017 | 812,687 |
| N40 | 43464 | 31,340 | 40,681 | 379,965 | 328,036 |
| N50 | 28358 | 29,093 | 33,983 | 193,062 | 164,639 |
| N60 | 23877 | 26,576 | 30,425 | 112,641 | 89,617 |
| N70 | 19048 | 21,647 | 25,980 | 74,185 | 56,280 |
| N80 | 13459 | 19,909 | 21,348 | 52,711 | 38,723 |
| N90 | 9445 | 17,053 | 18,263 | 35,852 | 28,794 |
| Minimum | 2045 | 7,254 | 6,876 | 2,317 | 6,962 |

**Table S3**. Number of microbial genomes for each quality rank

| Quality rank | Duodenum | Jejunum | Ileum | Cecum | Colorectum | Combined(NR) |
| --- | --- | --- | --- | --- | --- | --- |
| Near-complete circular contigs | 22 | 25 | 41 | 110 | 147 | 216 |
| High-quality circular contigs | 0 | 0 | 0 | 10 | 24 | 28 |
| Medium-quality circular contigs | 0 | 0 | 0 | 0 | 2 | 2 |
| Near-complete tangled "circular" assembly | 0 | 2 | 1 | 0 | 2 | 0 |
| High-quality tangled "circular" assembly | 0 | 0 | 1 | 0 | 5 | 0 |
| Medium-quality tangled "circular" assembly | 0 | 1 | 1 | 1 | 0 | 0 |
| Near-complete linear binning MAGs | 1 | 9 | 8 | 50 | 47 | 62 |
| High-quality linear binning MAGs | 2 | 0 | 6 | 49 | 54 | 70 |
| Medium-quality linear binning MAGs | 2 | 3 | 4 | 65 | 53 | 83 |
| Total | 27 | 40 | 62 | 285 | 334 | 461 |

Note: this table is a supplementary to Figure 3a. Circular contigs refer to complete microbial genome assembly directly obtained from Hifiasm-meta, tangled "circular" assembly refer to re-assembly (merging) of the tangled “circular” that is composed of many tiny fragments, and linear binning MAGs are derived from MetaBAT2 contig binning of the incomplete linear contigs. In Figure 3a, circular contigs are classified as circular genomes, while tangled "circular" assembly and linear binning MAGs are classified into non-circular MAGs.

**Table S4**. Statistics of assembled circular plasmid and viral genomes

| Types | Duodenum | Jejunum | Ileum | Cecum | Colorectum |
| --- | --- | --- | --- | --- | --- |
| Plasmid | 47 | 65 | 65 | 62 | 67 |
| Uncertain - plasmid or chromosomal | 14 | 2 | 6 | 19 | 11 |
| Virus | 5 | 6 | 8 | 38 | 45 |
| Uncertain - viral or bacterial | 28 | 8 | 6 | 14 | 5 |
| Total | 94 | 81 | 85 | 133 | 128 |

Note: these numbers are counted from viralVerify result files (*_result_table.csv).

**Table S5**. Unclassified number of genomes at each taxonomic level

|  | GTDB-tk | GTDB-tk + RDP | GTDB-tk + RDP + Silva |
| --- | --- | --- | --- |
| Family level | 2 | 1 | 0 |
| Genus level | 35 | 21 | 9 |
| Species level | 189 | 189 | 49 |

Note: Here the 337 species-level microbial genomes were used as input, and the unclassified number of genomes at each taxonomic levels (family, genus, species) were shown by three classification methods: (1) GTDB-tk alone; (2) GTDB-tk and Ribosomal Database Project (RDP) Classifier; (3) GTDB-tk and Ribosomal Database Project (RDP) Classifier and alignments to the Silva 16S rRNA database. The last method classified the maximum number of genomes, and only 9 and 49 genomes failed to be classified into known genus and species, indicating that they may be novel genus and species which haven’t been reported before.

**Table S6**. Nine inferred novel genus by GTDB-tk + RDP + Silva method

| Microbial genome_ID | Compare to short-read MAGs | GTDB-tk taxonomic classification | RDP classification with 16S rRNA | Identity (Silva) | Blast score (Silva) | 16S rRNA best hit to the Silva database |
| --- | --- | --- | --- | --- | --- | --- |
| Cecum.out.p_ctg.gfa.seq.fa.len.circular.fa.035 | Un-matched | d__Bacteria;p__Desulfobacterota;c__Desulfovibrionia;o__Desulfovibrionales;f__Desulfovibrionaceae;g__;s__ | d__Bacteria(100%);p__Proteobacteria(100%);c__Deltaproteobacteria(100%);o__Desulfovibrionales(100%);f__Desulfovibrionaceae(100%);g__Desulfovibrio(53%) | 89.987 | 1067 | Bacteria;Desulfobacterota;Desulfovibrionia;Desulfovibrionales;Desulfovibrionaceae;Desulfovibrio;Desulfovibrio sp. 3_1_syn3 |
| Cecum.out.p_ctg.gfa.seq.fa.len.circular.fa.120 | Un-matched | d__Bacteria;p__Firmicutes_A;c__Clostridia;o__TANB77;f__CAG-508;g__;s__ | d__Bacteria(100%);p__Firmicutes(100%);c__Clostridia(98%);o__Clostridiales(98%);f__Ruminococcaceae(97%);g__Hydrogeniiclostridium(21%) | 94.471 | 1232 | Bacteria;Firmicutes;Clostridia;Clostridia UCG-014;uncultured bacterium |
| Cecum.out.p_ctg.gfa.seq.fa.len.linear.fa.bin.684 | Matched | d__Bacteria;p__Firmicutes_A;c__Clostridia;o__Lachnospirales;f__Lachnospiraceae;g__;s__ | d__Bacteria(100%);p__Firmicutes(100%);c__Clostridia(100%);o__Clostridiales(100%);f__Lachnospiraceae(100%);g__Bariatricus(30%) | 93.921 | 1220 | Bacteria;Firmicutes;Clostridia;Lachnospirales;Lachnospiraceae;[Ruminococcus] torques group;uncultured bacterium |
| Cecum.out.p_ctg.gfa.seq.fa.len.linear.fa.bin.734 | Matched | d__Bacteria;p__Firmicutes;c__Bacilli;o__Bacillales;f__;g__;s__ | d__Bacteria(100%);p__Firmicutes(100%);c__Bacilli(99%);o__Bacillales(99%);f__Bacillaceae 2(51%);g__Salirhabdus(31%) | 92.759 | 1191 | Bacteria;Firmicutes;Bacilli;Bacillales;Bacillaceae;Bacillus;Streptococcus pneumoniae |
| Cecum.out.p_ctg.gfa.seq.fa.len.linear.fa.bin.773 | Matched | d__Bacteria;p__Firmicutes_A;c__Clostridia;o__Oscillospirales;f__Oscillospiraceae;g__;s__ | d__Bacteria(100%);p__Firmicutes(100%);c__Clostridia(100%);o__Clostridiales(100%);f__Ruminococcaceae(100%);g__Intestinimonas(69%) | 92.163 | 1132 | Bacteria;Firmicutes;Clostridia;Oscillospirales;Oscillospiraceae;Papillibacter;uncultured Firmicutes bacterium |
| Cecum.out.p_ctg.gfa.seq.fa.len.linear.fa.bin.999 | Matched | d__Bacteria;p__Firmicutes_A;c__Clostridia;o__Lachnospirales;f__Lachnospiraceae;g__;s__ | d__Bacteria(100%);p__Firmicutes(100%);c__Clostridia(100%);o__Clostridiales(100%);f__Lachnospiraceae(100%);g__Clostridium XlVa(34%) | 93.956 | 1217 | Bacteria;Firmicutes;Clostridia;Lachnospirales;Lachnospiraceae;Marvinbryantia;uncultured bacterium |
| Rectum.out.p_ctg.gfa.seq.fa.len.circular.fa.112 | Matched | d__Bacteria;p__Firmicutes;c__Bacilli;o__RF39;f__UBA660;g__;s__ | d__Bacteria(100%);p__Firmicutes(75%);c__Erysipelotrichia(62%);o__Erysipelotrichales(62%);f__Erysipelotrichaceae(62%);g__Amedibacillus(7%) | 92.298 | 1183 | Bacteria;Firmicutes;Bacilli;RF39;uncultured rumen bacterium |
| Rectum.out.p_ctg.gfa.seq.fa.len.linear.fa.bin.1509 | Un-matched | d__Bacteria;p__Actinobacteriota;c__Coriobacteriia;o__Coriobacteriales;f__Eggerthellaceae;g__;s__ | d__Bacteria(100%);p__Actinobacteria(100%);c__Coriobacteriia(100%);o__Eggerthellales(98%);f__Eggerthellaceae(98%);g__Slackia(42%) | 94.055 | 1223 | Bacteria;Actinobacteriota;Coriobacteriia;Coriobacteriales;Eggerthellaceae;CHKCI002;Coriobacteriaceae bacterium CHKCI002 |
| Rectum.out.p_ctg.gfa.seq.fa.len.linear.fa.bin.512 | Matched | d__Bacteria;p__Bacteroidota;c__Bacteroidia;o__Bacteroidales;f__Rikenellaceae;g__;s__ | d__Bacteria(100%);p__Bacteroidetes(100%);c__Bacteroidia(100%);o__Bacteroidales(100%);f__Rikenellaceae(98%);g__Mucinivorans(51%) | 91.237 | 1094 | Bacteria;Bacteroidota;Bacteroidia;Bacteroidales;Rikenellaceae;Rikenella;uncultured bacterium |

Table S7. Forty-nine inferred novel species by GTDB-tk + RDP + Silva method

| Microbial genome_ID | Compare to short-read MAGs | GTDB-tk taxonomic classification | RDP classification with 16S rRNA | Identity (Silva) | Blast score (Silva) | 16S rRNA best hit to the Silva database |
| --- | --- | --- | --- | --- | --- | --- |
| Cecum.out.p_ctg.gfa.seq.fa.len.circular.fa.060 | Un-matched | d__Bacteria;p__Firmicutes_A;c__Clostridia;o__Lachnospirales;f__Lachnospiraceae;g__UBA7182;s__ | d__Bacteria(100%);p__Firmicutes(100%);c__Clostridia(100%);o__Clostridiales(100%);f__Lachnospiraceae(100%);g__Clostridium XlVa(59%) | 94.385 | 1240 | Bacteria;Firmicutes;Clostridia;Lachnospirales;Lachnospiraceae;uncultured;uncultured bacterium |
| Cecum.out.p_ctg.gfa.seq.fa.len.circular.fa.090 | Un-matched | d__Bacteria;p__Firmicutes_A;c__Clostridia;o__Lachnospirales;f__Lachnospiraceae;g__Merdimonas;s__ | d__Bacteria(100%);p__Firmicutes(100%);c__Clostridia(100%);o__Clostridiales(100%);f__Lachnospiraceae(100%);g__Mordavella(95%) | 96.264 | 1336 | Bacteria;Firmicutes;Clostridia;Lachnospirales;Lachnospiraceae;Lachnoclostridium;Mordavella massiliensis |
| Cecum.out.p_ctg.gfa.seq.fa.len.circular.fa.098 | Matched | d__Bacteria;p__Bacteroidota;c__Bacteroidia;o__Bacteroidales;f__Rikenellaceae;g__Tidjanibacter;s__ | d__Bacteria(100%);p__Bacteroidetes(100%);c__Bacteroidia(100%);o__Bacteroidales(100%);f__Rikenellaceae(100%);g__Alistipes(98%) | 93.842 | 1216 | Bacteria;Bacteroidota;Bacteroidia;Bacteroidales;Rikenellaceae;Alistipes;uncultured bacterium |
| Cecum.out.p_ctg.gfa.seq.fa.len.circular.fa.113 | Matched | d__Bacteria;p__Proteobacteria;c__Alphaproteobacteria;o__RF32;f__CAG-239;g__CAG-495;s__ | d__Bacteria(100%);p__Proteobacteria(99%);c__Alphaproteobacteria(98%);o__Kiloniellales(58%);f__Kiloniellaceae(58%);g__Aestuariispira(36%) | 94.213 | 1225 | Bacteria;Proteobacteria;Alphaproteobacteria;Rhodospirillales;uncultured;uncultured bacterium |
| Cecum.out.p_ctg.gfa.seq.fa.len.circular.fa.128 | Un-matched | d__Bacteria;p__Firmicutes_A;c__Clostridia;o__Lachnospirales;f__Lachnospiraceae;g__Mediterraneibacter;s__ | d__Bacteria(100%);p__Firmicutes(100%);c__Clostridia(100%);o__Clostridiales(100%);f__Lachnospiraceae(100%);g__Mediterraneibacter(98%) | 96.365 | 1348 | Bacteria;Firmicutes;Clostridia;Lachnospirales;Lachnospiraceae;[Ruminococcus] torques group;Clostridium sp. Marseille-P3244 |
| Cecum.out.p_ctg.gfa.seq.fa.len.linear.fa.bin.1016 | Matched | d__Bacteria;p__Firmicutes_A;c__Clostridia;o__Oscillospirales;f__Ruminococcaceae;g__Anaerotruncus;s__ | d__Bacteria(100%);p__Firmicutes(100%);c__Clostridia(100%);o__Clostridiales(100%);f__Ruminococcaceae(100%);g__Anaerotruncus(100%) | 96.68 | 1355 | Bacteria;Firmicutes;Clostridia;Oscillospirales;Ruminococcaceae;Anaerotruncus;Clostridiales bacterium VE202-13 |
| Cecum.out.p_ctg.gfa.seq.fa.len.linear.fa.bin.1120 | Un-matched | d__Bacteria;p__Firmicutes_A;c__Clostridia;o__Lachnospirales;f__Lachnospiraceae;g__Enterocloster;s__ | d__Bacteria(100%);p__Firmicutes(100%);c__Clostridia(100%);o__Clostridiales(100%);f__Lachnospiraceae(100%);g__Enterocloster(60%) | 96.669 | 1322 | Bacteria;Firmicutes;Clostridia;Lachnospirales;Lachnospiraceae;uncultured;uncultured bacterium |
| Cecum.out.p_ctg.gfa.seq.fa.len.linear.fa.bin.1253 | Un-matched | d__Bacteria;p__Firmicutes_A;c__Clostridia;o__Oscillospirales;f__Acutalibacteraceae;g__CAG-557;s__ | d__Bacteria(100%);p__Firmicutes(100%);c__Clostridia(100%);o__Clostridiales(100%);f__Ruminococcaceae(100%);g__Clostridium IV(71%) | 93.767 | 1218 | Bacteria;Firmicutes;Clostridia;Oscillospirales;Ruminococcaceae;uncultured;uncultured bacterium |
| Cecum.out.p_ctg.gfa.seq.fa.len.linear.fa.bin.336 | Matched | d__Bacteria;p__Firmicutes_A;c__Clostridia_A;o__Christensenellales;f__UBA3700;g__CABKMX01;s__ | d__Bacteria(100%);p__Firmicutes(100%);c__Clostridia(96%);o__Clostridiales(96%);f__Catabacteriaceae(17%);g__Catabacter(17%) | 94.482 | 1224 | Bacteria;Firmicutes;Clostridia;Clostridia vadinBB60 group;uncultured bacterium |
| Cecum.out.p_ctg.gfa.seq.fa.len.linear.fa.bin.635 | Matched | d__Bacteria;p__Firmicutes_A;c__Clostridia;o__Oscillospirales;f__Oscillospiraceae;g__UBA9475;s__ | d__Bacteria(100%);p__Firmicutes(100%);c__Clostridia(100%);o__Clostridiales(100%);f__Ruminococcaceae(100%);g__Intestinimonas(44%) | 96.522 | 1335 | Bacteria;Firmicutes;Clostridia;Oscillospirales;Oscillospiraceae;uncultured;uncultured bacterium |
| Cecum.out.p_ctg.gfa.seq.fa.len.linear.fa.bin.664 | Matched | d__Bacteria;p__Firmicutes_A;c__Clostridia;o__Lachnospirales;f__Lachnospiraceae;g__Blautia_A;s__ | d__Bacteria(100%);p__Firmicutes(100%);c__Clostridia(100%);o__Clostridiales(100%);f__Lachnospiraceae(100%);g__Blautia(100%) | 95.971 | 1330 | Bacteria;Firmicutes;Clostridia;Lachnospirales;Lachnospiraceae;Blautia;uncultured bacterium |
| Cecum.out.p_ctg.gfa.seq.fa.len.linear.fa.bin.982 | Matched | d__Bacteria;p__Firmicutes_A;c__Clostridia;o__Lachnospirales;f__Lachnospiraceae;g__Blautia_A;s__ | d__Bacteria(100%);p__Firmicutes(100%);c__Clostridia(100%);o__Clostridiales(100%);f__Lachnospiraceae(100%);g__Blautia(36%) | 92.923 | 1187 | Bacteria;Firmicutes;Clostridia;Lachnospirales;Lachnospiraceae;Blautia;Ruminococcus sp. 5_1_39BFAA |
| Rectum.out.p_ctg.gfa.seq.fa.len.circular.fa.005 | Matched | d__Bacteria;p__Firmicutes_A;c__Clostridia;o__Monoglobales_A;f__UBA1381;g__12844;s__ | d__Bacteria(100%);p__Firmicutes(100%);c__Clostridia(100%);o__Clostridiales(100%);f__Ruminococcaceae(100%);g__Monoglobus(88%) | 96.713 | 1365 | Bacteria;Firmicutes;Clostridia;Monoglobales;Monoglobaceae;Monoglobus;uncultured bacterium |
| Rectum.out.p_ctg.gfa.seq.fa.len.circular.fa.024 | Un-matched | d__Bacteria;p__Firmicutes;c__Bacilli;o__Erysipelotrichales;f__Erysipelatoclostridiaceae;g__CHKCI006;s__ | d__Bacteria(100%);p__Firmicutes(100%);c__Erysipelotrichia(100%);o__Erysipelotrichales(100%);f__Erysipelotrichaceae(92%);g__Coprobacillus(22%) | 95.409 | 1271 | Bacteria;Firmicutes;Bacilli;Erysipelotrichales;Erysipelatoclostridiaceae;Erysipelatoclostridium;uncultured bacterium |
| Rectum.out.p_ctg.gfa.seq.fa.len.circular.fa.025 | Matched | d__Bacteria;p__Firmicutes_A;c__Clostridia_A;o__Christensenellales;f__CAG-74;g__SFFH01;s__ | d__Bacteria(100%);p__Firmicutes(100%);c__Clostridia(98%);o__Clostridiales(98%);f__Ruminococcaceae(53%);g__Pseudoclostridium(37%) | 96.62 | 1354 | Bacteria;Firmicutes;Clostridia;Christensenellales;Christensenellaceae;Christensenellaceae R-7 group;uncultured bacterium |
| Rectum.out.p_ctg.gfa.seq.fa.len.circular.fa.036 | Matched | d__Bacteria;p__Cyanobacteria;c__Vampirovibrionia;o__Gastranaerophilales;f__Gastranaerophilaceae;g__CAG-484;s__ | d__Bacteria(100%);p__Proteobacteria(78%);c__Deltaproteobacteria(78%);o__Bdellovibrionales(78%);f__Bdellovibrionaceae(78%);g__Vampirovibrio(78%) | 95.216 | 1287 | Bacteria;Cyanobacteria;Vampirivibrionia;Gastranaerophilales;uncultured bacterium |
| Rectum.out.p_ctg.gfa.seq.fa.len.circular.fa.044 | Matched | d__Bacteria;p__Firmicutes_A;c__Clostridia;o__Peptostreptococcales;f__Anaerovoracaceae;g__UBA1191;s__ | d__Bacteria(100%);p__Firmicutes(100%);c__Clostridia(100%);o__Clostridiales(100%);f__Clostridiales_Incertae Sedis XIII(93%);g__Ihubacter(56%) | 95.087 | 1259 | Bacteria;Firmicutes;Clostridia;Peptostreptococcales-Tissierellales;Anaerovoracaceae;Family XIII AD3011 group;uncultured bacterium |
| Rectum.out.p_ctg.gfa.seq.fa.len.circular.fa.047 | Un-matched | d__Bacteria;p__Firmicutes;c__Bacilli;o__RF39;f__UBA660;g__UMGS2016;s__ | d__Bacteria(100%);p__Firmicutes(73%);c__Erysipelotrichia(58%);o__Erysipelotrichales(58%);f__Erysipelotrichaceae(58%);g__Amedibacterium(21%) | 92.561 | 1195 | Bacteria;Firmicutes;Bacilli;RF39;uncultured rumen bacterium |
| Rectum.out.p_ctg.gfa.seq.fa.len.circular.fa.054 | Matched | d__Bacteria;p__Firmicutes_A;c__Clostridia;o__Oscillospirales;f__Ruminococcaceae;g__UMGS966;s__ | d__Bacteria(100%);p__Firmicutes(100%);c__Clostridia(100%);o__Clostridiales(100%);f__Ruminococcaceae(100%);g__Phocea(36%) | 95.853 | 1306 | Bacteria;Firmicutes;Clostridia;Oscillospirales;Ruminococcaceae;uncultured;uncultured bacterium |
| Rectum.out.p_ctg.gfa.seq.fa.len.circular.fa.057 | Matched | d__Bacteria;p__Firmicutes_A;c__Clostridia;o__TANB77;f__CAG-508;g__UMGS1994;s__ | d__Bacteria(100%);p__Firmicutes(100%);c__Clostridia(100%);o__Clostridiales(100%);f__Ruminococcaceae(100%);g__Hydrogeniiclostridium(29%) | 96.742 | 1354 | Bacteria;Firmicutes;Clostridia;Clostridia UCG-014;uncultured bacterium |
| Rectum.out.p_ctg.gfa.seq.fa.len.circular.fa.069 | Un-matched | d__Bacteria;p__Firmicutes_A;c__Clostridia;o__TANB77;f__CAG-508;g__CAG-273;s__ | d__Bacteria(100%);p__Firmicutes(100%);c__Clostridia(100%);o__Clostridiales(100%);f__Ruminococcaceae(97%);g__Petroclostridium(40%) | 96.939 | 1361 | Bacteria;Firmicutes;Clostridia;Clostridia UCG-014;uncultured bacterium |
| Rectum.out.p_ctg.gfa.seq.fa.len.circular.fa.117 | Matched | d__Bacteria;p__Firmicutes_A;c__Clostridia;o__Monoglobales_A;f__UBA1381;g__12844;s__ | d__Bacteria(100%);p__Firmicutes(100%);c__Clostridia(100%);o__Clostridiales(100%);f__Ruminococcaceae(100%);g__Monoglobus(53%) | 95.135 | 1294 | Bacteria;Firmicutes;Clostridia;Monoglobales;Monoglobaceae;Monoglobus;uncultured bacterium |
| Rectum.out.p_ctg.gfa.seq.fa.len.circular.fa.127 | Un-matched | d__Bacteria;p__Firmicutes;c__Bacilli;o__RF39;f__UBA660;g__UMGS2068;s__ | d__Bacteria(100%);p__Firmicutes(81%);c__Erysipelotrichia(78%);o__Erysipelotrichales(78%);f__Erysipelotrichaceae(78%);g__Erysipelotrichaceae_incertae_sedis(41%) | 93.511 | 1240 | Bacteria;Firmicutes;Bacilli;RF39;gut metagenome |
| Rectum.out.p_ctg.gfa.seq.fa.len.circular.fa.137 | Un-matched | d__Bacteria;p__Firmicutes_A;c__Clostridia;o__Oscillospirales;f__Acutalibacteraceae;g__HGM12814;s__ | d__Bacteria(100%);p__Firmicutes(100%);c__Clostridia(100%);o__Clostridiales(100%);f__Ruminococcaceae(100%);g__Neglecta(31%) | 91.846 | 1143 | Bacteria;Firmicutes;Clostridia;Oscillospirales;Ruminococcaceae;uncultured;uncultured Firmicutes bacterium |
| Rectum.out.p_ctg.gfa.seq.fa.len.circular.fa.140 | Un-matched | d__Bacteria;p__Firmicutes_A;c__Clostridia;o__Oscillospirales;f__Ruminococcaceae;g__UBA1409;s__ | d__Bacteria(100%);p__Firmicutes(100%);c__Clostridia(100%);o__Clostridiales(100%);f__Ruminococcaceae(100%);g__Ruminococcus(80%) | 95.97 | 1305 | Bacteria;Firmicutes;Clostridia;Oscillospirales;Ruminococcaceae;Ruminococcus;uncultured bacterium |
| Rectum.out.p_ctg.gfa.seq.fa.len.circular.fa.143 | Un-matched | d__Bacteria;p__Firmicutes_A;c__Clostridia;o__TANB77;f__CAG-508;g__CAG-273;s__ | d__Bacteria(100%);p__Firmicutes(100%);c__Clostridia(99%);o__Clostridiales(99%);f__Ruminococcaceae(97%);g__Petroclostridium(44%) | 96.609 | 1347 | Bacteria;Firmicutes;Clostridia;Clostridia UCG-014;uncultured bacterium |
| Rectum.out.p_ctg.gfa.seq.fa.len.circular.fa.147 | Matched | d__Bacteria;p__Firmicutes_A;c__Clostridia;o__TANB77;f__CAG-508;g__CAG-269;s__ | d__Bacteria(100%);p__Firmicutes(100%);c__Clostridia(99%);o__Clostridiales(99%);f__Ruminococcaceae(98%);g__Pseudoclostridium(45%) | 96.806 | 1357 | Bacteria;Firmicutes;Clostridia;Clostridia UCG-014;uncultured bacterium |
| Rectum.out.p_ctg.gfa.seq.fa.len.circular.fa.152 | Un-matched | d__Bacteria;p__Firmicutes;c__Bacilli;o__RF39;f__UBA660;g__CAG-451;s__ | d__Bacteria(100%);p__Firmicutes(91%);c__Erysipelotrichia(39%);o__Erysipelotrichales(39%);f__Erysipelotrichaceae(39%);g__Amedibacillus(14%) | 95.055 | 1303 | Bacteria;Firmicutes;Bacilli;RF39;uncultured bacterium |
| Rectum.out.p_ctg.gfa.seq.fa.len.circular.fa.156 | Matched | d__Bacteria;p__Firmicutes_A;c__Clostridia;o__Oscillospirales;f__Oscillospiraceae;g__Flavonifractor;s__ | d__Bacteria(100%);p__Firmicutes(100%);c__Clostridia(100%);o__Clostridiales(100%);f__Ruminococcaceae(100%);g__Intestinimonas(98%) | 96.296 | 1344 | Bacteria;Firmicutes;Clostridia;Oscillospirales;Oscillospiraceae;Pseudoflavonifractor;uncultured Flavonifractor sp. |
| Rectum.out.p_ctg.gfa.seq.fa.len.circular.fa.184 | Un-matched | d__Bacteria;p__Firmicutes_A;c__Clostridia;o__TANB77;f__CAG-508;g__CAG-269;s__ | d__Bacteria(100%);p__Firmicutes(100%);c__Clostridia(99%);o__Clostridiales(99%);f__Ruminococcaceae(96%);g__Pseudoclostridium(52%) | 96.346 | 1336 | Bacteria;Firmicutes;Clostridia;Clostridia UCG-014;uncultured bacterium |
| Rectum.out.p_ctg.gfa.seq.fa.len.circular.fa.192 | Un-matched | d__Bacteria;p__Firmicutes;c__Bacilli;o__RF39;f__UBA660;g__CAG-451;s__ | d__Bacteria(100%);p__Firmicutes(95%);c__Erysipelotrichia(41%);o__Erysipelotrichales(41%);f__Erysipelotrichaceae(41%);g__Amedibacillus(15%) | 94.925 | 1297 | Bacteria;Firmicutes;Bacilli;RF39;uncultured bacterium |
| Rectum.out.p_ctg.gfa.seq.fa.len.linear.fa.bin.1027 | Matched | d__Bacteria;p__Firmicutes_A;c__Clostridia;o__Lachnospirales;f__Lachnospiraceae;g__Catenibacillus;s__ | d__Bacteria(100%);p__Firmicutes(100%);c__Clostridia(100%);o__Clostridiales(100%);f__Lachnospiraceae(100%);g__Caecibacterium(51%) | 95 | 1280 | Bacteria;Firmicutes;Clostridia;Lachnospirales;Lachnospiraceae;Catenibacillus;uncultured bacterium |
| Rectum.out.p_ctg.gfa.seq.fa.len.linear.fa.bin.1084 | Matched | d__Bacteria;p__Firmicutes_A;c__Clostridia;o__Lachnospirales;f__Lachnospiraceae;g__Enterocloster;s__ | d__Bacteria(100%);p__Firmicutes(100%);c__Clostridia(100%);o__Clostridiales(100%);f__Lachnospiraceae(100%);g__Enterocloster(60%) | 96.467 | 1313 | Bacteria;Firmicutes;Clostridia;Lachnospirales;Lachnospiraceae;uncultured;uncultured bacterium |
| Rectum.out.p_ctg.gfa.seq.fa.len.linear.fa.bin.1305 | Matched | d__Bacteria;p__Firmicutes_A;c__Clostridia;o__Oscillospirales;f__Oscillospiraceae;g__NK3B98;s__ | d__Bacteria(100%);p__Firmicutes(100%);c__Clostridia(100%);o__Clostridiales(100%);f__Ruminococcaceae(100%);g__Intestinimonas(49%) | 95.779 | 1280 | Bacteria;Firmicutes;Clostridia;Oscillospirales;Oscillospiraceae;Colidextribacter;uncultured bacterium |
| Rectum.out.p_ctg.gfa.seq.fa.len.linear.fa.bin.1343 | Un-matched | d__Bacteria;p__Firmicutes_A;c__Clostridia;o__Peptostreptococcales;f__Anaerovoracaceae;g__UBA1191;s__ | d__Bacteria(100%);p__Firmicutes(100%);c__Clostridia(100%);o__Clostridiales(100%);f__Clostridiales_Incertae Sedis XIII(92%);g__Ihubacter(75%) | 95.203 | 1263 | Bacteria;Firmicutes;Clostridia;Peptostreptococcales-Tissierellales;Anaerovoracaceae;Family XIII AD3011 group;uncultured bacterium |
| Rectum.out.p_ctg.gfa.seq.fa.len.linear.fa.bin.1701 | Un-matched | d__Bacteria;p__Firmicutes;c__Bacilli;o__RF39;f__UBA660;g__CAG-451;s__ | d__Bacteria(100%);p__Firmicutes(90%);c__Erysipelotrichia(47%);o__Erysipelotrichales(47%);f__Erysipelotrichaceae(47%);g__Amedibacillus(20%) | 94.863 | 1294 | Bacteria;Firmicutes;Bacilli;RF39;uncultured bacterium |
| Rectum.out.p_ctg.gfa.seq.fa.len.linear.fa.bin.284 | Un-matched | d__Bacteria;p__Actinobacteriota;c__Coriobacteriia;o__Coriobacteriales;f__Eggerthellaceae;g__Adlercreutzia;s__ | d__Bacteria(100%);p__Actinobacteria(100%);c__Coriobacteriia(100%);o__Eggerthellales(100%);f__Eggerthellaceae(100%);g__Adlercreutzia(99%) | 94.6 | 1248 | Bacteria;Actinobacteriota;Coriobacteriia;Coriobacteriales;Eggerthellaceae;Enterorhabdus;Enterorhabdus caecimuris B7 |
| Rectum.out.p_ctg.gfa.seq.fa.len.linear.fa.bin.312 | Un-matched | d__Bacteria;p__Firmicutes_A;c__Clostridia;o__TANB77;f__CAG-508;g__CAG-269;s__ | d__Bacteria(100%);p__Firmicutes(100%);c__Clostridia(100%);o__Clostridiales(100%);f__Ruminococcaceae(97%);g__Anaerobacterium(33%) | 96.485 | 1345 | Bacteria;Firmicutes;Clostridia;Clostridia UCG-014;uncultured bacterium |
| Rectum.out.p_ctg.gfa.seq.fa.len.linear.fa.bin.360 | Matched | d__Bacteria;p__Firmicutes_A;c__Clostridia;o__Lachnospirales;f__Lachnospiraceae;g__Mediterraneibacter;s__ | d__Bacteria(100%);p__Firmicutes(100%);c__Clostridia(100%);o__Clostridiales(100%);f__Lachnospiraceae(100%);g__Mediterraneibacter(67%) | 96.587 | 1303 | Bacteria;Firmicutes;Clostridia;Lachnospirales;Lachnospiraceae;[Ruminococcus] torques group;uncultured bacterium |
| Rectum.out.p_ctg.gfa.seq.fa.len.linear.fa.bin.494 | Matched | d__Bacteria;p__Firmicutes_A;c__Clostridia;o__Oscillospirales;f__Oscillospiraceae;g__RUG678;s__ | d__Bacteria(100%);p__Firmicutes(100%);c__Clostridia(100%);o__Clostridiales(100%);f__Ruminococcaceae(100%);g__Sporobacter(84%) | 95.71 | 1312 | Bacteria;Firmicutes;Clostridia;Oscillospirales;Oscillospiraceae;NK4A214 group;uncultured bacterium |
| Rectum.out.p_ctg.gfa.seq.fa.len.linear.fa.bin.509 | Matched | d__Bacteria;p__Firmicutes_A;c__Clostridia;o__Monoglobales;f__Monoglobaceae;g__Monoglobus;s__ | d__Bacteria(100%);p__Firmicutes(100%);c__Clostridia(100%);o__Clostridiales(100%);f__Ruminococcaceae(100%);g__Monoglobus(100%) | 96.433 | 1349 | Bacteria;Firmicutes;Clostridia;Monoglobales;Monoglobaceae;Monoglobus;gut metagenome |
| Rectum.out.p_ctg.gfa.seq.fa.len.linear.fa.bin.515 | Un-matched | d__Bacteria;p__Firmicutes_A;c__Clostridia;o__TANB77;f__CAG-465;g__CAG-465;s__ | d__Bacteria(100%);p__Firmicutes(100%);c__Clostridia(100%);o__Clostridiales(100%);f__Ruminococcaceae(79%);g__Pseudoclostridium(30%) | 93.315 | 1168 | Bacteria;Firmicutes;Clostridia;Clostridia UCG-014;uncultured bacterium |
| Rectum.out.p_ctg.gfa.seq.fa.len.linear.fa.bin.590 | Un-matched | d__Bacteria;p__Firmicutes_A;c__Clostridia;o__Lachnospirales;f__Lachnospiraceae;g__Anaerobutyricum;s__ | d__Bacteria(100%);p__Firmicutes(100%);c__Clostridia(100%);o__Clostridiales(100%);f__Lachnospiraceae(100%);g__Anaerobutyricum(100%) | 96.833 | 1341 | Bacteria;Firmicutes;Clostridia;Lachnospirales;Lachnospiraceae;[Eubacterium] hallii group;uncultured bacterium |
| Rectum.out.p_ctg.gfa.seq.fa.len.linear.fa.bin.655 | Un-matched | d__Bacteria;p__Firmicutes;c__Bacilli;o__RF39;f__UBA660;g__CAG-460;s__ | d__Bacteria(100%);p__Tenericutes(51%);c__Mollicutes(51%);o__Entomoplasmatales(47%);f__Entomoplasmataceae(47%);g__Entomoplasma(47%) | 96.907 | 1407 | Bacteria;Firmicutes;Bacilli;RF39;gut metagenome |
| Rectum.out.p_ctg.gfa.seq.fa.len.linear.fa.bin.732 | Un-matched | d__Bacteria;p__Firmicutes_A;c__Clostridia;o__Oscillospirales;f__Oscillospiraceae;g__Flavonifractor;s__ | d__Bacteria(100%);p__Firmicutes(100%);c__Clostridia(100%);o__Clostridiales(100%);f__Ruminococcaceae(100%);g__Intestinimonas(97%) | 96.429 | 1349 | Bacteria;Firmicutes;Clostridia;Oscillospirales;Oscillospiraceae;Pseudoflavonifractor;uncultured Flavonifractor sp. |
| Rectum.out.p_ctg.gfa.seq.fa.len.linear.fa.bin.772 | Matched | d__Bacteria;p__Firmicutes_A;c__Clostridia;o__Lachnospirales;f__Lachnospiraceae;g__Eubacterium_I;s__ | d__Bacteria(100%);p__Firmicutes(100%);c__Clostridia(100%);o__Clostridiales(100%);f__Lachnospiraceae(100%);g__Lachnospiracea_incertae_sedis(58%) | 95.933 | 1315 | Bacteria;Firmicutes;Clostridia;Lachnospirales;Lachnospiraceae;Shuttleworthia;uncultured bacterium |
| Rectum.out.p_ctg.gfa.seq.fa.len.linear.fa.bin.810 | Matched | d__Bacteria;p__Firmicutes_A;c__Clostridia;o__Lachnospirales;f__Lachnospiraceae;g__UBA3402;s__ | d__Bacteria(100%);p__Firmicutes(100%);c__Clostridia(100%);o__Clostridiales(100%);f__Lachnospiraceae(100%);g__Hungatella(40%) | 94.832 | 1257 | Bacteria;Firmicutes;Clostridia;Lachnospirales;Lachnospiraceae;Lachnoclostridium;uncultured bacterium |
| Rectum.out.p_ctg.gfa.seq.fa.len.linear.fa.bin.849 | Un-matched | d__Bacteria;p__Firmicutes_A;c__Clostridia;o__TANB77;f__CAG-508;g__CAG-269;s__ | d__Bacteria(100%);p__Firmicutes(100%);c__Clostridia(100%);o__Clostridiales(100%);f__Ruminococcaceae(99%);g__Pseudoclostridium(48%) | 96.478 | 1342 | Bacteria;Firmicutes;Clostridia;Clostridia UCG-014;uncultured bacterium |
| Rectum.out.p_ctg.gfa.seq.fa.len.linear.fa.bin.881 | Matched | d__Bacteria;p__Firmicutes_A;c__Clostridia;o__Lachnospirales;f__Lachnospiraceae;g__Lachnoclostridium_A;s__ | d__Bacteria(100%);p__Firmicutes(100%);c__Clostridia(100%);o__Clostridiales(100%);f__Lachnospiraceae(100%);g__Clostridium XlVa(81%) | 95.238 | 1294 | Bacteria;Firmicutes;Clostridia;Lachnospirales;Lachnospiraceae;uncultured;Clostridium sp. AT5 |
