# Supplementary material for: Improved microbial genomes and gene catalog of the chicken gut from metagenomic sequencing of high-fidelity long reads
Source: Gigascience. 2022 Nov 18;11:giac116. doi: 10.1093/gigascience/giac116 (PMC9673493; doi:10.1093/gigascience/giac116)
Supplement: giac116_Response_to_Reviewer_Comments_Original_Submission [file giac116_response_to_reviewer_comments_original_submission.pdf]

Author's Response To Reviewer Comments

Close

Editor comments:

In addition, please register any new software application in the bio.tools and SciCrunch.org databases to receive RRID (Research Resource Identification Initiative ID) and biotoolsID identifiers, and include these in your manuscript. This will facilitate tracking, reproducibility and re-use of your tool.

Reply: we have registered new software in the SciCrunch.org databases, and included RRIDs of all the used software in our manuscript.

Reviewer reports:

Reviewer #1: The authors sequenced the chicken gut microbiome using HiFi sequencing based on PacBio Sequel II platform, and further revealed its advantages compared with traditional short-read sequencing: higher quality of assemblies for gene structures and more recovered novel genes and genomes. Although the conclusions obtained in this study are right, this work meets our expectations and is a commonsense. In addition, this study is a little bit simple because they only compare the two sequencing technologies. The authors should perform some analysis about scientific findings in chicken gut microbiome. The detailed comments are as following：

1. This paper has spent large effort to prove the advantage of the high-fidelity (HiFi) sequencing, but a lot of previous research have reported the advantage of the high-fidelity (HiFi) sequencing and it is a commonsense. What is really new in this paper? The authors should reveal the scientific findings among different chicken-gut microbiota.

Reply: Thanks to the reviewer's suggestions. As this is the first HiFi metagenome project for chicken, we have performed detailed comparisons of the HiFi metagenome data and short-read metagenome data. Although the advantage of HiFi metagenome sequencing has been shown by a few previous studies, our analysis has used different assembly method, so we think it is necessary to make detailed comparisons again, which can assure that the chicken HiFi-derived microbial genomes and gene catalogue are both in high quality.

We admit that the experiment design of this study is a bit simple. However, we think that the quick release of the generated resources, including the assembled novel strain and species genomes, as well as the predicted novel genes, will be very helpful to the scientific world and chicken production industry. We have also made several scientific findings from our metagenome data analysis, such as: (1) The dominant phyla in our HiFi assembled genomes is Firmicutes (82.5%), and the foregut is highly enriched in five genera Ligilactobacillus, Limosilactobacillus, Lactobacillus, Weissella, and Enterococcus. (2) Among the 337 species-level microbial genomes, 9 and 49 of them may belong to novel genera and species, respectively.

Now we have added some more analysis and descriptions in the manuscript, and the original Result section "Phylogeny of HiFi assembled microbial genomes and novel genomic representation"was split into two sections "Phylogeny of HiFi assembled microbial genomes and differences among intestinal compartments" and "Novel genomic representation and novel genus and species discovery". Some important revisions were listed below:

(1) In Result section "Phylogeny of HiFi assembled microbial genomes and differences among intestinal compartments", we have added:

"The remaining genomes were classified as Cyanobacteria, Proteobacteria, Desulfobacterota, Campylobacterota, Deferribacterota, Methanobacteriota, and Verrucomicrobiota.";

“The foregut contains the duodenum, jejunum, and ileum, which mainly function in feed digestion and nutrient absorption. The hindgut contains the cecum and colorectum, which function in fermentation, detoxification and recycling of residual water and salt.”;

(2) In Result section “Novel genomic representation and novel genus and species discovery”, we have added:

“Some of these new genomic sequences may have potential benefits to industry or medical applications. Lactobacillus has traditionally been used in the fermentation industry, producing lactate from raw carbohydrates and synthetic media (Sun, et al., 2015). In recent years, Lactobacillus and its close relatives Ligilactobacillus and Limosilactobacillus have also been widely adopted as probiotic supplements, either in animal feed to promote growth or human foods to improve human health (Sarao and Arora, 2017). Among our 337 assembled microbial species genomes, 3 genomes belong to Lactobacillus, 7 genomes belong to Ligilactobacillus, and 6 genomes belong to Limosilactobacillus. All these genomes have been successfully classified to the genus level, and most of the genomes were successfully classified to the species level by GTDB-Tk. However, 2 Ligilactobacillus genomes and 1 Limosilactobacillus genome have not been classified to the species level, suggesting that these 3 species-level genomes may represent novel genomic resources for probiotic development.”;

“In addition, approximately one-third of these newly discovered genera and half of these newly discovered species were not found in the short-read MAG data, suggesting that they are derived only from HiFi metagenome data, which further shows the advantage of HiFi sequencing in metagenomic studies.”;

(3) In Result section “Advantage of HiFi-derived gene catalog over gene catalogs from short-reads”, we have added a paragraph:

“By comparing the pairwise overlap at the gene sequence level, we found that 847,801 (33.8%) and 724,123 (28.9%) genes are unique in HiFi-RGC compared to CGM-RGC and GG-IGC, respectively (Figure 6c-d), suggesting that the HiFi-derived gene catalog recovered a substantial portion of the genes that were missed by short-read technologies. Because GG-IGC is more comprehensive than CGM-RGC, we considered the 724,123 (28.9%) genes in HiFi-RGC as unique genes and the remaining genes (71.1%) in HiFi-RGC as shared genes. Then, the microbial communities derived from the unique and shared genes in HiFi-RGC were compared. The results showed that 36.8% of unique genes were unclassified at the phylum level, which was obviously higher than the proportion of shared genes (24.9%), suggesting that the unique genes are enriched in unknown phyla (Figure S6).”

2. Method section: "The 30 chickens were separated into 6 groups, with each group containing 5 chickens.". However, the digesta samples of the 30 chickens were finally pooled, mixed together and analyzed based on the difference of intestinal compartment. Pls elucidate what the meaning of classification is?

Reply: The mentioned “classification” was just for the convenience of experimental processing, as the total volume of digesta from all chickens was large and difficult to process in one time. For each intestinal compartment， samples from all the 30 chickens were pooled altogether in two steps of pooling (digesta pooling and extracted DNA pooling). The manuscript has been revised with more detailed and clear description：

“Mainly due to the volume of digesta, it was difficult to process all of the samples at one time. For the convenience of processing, the duodenum digesta from every 5 chickens were pooled together and then washed for microbial cell enrichment and DNA extraction. After processing all duodenum samples, the metagenomic DNA was finally pooled, and further purified with VAHTS DNA Clean Beads (N411-02, Vazyme). The metagenomic DNA samples of the jejunum, ileum, cecum and colorectum were processed in the same way, except that for the cecum, due to its relatively high microbial density, only a subfraction of the pooled and thoroughly mixed digesta was used for microbial cell enrichment and DNA extraction.”

3. The line number should be added in the manuscript, and English needs to be polished throughout the MS.

Reply: We have added the line number and polished the English by a native speaker.

Reviewer #2: The authors present work that expands the databases of chicken microbiome genomes with high quality assemblies and represents a valuable resource. The authors also demonstrate a step towards moving from short read assembly of metagenomes towards long read assemblies, which is undoubtedly the direction the field should be moving in.

While I expect these genomes to be high quality and not heavily impacted by indels given the accuracy of hifi and the high completeness statistics, a common criticism of long-reads is the error rate. Have the authors considered demonstrating that this is not impacting their high quality genomes through, for example, the ideel tool or some other assessment of error rate in the assemblies? This may strengthen the message that long reads are suitable for this kind of work, particularly if the result is independent of the depth of coverage over each species.

Reply: Before metagenome assembly, we have pre-filtered low quality HiFi reads, and only HiFi reads with quality over Q20 were used for metagenome assembly. The high base quality of input reads makes sure that the high base quality of the contig assembly.

In respect to the hifiasm-meta algorithm, it first performs HiFi-reads error correction from overlapped reads, which is equivalent to call the consensus sequences from multiple aligned reads. So, higher reads coverage will improve the error correction of the HiFi reads, and finally improve the base accuracy of the contig assemblies. In the hifiasm-meta paper (Feng, X., et al. Metagenome assembly of high-fidelity long reads with hifiasm-meta. Nat Methods 2022), Extended Data Fig.2 shows Yak quality value (QV) score correlated with contig coverage, and higher coverage will result in higher Yak QV. Yak QV is a rough estimation of the single base accuracy, including both mismatch and indel errors.

Here, we performed the same analysis using our chicken metagenome contig data, and obtained a similar result, which was shown in Figure 2d: Correlation plot of YAK quality score (QV) and coverage depth, using contigs with length over 100 Kb from all intestinal compartments. The K-mer frequency was calculated with parameters "yak count -b37 -t48" and yak QV was calculated with parameters "yak qv -t80 -p -K3.2g -l100k". The red marker line indicates that higher coverage depth will improve the single base quality of the contig sequences.

We have also added descriptions in the maintext (Results/Longer contigs of the chicken metagenome assembled from high-fidelity long reads): "Moreover, the coverage depth is positively related with the single-base quality values, indicating that higher coverage depth will improve the single-base accuracy of the contig sequences (Figure 2d).".

In addition, we also used the checkM completeness to evaluate the single base accuracy of the assembled genomes: We added a supplementary figure S5: Correlation plot of genome coverage depth and checkM completeness score. The 187 circular genomes out of 337 non-redundant species-level genomes were used here. Considering all these genomes have complete genome assemblies, the difference of checkM completeness scores should only be caused by the single base accuracy, due to the marker gene prediction method adopted by checkM. Genome assemblies with higher single base accuracy will have higher checkM completeness values.The plots clearly shows that higher coverage depth will result in higher checkM completeness scores, indicating that higher coverage depth will improve the single base accuracy of genome assemblies.

We also added descriptions in the maintext "Using the 187 circular species-level genomes, which all have complete genome assemblies, we showed that higher coverage depth is positively correlated with CheckM completeness score, indicating that a higher coverage depth will improve the single-base accuracy of the genome assemblies (Figure S5)."

Can the authors elaborate on the methods they used to compare the existing gene catalogs to their own as this appears to not be described in the methods section?

Reply: We have added the methods for comparing the gene catalogs in Methods/ Non-redundant gene catalog construction:

To compare the overlap of our gene catalog (HiFi-RGC) with two published chicken gut metagenome gene catalogs (CGM-RGC and GG-IGC) (Feng, et al., 2021; Huang, et al., 2018), pairwise alignments of HiFi-RGC to CGM-RGC and HiFi-RGC to GG-IGC were performed using BLAT (BLAT, RRID:SCR_011919) (Kent, 2002) with identity ≥ 95% and overlap ≥ 90% of the shorter genes as the criteria for shared genes.

Can the authors include in the supplement some stats on the quantity and quality of DNA extracted from each sample type? It is very useful for people considering using the same protocol especially when long-read sequencing typically requires high levels of high quality starting material.

Reply: We have added the quantity and quality information in Table S1 and Figure S1. The DNA quality and quantity were measured by Invitrogen Qubit 4 Fluorometer with Qubit™ dsDNA BR(Invitrogen, Q32850) and by Nanodrop 2000c Microvolume Spectrophotometer (Table S1). The integrity of DNA was evaluated on field electrophoresis agarose gels (Figure S1).

Table S1. Quality and Quantity assessment of the extracted DNA

Figure S1. Agarose gel electrophoresis. (a) DC electrophoresis (0.7% gel, 100V, 1h) for Duodenum microbiota DNA (3011A); M1 15kb DNA Marker(15000、10000、7500、5000、2500、1000、250bp); M2 λDNA /HindIII(23130、9416、6557、4361、2322、2027、564bp). (b) Pulse electrophoresis (0.7% gel, pulse 5~80kb, 16h) for Duodenum microbiota DNA (3011A); M1 15kb DNA Marker; M2 λDNA /HindIII; (c) DC electrophoresis (1% gel, 180V, 20min) for Jejunum microbiota DNA (lane 1), Ileum microbiota DNA (lane 2), Cecum microbiota DNA (lane 3), and Colorectum microbiota DNA (lane 4); S stantard sample (50ng); M-1 trans 2k plus; M-2 trans 15k plus. (d) Pulse electrophoresis (0.8% gel, pulse 5~80kb, 17h) for Jejunum microbiota DNA (lane 1), Ileum microbiota DNA (lane 2), Cecum microbiota DNA (lane 3), and Colorectum microbiota DNA (lane 4); M 48kb DNA Extension Ladder. In summary, the microbiota DNA from all intestinal fragments are intact except for the Duodenum, which is slightly degraded. The microbiota DNA from all intestinal fragments are qualified for HiFi sequencing.

The authors state that computational limitations prevented them from doing a co-assembly, but did they include coverage data from multiple gut regions while binning their non-circular contigs with metabat2? Using many samples from different individuals and regions of the gut aligned to the contigs you are binning can improve the outcome from binning and uses more data without requiring the extreme computational power of the assembly stage.

Reply: Thanks for the reviewer's suggestion. We agreed that using many samples from different individuals and regions of the gut may improve the binning process. If we have taken a co-assembly of all the intestinal data and generated one contig data set, then that will be a certain choice.

However, a big limitation in metagenome assembly is the computer memory, for example, only the assembly of cecum data needs about 800 G memory, and it will take over 3000 G memory for a co-assembly of all the 5 intestinal fragments. Considering that the maximum memory of our computer server is 1000 G, we assembled each of the 5 intestinal fragments separately, resulting in 5 hifiasm-meta contig data sets. Then, a major task is to merge them into one assembly result. If we merge all the contigs firstly, then the linkage and depth information from the hifiasm-meta GFA files will be lost.

To fully utilize the linkage and depth information from the hifiasm-meta GFA file, we decided to get genome-level assembly for each of the 5 intestinal fragment firstly, including 3 parts: (1) the circular contigs were taken as complete genome; (2) "tangled"circular were re-assembled into non-redundant contig assembly; (3) linear contig were binned by Metabat2 using the depth information obtained from the hifiasm-meta GFA file of the corresponding intestinal fragment. In this way, the contigs and the depth information matched well. Finally, the genome-level assemblies from all the 5 intestinal fragments were merged into a non-redundant genome-level assembly. We think this strategy is more suitable for

our data analysis. Although the results here may not be the best, but it should be reliable.

There are a couple of instances of GTDB-tk being written as GTDT-tk, please double check this is correct throughout.

Reply: We have corrected all “GTDT-tk”to “GTDB-tk”.

I find the panel order in figure 5 to be unintuitive, I suggest that the panels below A and B go in order from left to right rather than top to bottom.

Reply: We have re-ordered the panel order of figure 5, let c, d, e in one row, and f, g, h in another row.

The last paragraph of page 7 is a little fiddly to follow, it might be better presented as a table or just referring the reader to see the data in the figure.

Reply: We have added a supplementary Table (Table S5) to make it easy to follow:

Table S5. Unclassified number of genomes at each taxonomic level
Note: Here the 337 species-level microbial genomes were used as input, and the unclassified number of genomes at each taxonomic levels (family, genus, species) were shown by three classification methods: (1) GTDB-tk alone; (2) GTDB-tk and Ribosomal Database Project (RDP) Classifier; (3) GTDB-tk and Ribosomal Database Project (RDP) Classifier and alignments to the Silva 16S rRNA database. The last method classified the maximum number of genomes, and only 9 and 49 genomes failed to be classified into known genus and species, indicating that they may be novel genus and species which haven’t been reported before.

Page 7 refers to "figure c-d" without a figure number.

Reply: "figure c-d" has been changed to “"Figure 6c-d"”.

I like figure 7, but a couple of parts are difficult to interpret. The circles and squares are not very distinct, is it possible for the squares to be another shape that is less similar to the circles? With the aid of zooming in it is possible to read the labels on the depth tracks, but for convenience I would recommend also listing the labels in order in the caption.

Reply: We have used hollow circles to replace the squares, and used solid circles to replace the circles. We also listed the labels in order in the caption “From inner to outer: duodenum, jejunum, ileum, cecum, and colorectum.”.

I find this sentence on page 10 a bit ambiguous and suggest adjusting for clarification "Finally, the combined DNA samples from 30 chicken individuals for duodenum, jejunum, ileum, cecum, and colorectum were generated independently." (i.e. what is combined and what is independent)

Reply: For each intestinal compartment, all the extracted DNA from all chickens were combined. The digesta samples from different intestinal compartment were processed separately. Finally， there were only 5 metagenomic DNA samples of duodenum, jejunum, ileum, cecum, and colorectum. The manuscript was revised for a better clarification：

“Mainly due to the volume of digesta, it was difficult to process all of the samples at one time. For the convenience of processing, the duodenum digesta from every 5 chickens were pooled together and then washed for microbial cell enrichment and DNA extraction. After processing all duodenum samples, the metagenomic DNA was finally pooled, and further purified with VAHTS DNA Clean Beads (N411-02, Vazyme). The metagenomic DNA samples of the jejunum, ileum, cecum and colorectum were processed in the same way, except that for the cecum, due to its relatively high microbial density, only a

subfraction of the pooled and thoroughly mixed digesta was used for microbial cell enrichment and DNA extraction."

Can the authors clarify in the methods section the method of bead beating used in the protocol, for reproducibility.

Reply: The details about bead beating were added into the manuscript : "For the bead beating and lysis options of the DNeasy PowerSoil Pro kit, we added approximately 200 mg of the enriched cells and 800 uL of Solution CD1 into each PowerBead Pro Tube. The tubes were vortexed briefly to mix and incubated at 65 °C for 10 min before the bead beating step. Then, the tubes were placed horizontally and properly balanced on a Vortex Adapter for 24 (1.5–2.0 ml) tubes (QIAGEN, 13000-V1-24) on a Kylin-Bell VORTEX-6. The samples were vortexed in the tubes at maximum speed for 10 min. To ensure the efficiency of the homogenization step, fewer than 12 tubes were vortexed at one time. All the other steps were carried out according to the manufacturer's standard protocol."

Close
