# Supplementary material for: Improved microbial genomes and gene catalog of the chicken gut from metagenomic sequencing of high-fidelity long reads
Source: Gigascience. 2022 Nov 18;11:giac116. doi: 10.1093/gigascience/giac116 (PMC9673493; doi:10.1093/gigascience/giac116)
Supplement: giac116_Response_to_Reviewer_Comments_Revision_1 [file giac116_response_to_reviewer_comments_revision_1.pdf]

Author's Response To Reviewer Comments

Close

I've made some revisions and added some comments in your manuscript, please check all changes and resolve all my comments.
Reply: Thanks for the revisions of our manuscript. We have checked all the changes and reformatted the references and URLs to the GigaScience style. We also searched the two questioned RRIDs, RRID:SCR_022773 and RRID:SCR_022774, and found they both can be found in the RRID database （ https://www.rrids.org/）, but were displayed at the second item. The searching results are listed below:

RRID:SCR_022773
https://sourceforge.net/projects/rdp-classifier/
Software tool as naive Bayesian classifier that can rapidly and accurately provide taxonomic assignments from domain to genus, with confidence estimates for each assignment.
Proper citation: RDP Classifier (RRID:SCR_022773)

RRID:SCR_022774
https://github.com/ablab/viralVerify
Software viral contig verification tool. Classifies contigs as viral, non-viral or uncertain, based on gene content. Also for non-viral contigs it can optionally provide plasmid/non-plasmid classification.
Proper citation: viralVerify (RRID:SCR_022774)

Also please meet the following requirements:
1.Figures should be provided as separate files, not embedded in the main manuscript file.
2.Each figure of a manuscript should be submitted as a single file that fits on a single page in portrait format.
3.Figure titles (max 15 words) and legends (max 300 words) should be provided in the main manuscript, not in the graphic file.
Reply: Each figure has been submitted as single file, and the figure titles and legends meet the requirements.

4.All references, including URLs, must be numbered consecutively, in square brackets, in the order in which they are cited in the text, followed by any in tables or legends.
Reply: We have reformatted all the reference papers and URLs.

Reviewer reports:
Reviewer #1: The revised manuscript has addressed all my concerns except a new nomenclature table for all abbreviations.
Reply: We have provided a nomenclature list for all abbreviations.

Close
