# Supplementary material for: Improved microbial genomes and gene catalog of the chicken gut from metagenomic sequencing of high-fidelity long reads
Source: Gigascience. 2022 Nov 18;11:giac116. doi: 10.1093/gigascience/giac116 (PMC9673493; doi:10.1093/gigascience/giac116)
Supplement: giac116_Reviewer_1_Report_Original_Submission — Fei Chen -- 8/18/2022 Reviewed [file giac116_reviewer_1_report_original_submission.pdf]

Reviewer Report

Title: Improved microbial genomes and gene catalog of chicken gut from metagenomic sequencing of high-fidelity long reads

Version: Original Submission **Date:** 8/18/2022

Reviewer name: Fei Chen

Reviewer Comments to Author:

The authors sequenced the chicken gut microbiome using HiFi sequencing based on PacBio Sequel II platform, and further revealed its advantages compared with traditional short-read sequencing: higher quality of assemblies for gene structures and more recovered novel genes and genomes. Although the conclusions obtained in this study are right, this work meets our expectations and is a commonsense. In addition, this study is a little bit simple because they only compare the two sequencing technologies. The authors should perform some analysis about scientific findings in chicken gut microbiome. The detailed comments are as followingï¼š
1. This paper has spent large effort to prove the advantage of the high-fidelity (HiFi) sequencing, but a lot of previous research have reported the advantage of the high-fidelity (HiFi) sequencing and it is a commonsense. What is really new in this paper? The authors should reveal the scientific findings among different chicken-gut microbiota.
2. Method section: "The 30 chickens were separated into 6 groups, with each group containing 5 chickens.". However, the digesta samples of the 30 chickens were finally pooled, mixed together and analyzed based on the difference of intestinal compartment. Pls elucidate what the meaning of classification is?
3. The line number should be added in the manuscript, and English needs to be polished throughout the MS.

Methods

Are the methods appropriate to the aims of the study, are they well described, and are necessary controls included? Choose an item.

Conclusions

Are the conclusions adequately supported by the data shown? Choose an item.

Reporting Standards

Does the manuscript adhere to the journal’s guidelines on minimum standards of reporting? Choose an item.

Choose an item.

Statistics

Are you able to assess all statistics in the manuscript, including the appropriateness of statistical tests used? Choose an item.

Quality of Written English

Please indicate the quality of language in the manuscript: Choose an item.

Declaration of Competing Interests

Please complete a declaration of competing interests, considering the following questions:

- Have you in the past five years received reimbursements, fees, funding, or salary from an organisation that may in any way gain or lose financially from the publication of this manuscript, either now or in the future?
- Do you hold any stocks or shares in an organisation that may in any way gain or lose financially from the publication of this manuscript, either now or in the future?
- Do you hold or are you currently applying for any patents relating to the content of the manuscript?
- Have you received reimbursements, fees, funding, or salary from an organization that holds or has applied for patents relating to the content of the manuscript?
- Do you have any other financial competing interests?
- Do you have any non-financial competing interests in relation to this paper?

If you can answer no to all of the above, write 'I declare that I have no competing interests' below. If your reply is yes to any, please give details below.

I declare that I have no competing interests.

I agree to the open peer review policy of the journal. I understand that my name will be included on my report to the authors and, if the manuscript is accepted for publication, my named report including any attachments I upload will be posted on the website along with the authors' responses. I agree for my report to be made available under an Open Access Creative Commons CC-BY license (http://creativecommons.org/licenses/by/4.0/). I understand that any comments which I do not wish to be included in my named report can be included as confidential comments to the editors, which will not be published.

Choose an item.

To further support our reviewers, we have joined with Publons, where you can gain additional credit to further highlight your hard work (see: https://publons.com/journal/530/gigascience). On publication of this paper, your review will be automatically added to Publons, you can then choose whether or not to claim your Publons credit. I understand this statement.

Yes Choose an item.
