# Supplementary material for: Improved microbial genomes and gene catalog of the chicken gut from metagenomic sequencing of high-fidelity long reads
Source: Gigascience. 2022 Nov 18;11:giac116. doi: 10.1093/gigascience/giac116 (PMC9673493; doi:10.1093/gigascience/giac116)
Supplement: giac116_Reviewer_2_Report_Original_Submission — Amanda Warr -- 9/12/2022 Reviewed [file giac116_reviewer_2_report_original_submission.pdf]

Reviewer Report

Title: Improved microbial genomes and gene catalog of chicken gut from metagenomic sequencing of high-fidelity long reads

Version: Original Submission **Date:** 9/12/2022

Reviewer name: Amanda Warr

Reviewer Comments to Author:

The authors present work that expands the databases of chicken microbiome genomes with high quality assemblies and represents a valuable resource. The authors also demonstrate a step towards moving from short read assembly of metagenomes towards long read assemblies, which is undoubtedly the direction the field should be moving in.
While I expect these genomes to be high quality and not heavily impacted by indels given the accuracy of hifi and the high completeness statistics, a common criticism of long-reads is the error rate. Have the authors considered demonstrating that this is not impacting their high quality genomes through, for example, the ideel tool or some other assessment of error rate in the assemblies? This may strengthen the message that long reads are suitable for this kind of work, particularly if the result is independent of the depth of coverage over each species.
Can the authors elaborate on the methods they used to compare the existing gene catalogs to their own as this appears to not be described in the methods section?
Can the authors include in the supplement some stats on the quantity and quality of DNA extracted from each sample type? It is very useful for people considering using the same protocol especially when long-read sequencing typically requires high levels of high quality starting material.
The authors state that computational limitations prevented them from doing a co-assembly, but did they include coverage data from multiple gut regions while binning their non-circular contigs with metabat2? Using many samples from different individuals and regions of the gut aligned to the contigs you are binning can improve the outcome from binning and uses more data without requiring the extreme computational power of the assembly stage.
There are a couple of instances of GTDB-tk being written as GTDT-tk, please double check this is correct throughout.
I find the panel order in figure 5 to be unintuitive, I suggest that the panels below A and B go in order from left to right rather than top to bottom.
The last paragraph of page 7 is a little fiddly to follow, it might be better presented as a table or just referring the reader to see the data in the figure.
Page 7 refers to "figure c-d" without a figure number.
I like figure 7, but a couple of parts are difficult to interpret. The circles and squares are not very distinct, is it possible for the squares to be another shape that is less similar to the circles? With the aid of zooming in it is possible to read the labels on the depth tracks, but for convenience I would recommend also listing the labels in order in the caption.
I find this sentence on page 10 a bit ambiguous and suggest adjusting for clarification "Finally, the

combined DNA samples from 30 chicken individuals for duodenum, jejunum, ileum, cecum, and colorectum were generated independently." (i.e. what is combined and what is independent)
Can the authors clarify in the methods section the method of bead beating used in the protocol, for reproducibility.

Methods

Are the methods appropriate to the aims of the study, are they well described, and are necessary controls included? Choose an item.

Conclusions

Are the conclusions adequately supported by the data shown? Choose an item.

Reporting Standards

Does the manuscript adhere to the journal's guidelines on minimum standards of reporting? Choose an item.

Choose an item.

Statistics

Are you able to assess all statistics in the manuscript, including the appropriateness of statistical tests used? Choose an item.

Quality of Written English

Please indicate the quality of language in the manuscript: Choose an item.

Declaration of Competing Interests

Please complete a declaration of competing interests, considering the following questions:

- Have you in the past five years received reimbursements, fees, funding, or salary from an organisation that may in any way gain or lose financially from the publication of this manuscript, either now or in the future?
- Do you hold any stocks or shares in an organisation that may in any way gain or lose financially from the publication of this manuscript, either now or in the future?
- Do you hold or are you currently applying for any patents relating to the content of the manuscript?
- Have you received reimbursements, fees, funding, or salary from an organization that holds or has applied for patents relating to the content of the manuscript?
- Do you have any other financial competing interests?
- Do you have any non-financial competing interests in relation to this paper?

If you can answer no to all of the above, write 'I declare that I have no competing interests' below. If your reply is yes to any, please give details below.

I declare that I have no competing interests

I agree to the open peer review policy of the journal. I understand that my name will be included on my report to the authors and, if the manuscript is accepted for publication, my named report including any attachments I upload will be posted on the website along with the authors' responses. I agree for my report to be made available under an Open Access Creative Commons CC-BY license (http://creativecommons.org/licenses/by/4.0/). I understand that any comments which I do not wish to be included in my named report can be included as confidential comments to the editors, which will not be published.

Choose an item.

To further support our reviewers, we have joined with Publons, where you can gain additional credit to further highlight your hard work (see: https://publons.com/journal/530/gigascience). On publication of this paper, your review will be automatically added to Publons, you can then choose whether or not to claim your Publons credit. I understand this statement.

Yes Choose an item.
